# Encapsulation of phenolic acids into cyclodextrins: A global statistical analysis of the effects of pH, temperature and concentrations on binding constants measured by ACE methods

**DOI:** 10.1002/elps.202200075

**Published:** 2022-07-10

**Authors:** Amra Aksamija, Valérie Tomao, Olivier Dangles, Raphaël Plasson

**Affiliations:** ^1^ Department of Chemistry, Avignon University, CNRS, UMR5247 CBSA/IBMM Avignon France; ^2^ Department of Chemistry, Avignon University, INRAE, UMR408 SQPOV Avignon France

**Keywords:** affinity capillary electrophoresis, multiparameter analysis, parameter optimization, polyphenol–cyclodextrin inclusion complex, thermodynamic parameters

## Abstract

Affinity capillary electrophoresis was used for the simultaneous measurement of the p*K*_a_ values and of the binding constants relative to the encapsulation of naturally occurring phenolic acids (rosmarinic and caffeic acids) with cyclodextrins. A thorough study as a function of pH and temperature was coupled to a detailed statistical analysis of the resulting experimental data. A step‐by‐step curve fitting process was sufficient for obtaining individual binding constant for each experimental condition, but the influence of temperature remained unclear. A quantitative and qualitative gain was then obtained by supplementing this initial analysis with global multiparameter optimization. This leads to the estimation of both entropy and enthalpy of reaction and to the full description of the binding reactions as a function of pH and temperature. The encapsulation was shown to be very sensitive to pH and temperature, with optimal complexation occurring at low pH and low temperature, gaining up to a factor of 3 by cooling from 36 to 15°C, and up to a factor of 10 by lowering the pH from 7 to 2.

**Abbreviations**
CD: β‐cyclodextrin
mCD: methyl‐β‐cyclodextrin

## 1 INTRODUCTION

**FIGURE 1:**
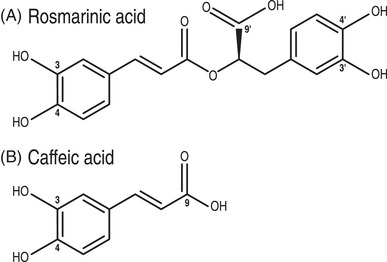
Chemical structure of rosmarinic and caffeic acids

Phenolic compounds are the largest class of secondary metabolites in plants [1]. Besides their important functions in plants (e.g., antioxidants, UV filters, pigments and copigments), they have attracted a lot of research over the last decades owing to their participation in the nutritional effects of plant‐based foods, mostly through their antioxidant and anti‐inflammatory activities [2]. They also have potential for development in the agro‐food, cosmetic and pharmaceutical industries [3, 4]. Among them, rosmarinic acid with its caffeic (3,4‐dihydroxycinnamic) and 3,4‐dihydroxyphenylacetic moieties is an important component of the rosemary (*Rosmarinus officinalis*) extracts [5] marketed for their antioxidant properties (see structure in Figure 1).

β‐Cyclodextrin (CD) is a macrocyclic oligosaccharide derived from starch with seven glucopyranose units linked by α‐1,4 glycosidic bonds [6]. It possesses a nonpolar central cavity suitable for binding small polarizable ligands with a hydrophobic character such as phenolic compounds [7, 8, 9, 10]. Through encapsulation into CD, sensitive guest molecules are typically protected against light‐induced and oxidative deterioration, thus facilitating their storage before use [11]. Their bioavailability—that is, their release in the digestive tract for subsequent intestinal absorption—may also be modulated.

The physicochemical properties of CD‐encapsulated phenolic compounds can be studied by capillary electrophoresis (CE) as an analytical tool of choice [12, 13, 14, 15]. It is, namely, well adapted for the determination of p*K*_a_ values [16, 17], as well as binding constants by affinity CE (ACE) methods [18, 19, 20]. ACE was, namely, shown to be efficient for the determination of binding constants while taking into account the variation of the protonation of the substrate as a function of pH [21, 22]. Overall, ACE methods can be used for the measurements of both binding constants and p*K*_a_ values, in precisely controlled conditions of temperature and ionic strength; the analysis can be performed simultaneously on several compounds present in a mixture, with a very low consumption of samples and buffer solutions.

The measurements of binding constants are generally performed in specific temperature and pH conditions and are, thus, difficult to compare from one work to another. Moreover, large inconsistencies are observed in the literature, with binding constants between rosmarinic acid and β‐CD reported from 100 [23, 24, 25] to 2000 M^−1^ [26, 27] in similar conditions (neutral pH, and around 25°C). In this work, the ACE method is applied to the systematic study of the encapsulation of rosmarinic (R) and caffeic (C) acids into CD and methyl‐CD (mCD, with 1.6–2.0 methyl substitution per glucopyranose unit) as a function of pH and temperature. The corresponding thermodynamic data of encapsulation were evaluated and analyzed. Two analytical methods for extracting the data were compared: a first step‐by‐step approach, and a global one. These two procedures revealed to be complementary for optimizing the determination of the studied physicochemical parameters, leading to the identification of the optimal conditions of binding and dissociation, and to evaluate the potential of CD derivatives for the effective extraction of phenolic acids.

## 2 THEORY

The full study was performed by measuring the electrophoretic mobility μ of phenolic acids in various conditions. Samples containing both rosmarinic and caffeic acids were analyzed with different buffer solutions, pH, CD and mCD concentrations, and temperatures. In all experiments, the buffer ionic strength was fixed to *I* = 10 mM.

### 2.1 Pertinent chemical equilibria

Both R and C (noted further as X) are both engaged in acid–base reactions. In the pH range studied, they are in either neutral protonated form (XH) at low pH or anionic deprotonated form (X^−^) at high pH. The electrophoretic mobility being proportional to the charge, only the anionic form possesses nonzero electrophoretic mobility. Decreasing the pH from above to below the p*K*_a_ leads to the decrease of the measured mobilities, until reaching zero when full protonation is achieved.

The complexation of X^−^ compound with cyclodextrins (noted further as L for either CD or mCD) leads to the formation of a 1 : 1 anionic complex $X^{-}\cdot L$ [23, 24]. The electrophoretic mobility being inversely proportional to the hydrodynamic radius of the compound, its value decreases upon binding to L. Based on the literature, the possible formation of 2 : 1 complexes was dismissed, as such complexes were only detected at lower pH than in our work (ca. pH 1), and for large X/L ratios [28].

The chemical reaction network is thus

(1) $$\begin{array}{c} \begin{array}{ccccccc} K_{a,X}: & \mathrm{XH} & ⇌X^{-}+H^{+} & & K_{a,\mathrm{XL}}: & \mathrm{XH}\cdot L & ⇌X^{-}\cdot L+H^{+}\\ K_{X^{-}}: & X^{-}+L & ⇌X^{-}\cdot L & & K_{\mathrm{XH}}: & \mathrm{XH}+L & ⇌\mathrm{XH}\cdot L \end{array} \end{array}$$

with L representing the free CD or mCD, $X^{-}\cdot L$ is the bound anionic form, and XH · L is the bound neutral form. These equilibrium constants are linked by the following relationship:

(2) $$\;K_{a,\mathrm{XL}}=\frac{K_{X^{-}}}{K_{\mathrm{XH}}}\;K_{a,X}$$

Only $K_{X^{-}}$, *K*_XH_ and *K*_a, X_ constants were thus considered, as *K*_a, XL_ can be calculated from them. For the sake of simplicity, *K*_a, X_ will further be referred to simply as *K*_a_.

Finally, the different concentrations are linked by the following equations:

(3) $$\begin{array}{ccc} K_{a,X} & = & \frac{\left[X^{-}\right]\left[H^{+}\right]}{\left[\mathrm{XH}\right]}\\ K_{X^{-}} & = & \frac{\left[X^{-}\cdot L\right]}{\left[X^{-}\right]\left[L\right]}\\ K_{\mathrm{XH}} & = & \frac{\left[\mathrm{XH}\cdot L\right]}{\left[\mathrm{XH}\right]\left[L\right]}\\ x & = & \left[\mathrm{XH}\right]+\left[X^{-}]+[\mathrm{XH}\cdot L]+[X^{-}\cdot L\right]\\ c & = & \left[L\right]+\left[\mathrm{XH}\cdot L\right]+\left[X^{-}\cdot L\right] \end{array}$$

with *x* and *c*, the total concentrations are, respectively, X and L. In most cases, the condition x≪c (large excess of cyclodextrins) is satisfied; the last equation can thus be simplified as c≈[L].

It shall be noted that these constants, being written as a function of the concentrations rather than the activities, are apparent constants at the studied ionic strength of 10 mM. The binding constants *K*_XH_ should not depend on the ionic strength, as all products and reactants are not charged. As for $K_{X^{-}}$, the charge of the corresponding reactants and products are nonzero but identical; as a consequence, only a small influence of the ionic strength on this constant shall be expected. Finally, *K*_a_ values will depend more strongly on the ionic strength; a correction will have to be done to extrapolate their value at infinite dilution, as detailed further.

### 2.2 Electrophoretic mobilities

#### 2.2.1 General expression

All the chemical reactions (binding and proton transfers) are very fast compared to the analysis time. The measured electrophoretic mobility of compound X, thus, reflects the average mobility of all species at a given pH, that is, X^−^, XH, $X^{-}\cdot L$ and XH · L. As only X^−^ and $X^{-}\cdot L$ are charged, the apparent electrophoretic mobility can be expressed as

(4) $$\mu =\frac{\left[X^{-}\right]\cdot \mu_{X^{-}}+\left[X^{-}\cdot L\right]\cdot \mu_{X^{-}L}}{x}$$

with *x*, the total concentration is X, and $\mu_{X^{-}}$ and $\mu_{X^{-}L}$ are the electrophoretic mobilities of, respectively, X^−^ and $X^{-}\cdot L$.

#### 2.2.2 Ionic strength and temperature effects

When working at very low ionic strength, the electrophoretic mobility can be simply expressed as a function of the compound's charge *q*, its equivalent hydrodynamic radius $R_{e}$ and the buffer viscosity η [29]:

(5) $$\mu^{\infty }=\frac{q}{\gamma_{h}}=\frac{q}{6\pi \eta R_{e}}$$

As a consequence, the product $\eta \cdot \mu^{\infty }$ is only dependent on the compound's properties and is, namely, independent of the temperature.

For very low ionic strength (up to 1 mM), the actual mobility can be modeled as proportional to the ionic mobility [30], so that the product ημ is also independent of the temperature. Even though the ionic strength used throughout this work (10 mM) is too strong for such a simple model, we hypothesized that the product ημ would approximately be independent of the temperature in our conditions. This was experimentally verified during the step‐by‐step procedure and assumed to be true in the subsequent global procedure.

#### 2.2.3 Viscosity correction

The presence of cyclodextrins induces a non‐negligible increase of the buffer viscosity η(θ) [31] with respect to pure water viscosity at a given temperature $\eta_{0}\left(\theta \right)$ [32]. This effect was evaluated from the variation of the electroosmotic flow as a function of the concentration in cyclodextrins [22], as it is inversely proportional to the buffer viscosity [33].

The electroosmotic flow time *t*_eof_, identified by the elution of the neutral marker, was measured as a function of the concentrations in CD (up to 15 mM) and mCD (up to 100 mM) for temperatures ranging from 15 to 37°C, in pH = 7 and I=10 mM phosphate buffers. The ratio $t_{\mathrm{eof}}/\eta_{0}$ from all experiments was shown to follow a linear evolution with *c*, the concentration in either CD or mCD (see Figure S1A). This variation could be attributed to a linear relationship between the buffer viscosity and *c* :

(6) $$\eta \left(\theta \right)=\left(1+\varepsilon \cdot c\right)\cdot \eta_{0}\left(\theta \right)$$

with $\varepsilon =8.2\pm 0.2\;M^{-1}$, no significant differences could be detected between CD and mCD. The corrected viscosity value from Equation (6) was used in all steps of this work.

The *t*_eof_ values showed large run‐to‐run fluctuations ($R^{2}=0.92$). Moreover, the residual error distribution do not follow a normal distribution, as it is clearly skewed toward larger values (see Figure S1B). This may be explained by the adsorption of compounds on the capillary wall, which essentially tends to lower the electroosmotic flow. The corresponding residual errors were observed to follow a clear χ^2^ random distribution.

#### 2.2.4 pH dependence

Literature values of the p*K*_a_ of the phenolic acids’ carboxylic moiety are reported between 4.37 and 4.43 [34, 35, 36, 37] for C, and 2.92 for R [37, 38] at 25°C and 100 mM ionic strength. Alternative computed values can be found: 4.50 for C [39] and 2.78 for R [40] at 25°C. A higher “estimated” value equal to 3.57 can also be found for R [41], but it is likely inconsistent with the other values and shall be ignored.

The p*K*_a_ values of the phenolic groups are all reported well over 8 [37, 40]. Both R and C are thus monoanions at pH 7, with the full deprotonation of the carboxylic moiety and full protonation of the phenolic group. As a consequence, Equations ((3), (4)) lead to

(7) $$\mu_{X,7}=\frac{\mu_{X}+K_{X^{-}}\cdot c\cdot \mu_{X^{-}L}}{1+K_{X^{-}}\cdot c}$$

with μ_X, 7_, the electrophoretic mobility, measured at pH 7.

At pH < 7, mixtures of protonated and deprotonated compounds exist, so that Equation (3) leads to

(8) $$\mu =\frac{\mu_{X}+K_{X^{-}}\cdot c\cdot \mu_{X^{-}L}}{1+10^{pK_{a}-\mathrm{pH}}+\left(K_{\mathrm{XH}}\cdot 10^{pK_{a}-\mathrm{pH}}+K_{X^{-}}\right)\cdot c}$$

For a given concentration *c*, this expression can be simplified by introducing μ_X, 7_ from Equation (7):

(9) $$\begin{array}{c} \mu =\frac{\mu_{X,7}}{1+10^{pK_{a,\mathrm{app}}-\mathrm{pH}}} \end{array}$$

(10) $$\mathrm{with}\;pK_{a,\mathrm{app}}=pK_{a}+\log\frac{1+K_{\mathrm{XH}}c}{1+K_{X^{-}}c}$$

Equation (9) can be used to determine the apparent variation $\Delta pK_{a}=pK_{a,\mathrm{app}}\left(c\right)-pK_{a}$. This value can in turn be used for the determination of *K*_XH_ from $K_{X^{-}}$ on the basis of Equation (10).

### 2.3 Thermodynamic parameters

In order to take into account the influence of temperature on the equilibrium constants, the p*K* values were linearized as

(11) $$pK=pK_{0}+\lambda \cdot \delta T$$

for low variations $\delta T=T-T_{0}$ of the temperature *T* around $T_{0}=299\;K$ (i.e., $\theta_{0}=26^{\circ }C$), the median temperature of the studied temperature range is ($\theta \in {\left[15-37\right]}^{\circ }C$). The thermodynamic parameters can then be evaluated by

(12) $$\begin{array}{ccc} \Delta_{r}G^{0} & = & \Delta_{r}H^{0}-T\cdot \Delta_{r}S^{0}\\ & = & -RT\ln K=2.303\cdot RT\cdot pK\\ pK & = & \frac{\Delta_{r}H^{0}}{2.303\cdot R\left(T_{0}+\delta T\right)}-\frac{\Delta_{r}S^{0}}{2.303\cdot R}\\ & \approx & \frac{\Delta_{r}H^{0}}{2.303\cdot RT_{0}}\cdot \left(1-\frac{\delta T}{T_{0}}\right)-\frac{\Delta_{r}S^{0}}{2.303\cdot R}\\ pK & \approx & \frac{\Delta_{r}H^{0}-T_{0}\Delta_{r}S^{0}}{2.303\cdot RT_{0}}-\frac{\Delta_{r}H^{0}}{2.303\cdot RT_{0}^{2}}\delta T \end{array}$$

By the association of Equation (11) with Equation (12), we obtain

(13) $$\begin{array}{ccc} \lambda & = & -\frac{\Delta_{r}H^{0}}{2.303\cdot RT_{0}^{2}}\\ pK_{0} & = & \frac{\Delta_{r}H^{0}-T_{0}\Delta_{r}S^{0}}{2.303\cdot RT_{0}} \end{array}$$

This implies

(14) $$\begin{array}{ccc} \Delta_{r}H^{0} & = & -2.303\cdot \lambda \cdot RT_{0}^{2}\\ \Delta_{r}S^{0} & = & -2.303\cdot R\cdot \left(pK_{0}+\lambda \cdot T_{0}\right) \end{array}$$

Both $\Delta_{r}H^{0}$ and $\Delta_{r}S^{0}$ values can, thus, be evaluated from the linearization of the p*K* values with temperature from Equation (11).

It can be noted that an alternative approach would have been to directly model the equilibrium constants, without linearization, as

(15) $$K=e^{\frac{\Delta_{r}S^{0}}{R}}\cdot e^{-\frac{\Delta_{r}H^{0}}{\mathrm{RT}}}$$

so that both $\Delta_{r}H^{0}$ and $\Delta_{r}S^{0}$ values would be directly fitted. However, this model could not be fitted properly. This is linked to the high difficulty to determine accurately the value of $\Delta_{r}S^{0}$: *T* values being essentially gathered in a short [288K,310K] range, any fit would imply that small variations of $\Delta_{r}H^{0}$ would lead to large variations of $\Delta_{r}S^{0}$.

## 3 MATERIALS AND METHODS

### 3.1 Apparatus

All electrophoresis experiments were performed with a Beckman Coulter P/ACE MDQ CE system with UV diode array detection and 32 Karat software (Beckman Coulter, Pasadena, CA, USA). Fused silica capillaries with 50 µm i.d. and 375 µm o.d. were purchased from Agilent (Agilent Technologies, Palo Alto, CA, USA). pH measurements were performed with a pH meter 827 pH lab (Metrohm AG, CH‐9101 Herisau, Switzerland).

### 3.2 Computer software

All the computations were performed using a python [42, 43] environment and were, namely, based on the scipy [44] and numpy [45] libraries for the statistical analysis. Standard nonlinear least‐square methods [46] from scipy were used for the curve fitting procedures. Full data and python script are available in the Supporting Information.

### 3.3 Chemicals

Phosphate (**A** and **G**) and acetate (**B** to **F**) buffer solutions were prepared in Milli‐*Q* water, for pH ranging from 2.9 to 7, adjusted at the same ionic strength of 10 mM (with an addition of NaCl when necessary, see Table S1 for details). The pH of each buffer solution was recorded from 15 to 37°C, and the precise value was used accordingly. Buffers **A**–**F** were prepared with either no cyclodextrin, 15 mM CD or 15 mM mCD. Buffer **G** was prepared with a concentration of up to 15 mM CD and up to 100 mM mCD.

Stock solutions of R and C, both in final concentration equal to 5 mM were prepared and preserved from light at 4°C. These solutions also contained 1 mM of sodium thiosulfate for protection against oxidation. The analyzed samples were prepared daily from these stock solutions to obtain a mixture of rosmarinic acid (0.05 mM), caffeic acid (0.2 mM) and phenol (1 mM).

### 3.4 Capillary conditioning

Prior to first use, capillary was conditioned by successive flushings at 20 psi with water (2 min), 0.1 M NaOH (10 min), 1.0 M NaOH (5 min), water (2 min) and finished with a working buffer (15 min). After every three consecutive runs, the capillary was rinsed with water (2 min), 0.1 M NaOH (5 min), water (2 min) and working buffer (5 min) in order to avoid excessive adsorption of phenolic compounds on the capillary wall. Before each sample run, capillary was flushed with 0.1 M NaOH (1 min) and the working buffer (5 min); after each analysis, the capillary was rinsed with the working buffer (2 min). The sample was injected in hydrodynamic mode at 0.5 psi for 3 s. The analyses were performed under an applied positive voltage of 10 kV with **G** buffer and 20 kV with **A–F** buffers, respectively.

### 3.5 ACE analysis

A sample containing rosmarinic acid (R), caffeic acid (C) and phenol as a neutral marker was analyzed by CE. The electrophoretic mobility μ of R and C in each condition could be measured by

(16) $$\mu =\frac{L_{d}\cdot L_{t}}{U}\left(\frac{1}{t_{X}}-\frac{1}{t_{\mathrm{eof}}}\right)$$

with *t*_eof_, the elution time of the neutral marker; *t*_X_, the elution time of the R or C compound; *U*, the applied positive voltage; L_*t*_, the total length of the capillary (60 cm) and L_*d*_, the length from the capillary input to the detector (50 cm). Three repeats were performed for each set of conditions. As all the analyzed compounds are either anionic or neutral, all measured electrophoretic mobilities are negative. For the sake of simplicity, only the absolute numerical values of the electrophoretic mobilities are given throughout this work to avoid confusion.

A fixed low ionic strength was used for all buffers, as well as a low voltage, in order to minimize the dissipated electric power. Voltages were lowered from 30 to either 20 or 10 kV, so that the dissipated power was kept in the $0.04-0.4\;W\;m^{-1}$ range. Excessive capillary heating by the Joule effect could, thus, be excluded [47], so that the setpoint temperature could be trusted as the actual buffer temperature. Moreover, the variations of actual mobilities due to dielectric friction modifications could be neglected thanks to the constant ionic strength.

The compounds were identified by their UV–visible spectra; at pH 7, both R and C compounds have a first absorbance band at 325 nm, and a second band at 287 nm for R and at 280 nm for C, respectively. A neutral marker enabled the precise determination of the electroosmotic flow; phenol was chosen for its good UV absorption while remaining fully protonated on the whole studied pH scale. It is also nonvolatile, so that its concentration remains stable after a large number of analyses of a given sample. Moreover, migrating far from the charged R and C compounds, it will not compete with them for the binding with CD. A good separation of R and C was observed in most experiments. Full UV–visible spectra were recorded by a diode‐array detector; the UV absorbance detection was specifically monitored at 211, 290 and 325 nm for the assessment of the electrophoretic times.

**FIGURE 2:**
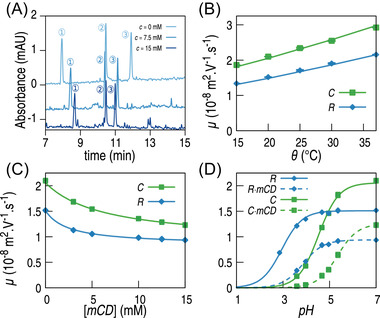
Separation of rosmarinic and caffeic acids by CE in phosphate buffer, pH = 7, I=10 mM, θ=20°C and [CD] = 0, 7.5 or 15 mM; detection at λ=290 nm; 1: Electroosmotic flow, 2: R, 3: C (A); variation of electrophoretic mobilities as a function of temperature, for pH = 7 and c = 0 M (B); of [mCD], for pH = 7 and θ=20°C (C) and of pH, for θ=20°C and [mCD] = 0 or 15 mM (D). Points correspond to experimental measurements, and the lines to curve fitting results

Typical separations are shown in Figure 2A. It can, namely, be seen that both R and C get closer to the neutral marker as the cyclodextrin concentration increases: more anionic R and C compounds are complexed, their apparent electrophoretic mobility is, thus, decreased in absolute value.

## 4 RESULTS AND DISCUSSION

### 4.1 Data analysis

#### 4.1.1 Step‐by‐step curve fitting process

The gathered μ values were first processed by a traditional step‐by‐step procedure for the assessment of the individual parameters:

Step 1: Determination of R and C mobilities.

Electrophoretic mobilities of both R and C were measured at pH 7, in the absence of both CD and mCD, for all temperatures, with fully anionic R^−^ and C^−^. The dependency of $\mu_{X^{-}}$ on temperature could be observed (see Figure 2B and first part of Table S3); the product $\eta \mu_{X^{-}}$ is indeed temperature‐independent and estimated to $\left(1.510\pm 0.041\right)\cdot 10^{-11}\;N\;V^{-1}$ for R^−^ and $\left(2.058\pm 0.017\right)\cdot 10^{-11}\;N\;V^{-1}$ for C^−^, respectively. In the following studies, the product ημ was, thus, systematically used as the parameter to be fitted, instead of directly fitting μ, so that all observed temperature effects can be solely attributed to the variations of the thermodynamic constants.

Step 2: Study of the binding process at pH 7.

At pH 7, R and C are fully deprotonated; Equation (7) has been used for fitting the variation of ημ as a function of c, leading to

(17) $$\eta \mu =\frac{\eta \mu_{X}+K_{X^{-}}\cdot c\cdot \eta \mu_{X^{-}L}}{1+K_{X^{-}}\cdot c}$$

Using the value determined in step 1 for $\eta \mu_{X^{-}}$, parameters $K_{X^{-}}$ and $\eta \mu_{X^{-}L}$ could be determined for the complexes $R^{-}\cdot \mathrm{CD}$, $R^{-}\cdot \mathrm{mCD}$, $C^{-}\cdot \mathrm{CD}$ and $C^{-}\cdot \mathrm{mCD}$ (see second part of Table S3). An example of curve fitting is given in Figure 2C.

**FIGURE 3:**
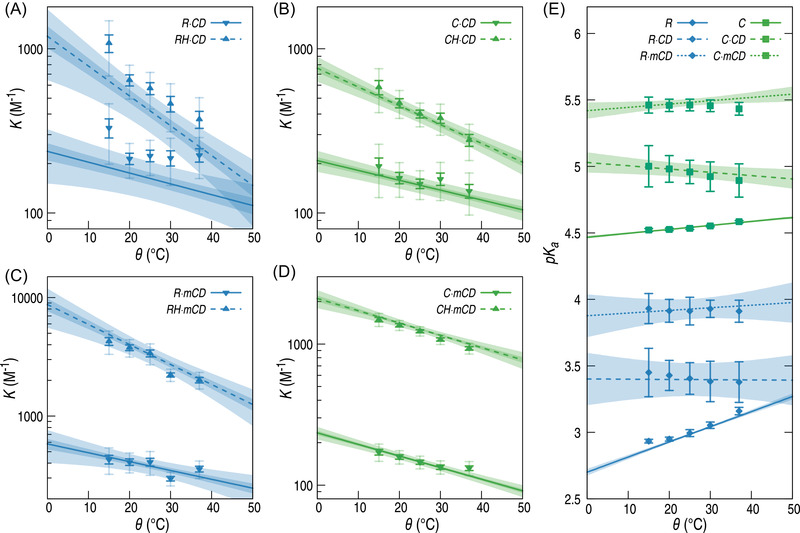
Variation of binding constants $K_{X^{-}}$ (solid lines and down triangles), *K*_XH_ (dotted lines and up triangles) (A–D) and p*K*_a_ (E). Data from step‐by‐step fits are given with triangles and error bars; data from the global fit are given as continuous or dotted line with 1σ (dark) and 3σ (light) error bands (A–D) or a 1σ error band (E).

Once again, it can be observed that the $\eta \mu_{X^{-}L}$ values are temperature‐independent (see last column of Table S3). A general tendency for the binding constants $K_{X^{-}}$ to decrease at higher temperature can be observed (see Figure 3A–D), indicating that the binding reaction is in all cases exothermic.

Step 3: Study of the complexation process as a function of pH

When maintaining a constant concentration in cyclodextrins, the variation of ημ as a function of pH can be expressed using Equation (9):

(18) $$\eta \mu =\frac{\eta \mu_{X,7}}{1+10^{pK_{a,\mathrm{app}}-\mathrm{pH}}}$$

Using the values of $\eta \mu_{X,7}$ as determined from the results of step 2, parameter p*K*_a, app_ could be determined for both R and C, in the absence of cyclodextrins, with 15‐mM CD or with 15 mM mCD (see the last part of Table S3). An example of curve fitting is given in Figure 2D.

In the absence of CD or mCD, $pK_{a,\mathrm{app}}=pK_{a}$ for R and C. The *K*_XH_ binding constants can be obtained from Equation (10), using the $\Delta pK_{a}$ obtained in this step and the $K_{X^{-}}$ values obtained in step 2. In all cases, *K*_XH_ values appear larger than $K_{X^{-}}$, by up to a factor of 10.

All the binding constants, p*K*_a_ and electrophoretic mobilities can, thus, be determined from this three‐step curve fitting for all compounds and temperatures. They are summed up in Table S2 (**S** entries).

#### 4.1.2 Global curve fitting

The second approach consists in performing a single‐step fit from the whole set of data, on the basis of Equations ((8), (11)) that lead to

(19) $$\begin{array}{ccc} \eta \mu & = & \frac{\eta \mu_{X}+f\left(\left[\text{CD}\right]\right)+f\left(\left[\text{mCD}\right]\right)}{1+10^{pK_{a}-\text{pH}}+g\left(\left[\text{CD}\right]\right)+g\left(\left[\text{mCD}\right]\right)}\\ \text{with:}\;f\left(\left[\text{CD}\right]\right) & = & K_{X^{-}\cdot \text{CD}}\cdot \left[\text{CD}\right]\cdot \eta \mu_{\text{XCD}}\\ f\left(\left[\text{mCD}\right]\right) & = & K_{X^{-}\cdot \text{mCD}}\cdot \left[\text{mCD}\right]\cdot \eta \mu_{\text{XmCD}}\\ g\left(\left[\text{CD}\right]\right) & = & \left(K_{\text{XH}\cdot \text{CD}}\cdot 10^{pK_{a}-\text{pH}}+K_{X^{-}\cdot \text{CD}}\right)\cdot \left[\text{CD}\right]\\ g\left(\left[\text{mCD}\right]\right) & = & \left(\;K_{\text{XH}\cdot \text{mCD}}\;\cdot \;10^{pK_{a}-\text{pH}}\;+\;K_{X^{-}\cdot \text{mCD}}\;\right)\cdot \left[\text{mCD}\right]\\ K_{X^{-}\cdot \text{CD}} & = & 10^{-pK_{X^{-}\cdot \text{CD},0}-\lambda_{X^{-}\cdot \text{CD}}\left(\theta -\theta_{0}\right)}\\ K_{X^{-}\cdot \text{mCD}} & = & 10^{-pK_{X^{-}\cdot \text{mCD},0}-\lambda_{X^{-}\cdot \text{mCD}}\left(\theta -\theta_{0}\right)}\\ K_{\text{XH}\cdot \text{CD}} & = & 10^{-pK_{\text{XH}\cdot \text{CD},0}-\lambda_{\text{XH}\cdot \text{CD}}\left(\theta -\theta_{0}\right)}\\ K_{\text{XH}\cdot \text{mCD}} & = & 10^{-pK_{\text{XH}\cdot \text{mCD},0}-\lambda_{\text{XH}\cdot \text{mCD}}\left(\theta -\theta_{0}\right)}\\ pK_{a} & = & pK_{a,X,0}+\lambda_{a,X}\left(\theta -\theta_{0}\right) \end{array}$$

The global fit was performed as a function of the temperature, pH, [CD] and [mCD] for the determination of all other parameters (acidity and binding constants at the median temperature θ_0_, with the λ parameter expressing their temperature dependence). Reasonable initial values for the fitting process were seeded from the results of the step‐by‐step procedure. All the results are given in Table 1. The full set of binding constants, p*K*_a_ and electrophoretic mobilities can then be determined as a function of temperature (see Table S2, **G** entries).

**TABLE 1:** Binding and acidity constants as determined by global nonlinear curve fitting

	C	R	C*·*CD	C*·*mCD	R*·*CD	R·mCD
ημ	2.056 ± 0.003	1.486 ± 0.003	1.13 ± 0.01	0.954 ± 0.006	0.99 ± 0.02	0.856 ± 0.006
p*K*_a, 0_	4.544 ± 0.003	2.997 ± 0.008	–	–	–	–
λ_a_	0.0030 ± 0.0004	0.0114 ± 0.0009	–	–	–	–
p*K*_0_	–	–	−2.16 ± 0.02	−2.157 ± 0.008	−2.20 ± 0.04	−2.57 ± 0.02
λ	–	–	0.0060 ± 0.0006	0.0082 ± 0.0005	0.007 ± 0.001	0.008 ± 0.002
$p{K^{\prime }}_{0}$	–	–	−2.58 ± 0.02	−3.097 ± 0.008	−2.60 ± 0.04	−3.50 ± 0.02
$\lambda^{\prime }$	–	–	0.0114 ± 0.0008	0.0087 ± 0.0008	0.018 ± 0.002	0.017 ± 0.002

*Note*: The indicated errors correspond to the standard deviation issued from the optimization covariance matrix. Units: ημ in $10^{-11}\;N\;V^{-1}$; λ in K^−1^

#### 4.1.3 Residual error statistics

Both fitting procedures give similar values for the binding constants, p*K*_a_ values and electrophoretic mobilities (see Figure 3). However, in most cases, the global curve fitting procedure provides a better precision (characterized by a decrease of the standard deviation) in the determination of the parameters while enforcing at the same time their coherent evolution with temperature. The largest discrepancies are observed for the binding constant between R and CD, the corresponding values being only consistent within an error of 3σ, whereas other constants are consistent within an error of 1σ (see Figure 3A).

The two fitting procedures—either step‐by‐step or global—essentially differ by the degrees of freedom left for the determination of the parameters. The step‐by‐step procedure implies a total of 52 independent fits, for the determination of 72 different parameters. In contrast, the global fit implies only 2 independent fits for the determination of 26 different parameters.

**FIGURE 4:**
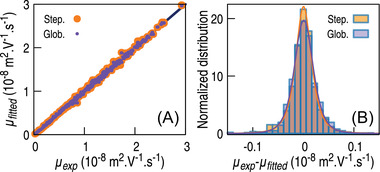
Global (purple) and step‐by‐step (orange) fit statistic: fitted experimental mobilities correlation (A) and residual error distribution (B)

The statistics of the residual error between experimental and fitted values (see Figure 4 and Table S4) show that despite the drastic reduction in the number of parameters, the global fit reproduces the experimental data in a very similar fashion as the step‐by‐step approach. In both cases, a very good correlation is obtained between experimental and fitted data ($R^{2}=0.998$), and the standard deviation of the $\mu_{\exp}-\mu_{\mathrm{fitted}}$ residuals represents about 2.5% of the mean value of all mobilities.

The residuals deviate from a normal distribution with a small—but significant—excess of large errors. The skewness is low, implying a symmetrically distributed residual error, but the positive kurtosis implies the presence of a long tail in the error distribution. The residual distribution was fitted with both a normal distribution and a *t*‐distribution. A KS‐test was then performed for assessing the goodness of each fit; the hypothesis of normal distribution was then rejected (p=0.001), implying that the residuals rather follow a Student's *t*‐distribution (p=0.6). The corresponding ν “degree of freedom” parameter of the fitted *t*‐distribution is noticeably different between the step‐by‐step (2.73) and the global (4.30) procedure, implying that the latter one is closer to a normal distribution than the former. The global curve fitting is, thus, marginally better than the step‐by‐step procedure; it diminishes the largest errors but does not eliminate them. This long tail in the residual error distribution is, however, unevenly distributed, as most of the errors stems from R (the Student's ν parameter are 4.12 and 8.67 for, respectively, Rand C with the global procedure). These fluctuations may be linked to a larger propensity of rosmarinic acid for adsorption to the surface capillary, decreasing the reproducibility of its electrophoretic mobility.

### 4.2 Physicochemical parameters

#### 4.2.1 Electrophoretic mobilities

All the electrophoretic mobilities obtained by the step‐by‐step procedure are, as initially hypothesized, inversely proportional to the buffer viscosity (see Figure S2). Moreover, they are globally consistent with what can be expected from their relative size. Indeed, the hydrodynamic radius of the compounds can be evaluated from the μ values, by following Equation (5) (see Figure S3 and Table S5). According to these values, caffeic acid ($R_{e}=3.8\;Å$) is smaller than rosmarinic acid ($R_{e}=5.0\;Å$). Their complexation leads to a size increase, with the CD complexes having a radius smaller by about 1 Å than the mCD complexes (7.1 and 6.4 Å for $R^{-}\cdot \mathrm{CD}$ and $C^{-}\cdot \mathrm{CD}$, 8.1 and 7.4 Å for $R^{-}\cdot \mathrm{mCD}$ and $C^{-}\cdot \mathrm{mCD}$). The radii of the $X^{-}\cdot \mathrm{CD}$ complexes are close to but significantly smaller than the hydrodynamic radius of CD as reported in the literature (7.7 Å) [48]. This small discrepancy probably originates from an inaccurate ionic strength correction; a more precise evaluation of the hydrodynamic radius would require a systematic study as a function of the ionic strength [47, 49].

#### 4.2.2 Enthalpic and entropic contributions

In many cases, the step‐by‐step curve fitting process leads to important noise in the final values of the binding constants, in terms of their evolution with temperature. This is especially visible for the R − CD binding (see Figure 3A). In order to correctly evaluate the thermodynamic parameters corresponding to these constants, it was necessary to rely solely on the values obtained from the global procedure, where the temperature dependence was enforced as a constraint in the model. This leads to a good evaluation of the $\Delta_{r}H^{0}$ values for binding reactions, whereas significant values for the $\Delta_{r}S^{0}$ were only observed in specific cases (see Table 2).

**TABLE 2:** Thermodynamic parameters as determined by global nonlinear curve fitting ($\Delta_{r}H^{0}$ and $\Delta_{r}G^{0}$ values in $\mathrm{kJ}\;{\mathrm{mol}}^{-1}$, $\Delta_{r}S^{0}$ values in $J\;K^{-1}\;{\mathrm{mol}}^{-1}$)

		$pK(T_{0})$	$\Delta_{r}G^{0}\left(T_{0}\right)$	$\Delta_{r}H^{0}$	$\Delta_{r}S^{0}$	$x_{S}$	$x_{H}$	$r_{SH}$
$K_{X^{-}}$	$R^{-}\cdot \mathrm{CD}$	−2.20 ± 0.04	−12.6 ± 0.2	−11 ± 2	4 ± 8	0.10	0.90	0.11
	$R^{-}\cdot \mathrm{mCD}$	−2.57 ± 0.02	−14.7 ± 0.1	−13 ± 3	6 ± 8	0.13	0.87	0.15
	$C^{-}\cdot \mathrm{CD}$	−2.16 ± 0.02	−12.39 ± 0.09	−10.3 ± 0.9	7 ± 3	0.17	0.83	0.20
	$C^{-}\cdot \mathrm{mCD}$	−2.157 ± 0.008	−12.35 ± 0.05	−14.1 ± 0.8	−6 ± 3	−0.14	1.14	−0.12
*K*_XH_	RH · CD	−2.60 ± 0.04	−14.9 ± 0.2	−31 ± 4	−50 ± 10	−1.09	2.09	−0.52
	RH · mCD	−3.50 ± 0.02	−20.1 ± 0.1	−29 ± 3	−30 ± 10	−0.44	1.44	−0.31
	CH · CD	−2.58 ± 0.02	−14.80 ± 0.09	−20 ± 1	−16 ± 4	−0.32	1.32	−0.24
	CH · mCD	−3.097 ± 0.008	−17.73 ± 0.05	−15 ± 1	9 ± 4	0.16	0.84	0.19
p*K*_a_	R	2.997 ± 0.008	17.16 ± 0.05	−20 ± 2	−122 ± 5	2.14	−1.14	−1.88
	C	4.545 ± 0.003	26.02 ± 0.02	−5.1 ± 0.6	−104 ± 2	1.20	−0.20	−6.00
	R · CD	3.4 ± 0.1	19.5 ± 0.7	0 ± 5	−60 ± 20	0.98	0.02	49.0
	C · CD	4.97 ± 0.05	28.4 ± 0.3	4 ± 2	−81 ± 6	0.85	0.15	5.67
	R · mCD	3.93 ± 0.08	22.5 ± 0.4	−3 ± 4	−90 ± 10	1.15	−0.15	−7.67
	C · mCD	5.48 ± 0.03	31.4 ± 0.2	−4 ± 2	−119 ± 6	1.14	−0.14	−8.14

*Note*: $\Delta_{r}H^{0}$ and $\Delta_{r}S^{0}$ are supposed to be independent of the temperature; p*K* and $\Delta_{r}G^{0}$ are calculated at $T_{0}=299$ K; $x_{S}=\frac{-T_{0}\Delta_{r}S^{0}}{\Delta_{r}G^{0}}$ and $x_{H}=\frac{\Delta_{r}H^{0}}{\Delta_{r}G^{0}}$ are the fraction of, respectively, entropic and enthalpic contribution to $\Delta_{r}G^{0}$ at *T*_0_. $r_{SH}=\frac{-T_{0}\Delta_{r}S^{0}}{\Delta_{r}H^{0}}$ is the ratio between entropic and enthalpic contributions.

All binding reactions are exothermic, with negative values for $\Delta_{r}H^{0}$ ranging from −10 to $-30\;\mathrm{kJ}\;{\mathrm{mol}}^{-1}$. The entropy of reaction is only significant for the formation of the RH · CD, RH · mCD and CH · CD complexes, where negative values imply an unfavorable entropic contribution. These complexes remain very stable due to a large negative enthalpy. For the other complexes, the binding reactions are essentially enthalpy‐driven and are characterized by very low $\Delta_{r}S^{0}$ (less than ca. $10\;J\;K^{-1}\;{\mathrm{mol}}^{-1}$), the determined values of which are of the same order of magnitude as their standard errors.

The acid–base reactions are essentially governed by the large negative entropy—implying that the protonation of the carboxylate moiety is favored—with less than 20% enthalpic contribution. In the case of rosmarinic acid, a strong negative enthalpy of proton transfer counteracts a strong entropic contribution, which favors the deprotonation and leads to a low p*K*_a_ value; it may reflect the favorable inductive effect of the adjacent C–O bond, resulting in a more labile proton.

The p*K*_a_ of the X · L complexes results from the combination of the binding and acid–base reactions. Binding the protonated forms is more exothermic than binding the deprotonated form. The exothermicity of the deprotonation of free R or C is, thus, counteracted by the loss of binding exothermicity when the phenolic acid is deprotonated. This leads to unusually low reaction enthalpies for the deprotonation of the complexes. The deprotonation of the C · CD complex is even endothermic.

#### 4.2.3 Binding efficiency

The binding constants were measured at an ionic strength of 10 mM. $K_{X^{-}}$ shall depend on the ionic strength, due to the difference between the anionic reactant and product radius. Having evaluated the different hydrodynamic radius of all the charged compounds, it is thus possible to evaluate the actual thermodynamic value for $K_{X^{-}}$ (see Supporting Information for details). A correction of about 0.003 unit of p*K*—less than 1% correction on the $K_{X^{-}}$ values—was obtained. This corresponds to about a tenth of the standard error measured for these constants. As a consequence, the effect of ionic strength can be neglected.

Binding reactions are highly sensitive to pH and temperature variations (see Figure S4). The neutral CH and RH possess a much higher affinity for cyclodextrins than their anionic forms. Moreover, the binding is exothermic and, thus, favored at low temperature. As a consequence, efficient encapsulation can be reached at low pH and low temperature. For temperature varying from 0 to 50°C and pH from 1 to 7, the global binding constant can easily vary by one order of magnitude.

As R and C display a similar affinity for CD, it can be proposed that the encapsulation of rosmarinic acid, and its anion into CD mostly involves their common caffeic moiety. This structure is consistent with molecular dynamics simulation studies from the literature[27]. This is no longer true with mCD, which binds R more strongly than C. Thus, the 3,4‐dihydroxyphenylacetic moiety, specific to the rosmarinic acid and its anion, probably participates in the encapsulation into the partially methylated macrocycle. Compared to CH, RH binds mCD by a ca. twice more exothermic reaction, although at an entropic cost possibly reflecting a stiffening of the 3,4‐dihydroxyphenylacetic moiety. Finally, when comparing CD and mCD, the much higher affinity of the latter for RH is likely rooted in the less unfavorable entropy change, which may be interpreted by a stronger stiffening of the 3,4‐dihydroxyphenylacetic moiety, possibly by H‐bonding to CD.

#### 4.2.4 Acidity constants

The p*K*_a_ values of R and C, measured at an ionic strength of 10 mM, can be compared with the literature data, namely, with the values reported by Maegawa et al. [37] at a ionic strength of 100 mM and 25°C. Because of the ionic strength difference, it is necessary to first extrapolate the corresponding values at infinite dilution using the Debye–Hückel equation. That implies a correction factor of about +0.044 from 10 mM ionic strength and +0.11 from 100 mM ionic strength, respectively [17]. Thus, the corrected p*K*_a_ values at I=0 measured in this work are, for R and C, respectively, 3.029 ± 0.008 and 4.585 ± 0.003 at 25°C. These values are in very good agreement with the corrected values from the literature [37] 3.03 ± 0.14 and 4.54 ± 0.02.

As we systematically observed $K_{\mathrm{XH}}>K_{X^{-}}$, Equation (2) implies that the protonated form XH is more stabilized by complexation than the anionic form X^−^ and is, thus, less acidic. An increase of up to one p*K*_a_ unit is observed.

## 5 CONCLUDING REMARKS

The first approach, which consists in successively determining different parameters in an incremental procedure, is sufficiently precise to give globally consistent results. However, it can lead to large inaccuracies, diminishing the relevance of the final results. More accurate results can be obtained by fitting the full set of experimental data.

Processing all the data with a global curve fitting procedure brings several advantages:

- The step‐by‐step approach leads to multiple independent determinations of parameters that are actually linked to each other. Reducing the number of parameters to describe the full system in a global fitting enforces the coherence of the results.
- At the same time, the drastic reduction of the number of adjustable parameters increases the constraints on the model. This limits the possibility of overfitting, as a too large number of parameters may lead to fit variations that are actually just noise.
- Each data point is only fitted once in the global fitting procedure, whereas some data points are reused in different successive fits in the step‐by‐step procedure. An overrepresentation of specific data points can, thus, be avoided as the same weight is given to all data points.

As a consequence, fully coherent results can be obtained by increasing the constraints on the fitted parameters, that is, by reducing the degrees of freedom while retaining a good statistical description of the experimental data.

However, it is important to keep in mind that a global fitting procedure requires a correctly chosen model. The step‐by‐step fit is still valuable for the validation of each independent hypothesis. It may also be difficult to obtain a good fit convergence from a single fitting procedure with a large number of parameters. It is important to initially seed such a procedure with initial good guesses on the parameters; the results from a step‐by‐step procedure can be used for this purpose. As a consequence, a global fit procedure should be used as an important refinement of the results obtained after a classical step‐by‐step procedure.

With this fitting procedure, all the parameters necessary for assessing the complexation of rosmarinic and caffeic acid with CD and mCD were determined. All binding constants, as well as the p*K*_a_ values for rosmarinic and caffeic acids in both free and bound forms, were obtained in good agreement with the few available values from the literature; they were further extended to a large range of temperature and pH. It could be observed that the binding reaction is very sensitive to these parameters, with an optimal encapsulation achieved at low pH and low temperature. These results are of great interest for the optimization of extraction procedures involving the reversible encapsulation of rosmarinic and caffeic acid—and by extension of other phenolic acids—with cyclodextrins. Moreover, a controlled adsorption/release procedure can be envisioned, by switching from low pH/low temperature conditions to high pH/high temperature conditions.

## CONFLICT OF INTEREST

The authors have declared no conflict of interest.

## Supporting information

Additional information, and as referred within the text are available in a supplementary Supplementary.pdf file

The full data and python scripts used in this work are provided within a single Python‐script.rar archive file.

All curves generated by the fitting process are available in a fits.pdf file.

## Data Availability

The data that support the findings of this study are available in the supplementary material of this article.
