# Supplementary material for: Encapsulation of phenolic acids into cyclodextrins: A global statistical analysis of the effects of pH, temperature and concentrations on binding constants measured by ACE methods
Source: Electrophoresis. 2022 Jul 10;43(23-24):2290–301. doi: 10.1002/elps.202200075 (PMC10083966; doi:10.1002/elps.202200075)
Supplement: Supplementary file 1 — Additional information, and as referred within the text are available in a supplementary Supplementary.pdf file [file ELPS-43-2290-s001.pdf]

Supplementary Information to "Encapsulation of phenolic acids into cyclodextrins: a global statistical analysis of the effects of *pH*, temperature and concentrations on binding constants measured by ACE methods"

Amra Aksamija, Valérie Tomao, Olivier Dangles, Raphaël Plasson*

Avignon University, INRAE, UMR408, 84000, Avignon, France

S1. Chemicals and reagents

Rosmarinic and caffeic acid, methyl-β-cyclodextrin, sodium dihydrogenphosphate dihydrate and disodium hydrogenphosphate heptahydrate were purchased from Sigma-Aldrich, (Steinheim, Germany). β-cyclodextrin was obtained from Panreac Quimica (Barcelona, Spain). Sodium hydroxide and acetic acid (99%) were purchased from Prolabo (Haasrode, Belgium). Phenol was obtained from Carlo Erba (Peypin, France) and sodium acetate trihydrate was purchased from Alfa Aesar (Schiltigheim, France). All used reagents are analytical grade quality.

Standard solutions for capillary conditioning, sodium hydroxide 0.1 M and 1.0 M were supplied by Fluka Biochemika (Steinheim, Germany) while the milliQ water was produced by EASY pure RF compact ultrapure water system (Barnstead, ThermoFicher Scientific, Waltham, MA, USA).

S2. Buffer preparation

All working buffers were prepared in milliQ water, and conserved at 4 °C.

A was prepared from 10 mM NaOH, adjusted with phosphoric acid to desired *p*H (2.84 at 25 °C)

B, C were prepared from 5 mM NaCl and 5 mM NaOH solutions, adjusted to the desired *p*H (3.6 and 3.94 at 25 °C) with acetic acid.

D was prepared from 10 mM sodium acetate trihydrate adjusted with acetic acid (*p*H 4.67 at 25 °C).

E, F were prepared from 10 mM NaOH solutions, adjusted to the desired *p*H (4.92 and 5.35 at 25 °C) with acetic acid.

G was prepared from 2.80 mM sodium dihydrogenphosphate dihydrate and 2.40 mM NaOH disodium hydrogenphosphate heptahydrate.

Buffers **A** to **F** were prepared with either no cyclodextrins, 15 mM *CD*, or 15 mM *mCD*. Buffer **G** was prepared with concentration up to 15 mM of *CD*, and up to 100 mM of *mCD*. The final ionic strength of all these solutions is 10 mM.

S3. Python script

A supplementary `Python-script.rar` file containing the `python` script necessary for performing the curve fitting processes is available. It contains the `datafile-local.py` script, `.txt` files containing instructions to be used by the python script, and `.dat` files containing experimental data. The full data processing will be performed through the command:

```
python datafile-local.py process.txt >
    result-process.txt
```

This command will generate two files containing all the processed data (`ros_caf.xls` and `ros_caf.hdf5`), and a `fits.pdf` file containing graphical representation of the fitting processes. The files `Residuals.html` and `Residuals.pdf` were obtained from the `jupyter` notebooks giving the details of the final statistical analysis of the fitting processes.

S4. Curve fitting parameters

The step-by step procedure implies:

1. the measurement of mean values of R and C mobilities for 5 temperatures, at *p*H 7, and in absence of cyclodextrins; this leads to the first two parameters: $\eta\mu_X$ for both R and C (thus 2 operations for the determination of 2 parameters);
2. 20 independent fits (for the 4 complexes R·CD, C·CD, R·mCD and C·mCD, each performed at five temperatures), each for the determination of 2 parameters $\eta\mu_{XL}$ and K_{X^-}, are performed from the measurements at *p*H 7 with various cyclodextrin concentrations (thus 20 operations for the determination of 40 parameters);

*Corresponding author

Email addresses: amra.aksamija@univ-avignon.fr (Amra Aksamija), valerie.tomao@univ-avignon.fr (Valérie Tomao), olivier.dangles@univ-avignon.fr (Olivier Dangles), raphael.plasson@univ-avignon.fr (Raphaël Plasson), +33.4.90.14.44.41 (Raphaël Plasson)

3. 30 independent fits (for the 6 compounds R, C, R·CD, R·mCD, C·CD and C·mCD at 5 temperatures), each for the determination of one parameter $pK_{a,app}$, are performed from the measurements as a function of pH (so 30 operations for the determination of 30 parameters).

This results in a total of 52 independent operations, for the determination of 72 different parameters.

As a contrast, the global fit implies only 2 independent fits for R and C (noted X below), each for the determination of 13 parameters:

- $pK_{a,X,0}$
- $\lambda_{a,X}$
- $\eta\mu_X$
- $\eta\mu_{XCD}$ and $\eta\mu_{XmCD}$
- $pK_{X^-\cdot CD,0}$ and $pK_{X^-\cdot mCD,0}$
- $\lambda_{X^-\cdot CD}$ and $\lambda_{X^-\cdot mCD}$
- $pK_{XH\cdot CD,0}$ and $pK_{XH\cdot mCD,0}$
- $\lambda_{XH\cdot CD}$ and $\lambda_{XH\cdot mCD}$

Each of this set of parameters is performed by one fit on all data related to either R or C, thus resulting in total to the determination of 26 different parameters.

The full results of the fitting processes are given in a separate `fits.pdf` file:

- Pages 1-3 give the fitting process for the viscosity correction $\frac{t_{eof}}{\eta_0} = f(c)$ (linear regression, non-linear curve fitting process, and experimental/fitted data correlation curve)
- Pages 4-63 give the details of the step 3 fitting process (30 non-linear curve fitting processes, with the corresponding experimental/fitted data correlation curve), i.e. $\mu_{C,app} = f(pH)$ (pp 4-33) and $\mu_{R,app} = f(pH)$ (pp 34-63) for different T, in absence or presence of CD or mCD.
- Pages 64-83 give the details of the step 2 fitting process for R (20 experimental/fitted data correlation curve for each multiparameter non-linear curve fitting process), i.e. $\mu_{R,app} = f([CD])$ (pp 64-68), at $pH = 7$, $\mu_{C,app} = f([CD])$ (pp 69-73), $\mu_{R,app} = f([mCD])$ (pp 74-78), $\mu_{C,app} = f([mCD])$ (pp 79-84), for different T.
- Pages 84-85 give the experimental/fitted data correlation curve for the two global fitting processes: $\mu_{R,app}$ (p 84) and $\mu_{C,app}$ (p 85).

S5. Hydrodynamic radius

In order to evaluate the compound radius from their electrophoretic mobilities, it is necessary to evaluate the ionic mobility from the actual mobility.

This can be performed using the Debye-Hückel-Onsager equation:

$$\mu = \mu^\infty - (A_1 + A_2\mu^\infty)\frac{\sqrt{I}}{1 + Br_i\sqrt{I}} \tag{S1}$$

Numerical values of the parameters can be obtained from the literature[S1, S2]. After this correction performed, the resulting ionic mobility can be used for the evaluation of the radius of the corresponding compound using Eq. (10). The equivalent radius R_e correspond to the radius of an equivalent sphere, that would possess the same frictional coefficient γ_h than the compound. See fig. S3 and table S5.

S6. Influence of ionic strength on binding constants

The thermodynamic binding constant for the compound X with the ligand L can be written as:

$$K^0 = \frac{a_{X\cdot L}}{a_X \cdot a_L} \tag{S2}$$

with

$$a_Y = \gamma_Y \cdot [Y] \tag{S3}$$

$$\log \gamma_Y = -A \cdot z_Y^2 \frac{\sqrt{I}}{1 + B \cdot r_Y \cdot \sqrt{Y}} \tag{S4}$$

$$A = 0.51\ M^{-0.5} \tag{S5}$$

$$B = 3.29\ nm^{-1}.M^{-0.5} \tag{S6}$$

where a_Y is the activity of compound Y, z_Y its charge, r_Y its ionic radius, and I is the solution ionic strength. This thermodynamic binding constant K^0 can be expressed as a function of the apparent binding constant K, written as a function of the concentrations instead of the activities:

$$K^0 = \frac{\gamma_{X\cdot L}[X \cdot L]}{\gamma_X[X] \cdot \gamma_L[L]} \tag{S7}$$

$$= \frac{\gamma_{X\cdot L}}{\gamma_X \cdot \gamma_L} \cdot K \tag{S8}$$

For neutral compounds, $\gamma = 1$. As a consequence, $K_{XH} = K^0_{XH}$ as all the compounds are uncharged, in which case the measured binding constant do not depend on the concentrations. This is not the case for K_{X^-}, that involves two anionic compounds:

$$K^0_{X^-} = \frac{\gamma_{X^-\cdot L}}{\gamma_{X^-}} \cdot K_{X^-} \tag{S9}$$

$$pK^0_{X^-} = pK_{X^-} + A\sqrt{I}\left(\frac{1}{1 + B \cdot r_{X^-\cdot L}\sqrt{I}} - \frac{1}{1 + B \cdot r_{X^-}\sqrt{I}}\right) \tag{S10}$$

The measurements were performed at $I = 10^{-2}$ M, and the different compound radius were evaluated to 0.50 nm, 0.38 nm, 0.71 nm, 0.64 nm, 0.81 nm and 0.75 nm for respectively R^-, C^-, $R^- \cdot CD$, $C^- \cdot CD$,$R^- \cdot mCD$ and $C^- \cdot mCD$. This implies a correction to pK_{X^-} that is equal to 0.0025, 0.0032, 0.0035, 0.0044 for respectively $R^- \cdot CD$, $C^- \cdot CD$,$R^- \cdot mCD$ and $C^- \cdot mCD$ (the corresponding standard error being equal respectively to 0.04, 0.02, 0.02 and 0.08).

References

(S1) Li, D., Fu, S., and Lucy, C. A. (1999). Prediction of electrophoretic mobilities. 3. effect of ionic strength in capillary zone electrophoresis. *Analytical Chemistry*, 71(3):687–699.

(S2) Plasson, R. and Cottet, H. (2005). Determination of homopolypeptide conformational changes by the modeling of electrophoretic mobilities. *Anal Chem*, 77(18):6047–54.

List of Figures

S1 (**A**) Variation of the t_{eof}/η_0 ratio with cyclodextrin concentration c; pale blue dots: individual measurements, blue bars: mean and standard deviation at a given concentration, green line: linear regression applied to all points. (**B**) Residual errors distribution; green line: χ^2 fitting of the residuals distribution. 5
S2 Variation of the electrophoretic mobilities of free and bound R and C as a function of temperature, at pH = 7 (buffer **G**), in absence or presence of CD and mCD at concentration 15 mM. 6
S3 $\eta\mu^{\infty}$ (**A**) and R_e ((**B**)) values as estimated from fits at pH 7 and from the global fit. (**C**): Representation of the equivalent radius R_e; the half-circle radius are proportional to the R_e value of the corresponding compound. . . 7
S4 Variation of the global binding constant as a function of temperature and pH. 8

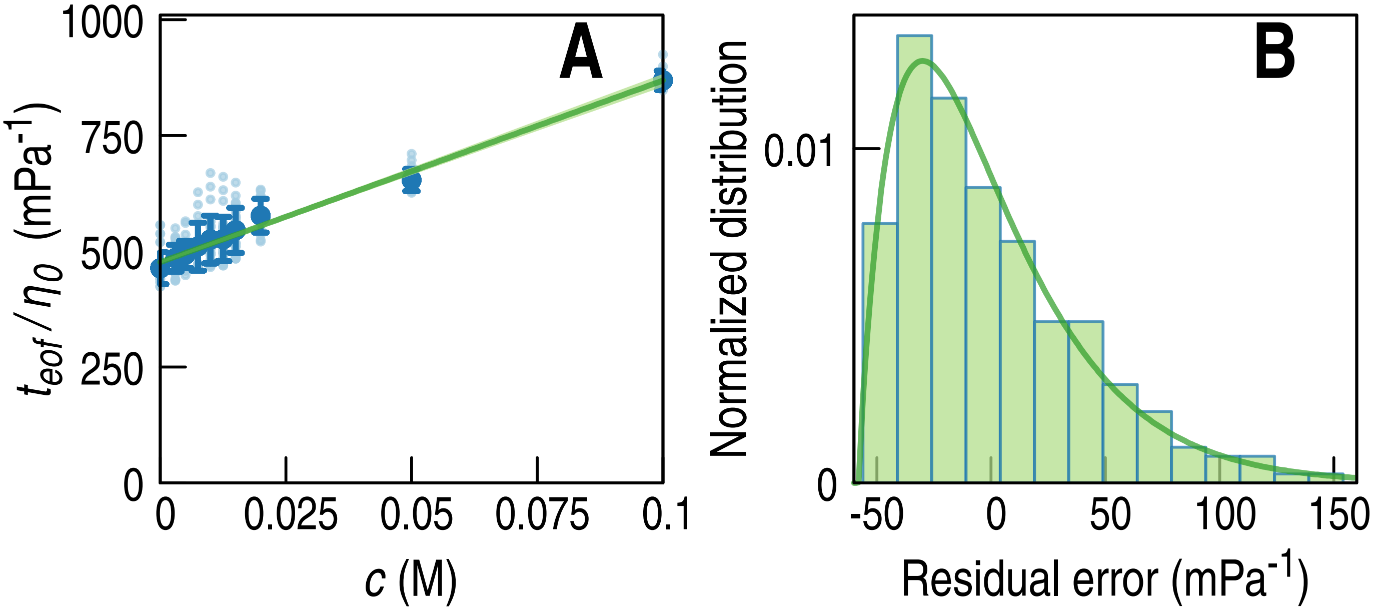

Figure S1: (**A**) Variation of the t_{eof}/η_0 ratio with cyclodextrin concentration c; pale blue dots: individual measurements, blue bars: mean and standard deviation at a given concentration, green line: linear regression applied to all points. (**B**) Residual errors distribution; green line: χ^2 fitting of the residuals distribution.

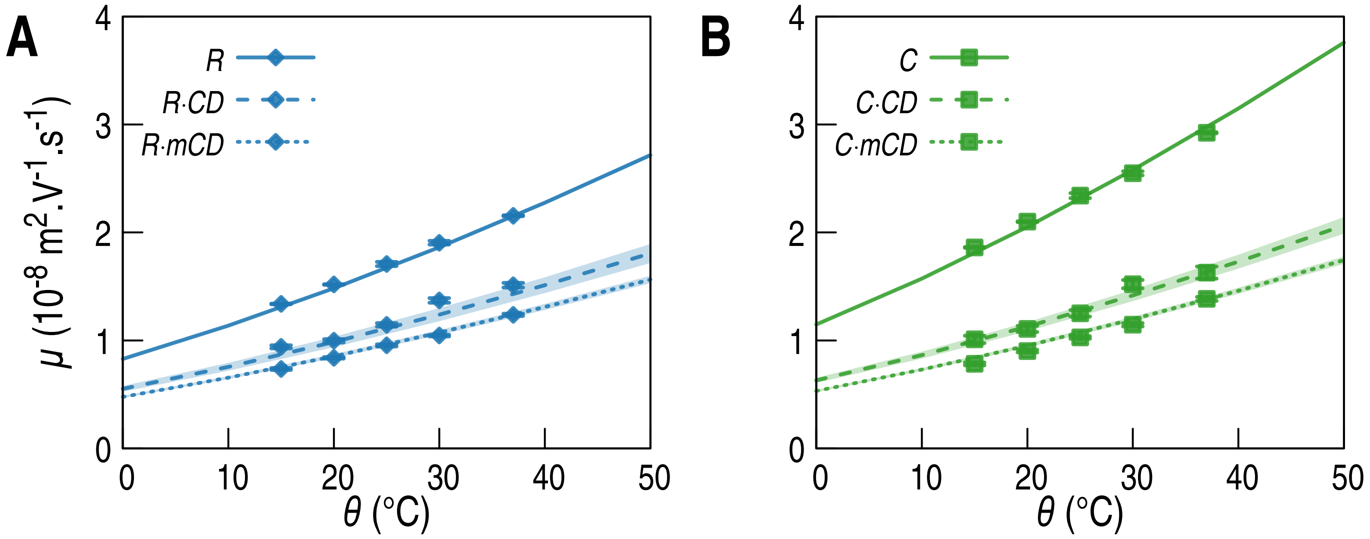

Figure S2: Variation of the electrophoretic mobilities of free and bound R and C as a function of temperature, at pH = 7 (buffer **G**), in absence or presence of CD and mCD at concentration 15 mM.

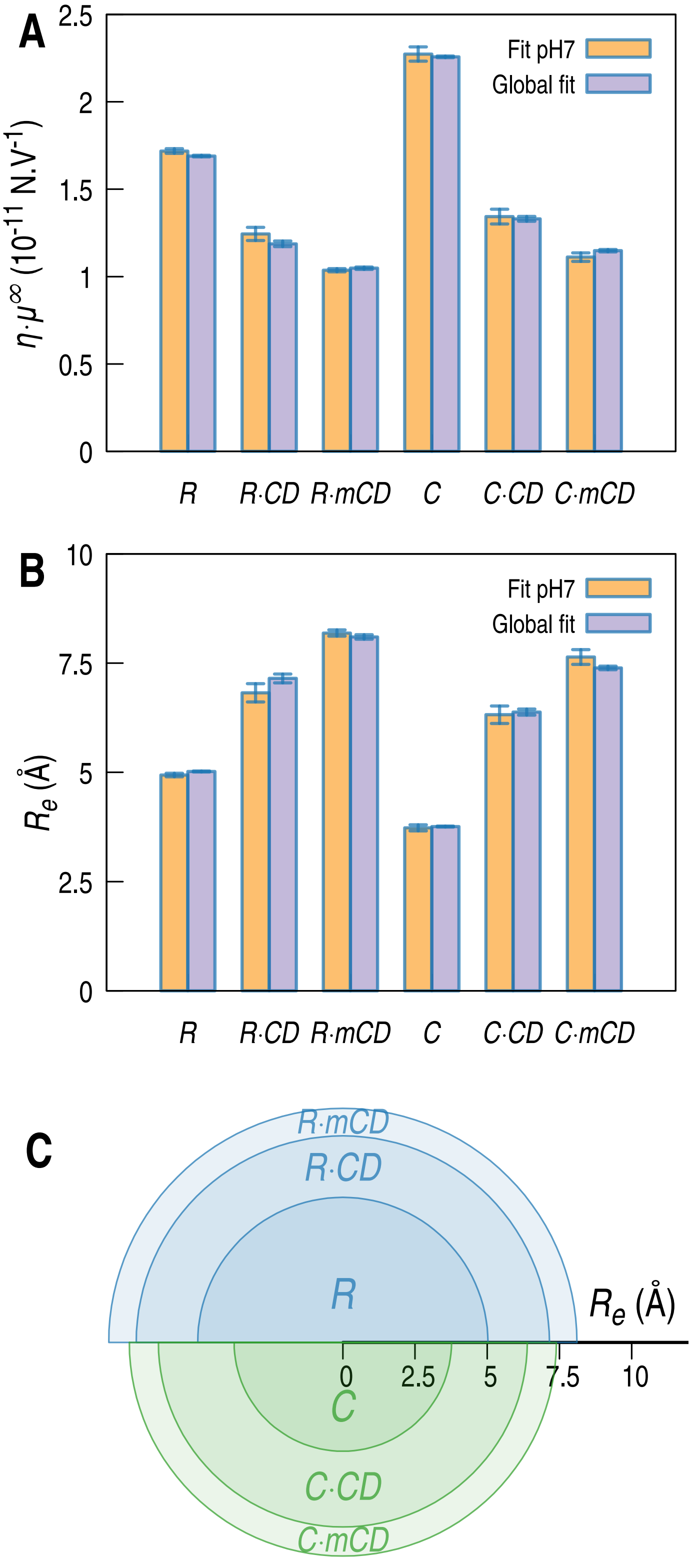

Figure S3: $\eta\mu^{\infty}$ (**A**) and R_e ((**B**)) values as estimated from fits at *p*H 7 and from the global fit. (**C**): Representation of the equivalent radius R_e; the half-circle radius are proportional to the R_e value of the corresponding compound.

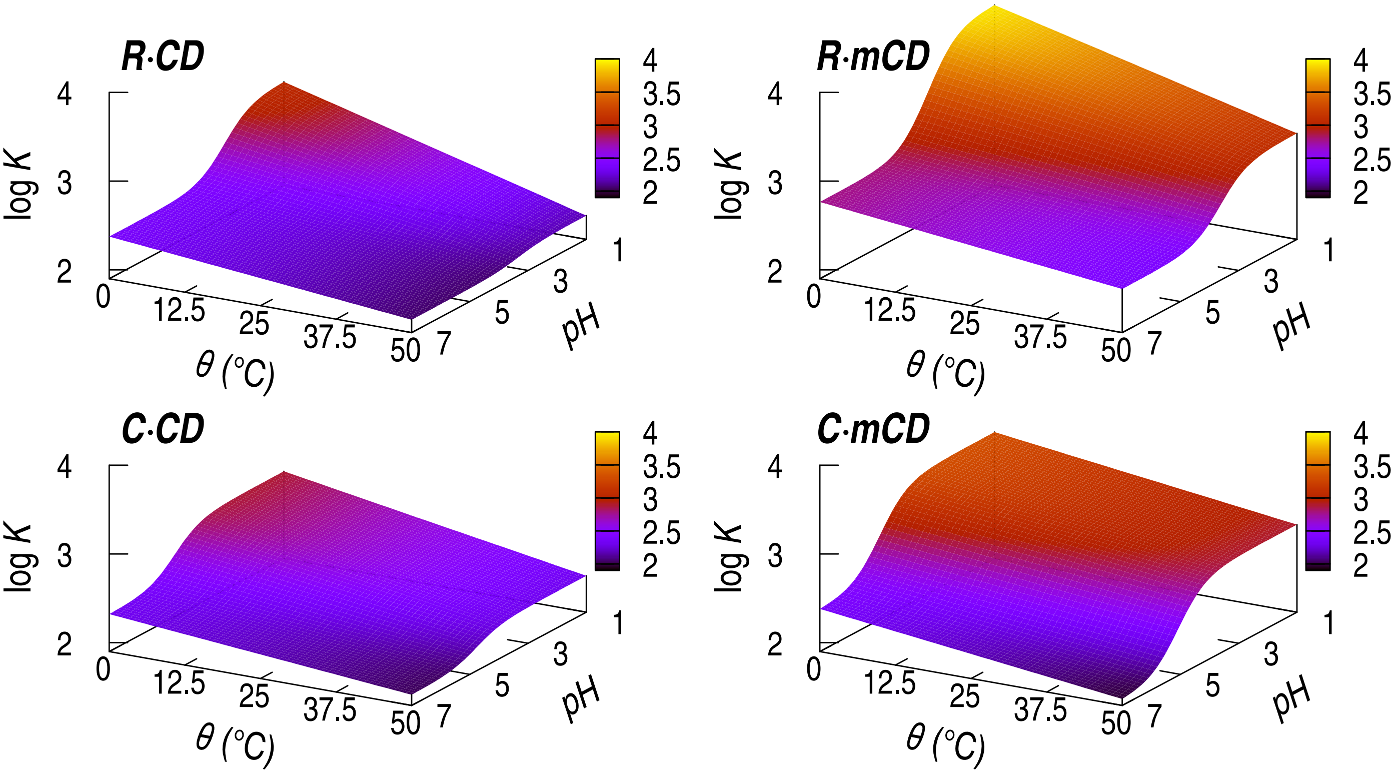

Figure S4: Variation of the global binding constant as a function of temperature and pH.

List of Tables

S1 Viscosity and pH variations of buffers with temperature. The buffers **A-G** were prepared as described in section S2. 10
S2 K_{X^-}, K_{XH}, pK_a and μ of R and C. **S** indicate values obtained from step-by-step fits, **G** from global fit. 11
S3 Fitted values from the step-by-step fitting process. Units: μ_X in 10^{-8} $m^2.V^{-1}.s^{-1}$; $\eta\mu_{XL}$ in 10^{-11} $N.V^{-1}$; K_{X^-} in M^{-1} 12
S4 Residuals statistics. The last column labelled "Ideal" gives the ideal values that would be obtained in the case of residuals with normally distributed noise. Residual statistics: the skewness represent the symmetry residuals around the mean; the kurtosis represent the presence of distribution tails (positive values indicating larger tails than for the normal distribution). All statistics were performed from electrophoretic mobilities in 10^{-8} $m^2.V^{-1}.s^{-1}$ units. 13
S5 $\eta \cdot \mu$ viscosity-mobility product, $\eta \cdot \mu^\infty$ viscosity-mobility product corrected at $I = 0$ (10^{-11} $N.V^{-1}$), and equivalent hydrodynamic radius R_e (Å). 14

Table S1: Viscosity and *p*H variations of buffers with temperature. The buffers **A**-**G** were prepared as described in section S2.

Buffer label	θ (°C)	15	20	25	30	37
**A**		2.82	2.83	2.84	2.85	2.87
**B**		3.63	3.61	3.60	3.60	3.61
**C**		3.93	3.93	3.94	3.95	3.96
**D**	*p*H	4.66	4.67	4.67	4.69	4.70
**E**		4.94	4.93	4.92	4.93	4.94
**F**		5.35	5.35	5.35	5.36	5.37
**G**		7.00	7.00	7.00	7.00	7.00
(water)	η_0 (mPa.s)	1.139	1.002	0.8904	0.7975	0.6915

Table S2: K_{X^-}, K_{XH}, pK_a and μ of R and C. **S** indicate values obtained from step-by-step fits, **G** from global fit.

θ (°C)			15	20	25	30	37
K_{X^-} (M^{-1})	R · *CD*	**S**	330 ± 40	210 ± 20	220 ± 20	220 ± 20	230 ± 20
		**G**	190 ± 20	180 ± 20	160 ± 10	150 ± 10	140 ± 10
	R · *mCD*	**S**	430 ± 40	410 ± 30	410 ± 30	300 ± 10	360 ± 20
		**G**	450 ± 30	410 ± 20	380 ± 20	350 ± 20	310 ± 20
	C · *CD*	**S**	190 ± 20	160 ± 10	150 ± 10	160 ± 10	140 ± 10
		**G**	169 ± 7	160 ± 6	148 ± 5	138 ± 5	125 ± 5
	C · *mCD*	**S**	171 ± 8	160 ± 6	145 ± 5	134 ± 5	132 ± 5
		**G**	177 ± 4	160 ± 3	146 ± 3	133 ± 3	117 ± 3
K_{XH} (M^{-1})	*RH · CD*	**S**	1100 ± 100	640 ± 50	570 ± 50	460 ± 50	370 ± 40
		**G**	640 ± 70	520 ± 50	420 ± 40	340 ± 30	250 ± 30
	RH · mCD	**S**	4300 ± 300	3800 ± 200	3400 ± 200	2220 ± 90	2000 ± 100
		**G**	4900 ± 300	4000 ± 200	3300 ± 200	2700 ± 200	2100 ± 100
	CH · CD	**S**	580 ± 60	470 ± 30	400 ± 20	380 ± 30	280 ± 20
		**G**	510 ± 20	450 ± 20	390 ± 10	350 ± 10	290 ± 10
	CH · mCD	**S**	1490 ± 50	1360 ± 40	1230 ± 30	1080 ± 30	930 ± 30
		**G**	1560 ± 40	1410 ± 30	1280 ± 20	1150 ± 20	1000 ± 30
pK_a	R	**S**	2.93 ± 0.01	2.95 ± 0.02	3.00 ± 0.03	3.05 ± 0.02	3.16 ± 0.03
		**G**	2.87 ± 0.01	2.93 ± 0.01	2.985 ± 0.008	3.042 ± 0.009	3.12 ± 0.01
	R · *CD*	**S**	3.5 ± 0.2	3.4 ± 0.1	3.4 ± 0.1	3.4 ± 0.2	3.8 ± 0.2
		**G**	3.4 ± 0.1	3.4 ± 0.1	3.4 ± 0.1	3.4 ± 0.1	3.4 ± 0.1
	R · *mCD*	**S**	3.9 ± 0.1	3.91 ± 0.09	3.9 ± 0.1	3.93 ± 0.07	3.91 ± 0.08
		**G**	3.9 ± 0.1	3.92 ± 0.08	3.93 ± 0.08	3.94 ± 0.08	4.0 ± 0.1
	C	**S**	4.52 ± 0.01	4.526 ± 0.009	4.534 ± 0.008	4.552 ± 0.006	4.584 ± 0.009
		**G**	4.51 ± 0.01	4.526 ± 0.004	4.541 ± 0.003	4.556 ± 0.004	4.577 ± 0.005
	C · *CD*	**S**	5.0 ± 0.2	5.0 ± 0.1	4.96 ± 0.09	4.9 ± 0.1	4.9 ± 0.1
		**G**	4.99 ± 0.06	4.98 ± 0.05	4.970 ± 0.05	4.96 ± 0.05	4.94 ± 0.06
	C · *mCD*	**S**	5.46 ± 0.06	5.46 ± 0.05	5.46 ± 0.04	5.46 ± 0.04	5.43 ± 0.05
		**G**	5.46 ± 0.04	5.47 ± 0.03	5.48 ± 0.03	5.49 ± 0.03	5.51 ± 0.05
μ($10^{-8}m^2.V^{-1}.s^{-1}$)	R	**S**	1.33 ± 0.04	1.51 ± 0.04	1.70 ± 0.05	1.89 ± 0.05	2.18 ± 0.06
		**G**	1.305 ± 0.003	1.483 ± 0.003	1.669 ± 0.004	1.864 ± 0.004	2.149 ± 0.005
	R · *CD*	**S**	0.92 ± 0.03	1.04 ± 0.04	1.17 ± 0.04	1.31 ± 0.05	1.51 ± 0.06
		**G**	0.87 ± 0.01	0.99 ± 0.02	1.11 ± 0.02	1.24 ± 0.02	1.43 ± 0.02
	R · *mCD*	**S**	0.741 ± 0.008	0.842 ± 0.009	0.95 ± 0.01	1.06 ± 0.01	1.22 ± 0.01
		**G**	0.751 ± 0.006	0.854 ± 0.006	0.961 ± 0.007	1.073 ± 0.008	1.237 ± 0.009
	C	**S**	1.81 ± 0.02	2.05 ± 0.02	2.31 ± 0.02	2.58 ± 0.02	2.98 ± 0.02
		**G**	1.805 ± 0.003	2.052 ± 0.003	2.310 ± 0.004	2.579 ± 0.004	2.974 ± 0.005
	C · *CD*	**S**	1.00 ± 0.04	1.14 ± 0.04	1.28 ± 0.05	1.43 ± 0.05	1.65 ± 0.06
		**G**	0.99 ± 0.01	1.13 ± 0.01	1.27 ± 0.02	1.42 ± 0.02	1.63 ± 0.02
	C · *mCD*	**S**	0.80 ± 0.02	0.91 ± 0.03	1.03 ± 0.03	1.15 ± 0.03	1.32 ± 0.04
		**G**	0.837 ± 0.006	0.952 ± 0.006	1.071 ± 0.007	1.196 ± 0.008	1.379 ± 0.009

Table S3: Fitted values from the step-by-step fitting process. Units: μ_X in 10^{-8} $m^2.V^{-1}.s^{-1}$; $\eta\mu_{XL}$ in 10^{-11} $N.V^{-1}$; K_{X^-} in M^{-1}

Step	Conditions	Compound	Parameter	$\theta = 15°C$	$\theta = 20°C$	$\theta = =25°C$	$\theta = 30°C$	$\theta = =37°C$	Mean $\eta\mu$
1	$pH = 7$, [L]=0 M	R^-	μ_X	1.338 ± 0.003	1.518 ± 0.003	1.71 ± 0.02	1.91 ± 0.02	2.1550 ± 0.005	1.51 ± 0.04
		C^-		1.862 ± 0.003	2.100 ± 0.0050	2.34 ± 0.02	2.55 ± 0.02	2.924 ± 0.006	2.06 ± 0.02
2	$pH = 7$, various [L]	$R^- \cdot CD$	$\eta\mu_{XL}$	1.07 ± 0.03	1.00 ± 0.02	1.02 ± 0.01	1.09 ± 0.02	1.05 ± 0.02	1.05 ± 0.04
			K_{X^-}	330 ± 40	210 ± 20	220 ± 20	220 ± 20	230 ± 20	—
		$R^- \cdot mCD$	$\eta\mu_{XL}$	0.84 ± 0.01	0.841 ± 0.008	0.850 ± 0.009	0.834 ± 0.006	0.856 ± 0.007	0.843 ± 0.009
			K_{X^-}	430 ± 40	410 ± 30	410 ± 30	300 ± 10	357 ± 20	—
		$C^- \cdot CD$	$\eta\mu_{XL}$	1.15 ± 0.04	1.11 ± 0.03	1.12 ± 0.03	1.21 ± 0.03	1.13 ± 0.04	1.14 ± 0.04
			K_{X^-}	190 ± 20	160 ± 10	150 ± 10	160 ± 10	140 ± 10	—
		$C^- \cdot mCD$	$\eta\mu_{XL}$	0.89 ± 0.02	0.90 ± 0.01	0.92 ± 0.01	0.91 ± 0.01	0.96 ± 0.01	0.92 ± 0.03
			K_{X^-}	172 ± 8	158 ± 6	146 ± 5	134 ± 5	132 ± 5	—
3	[L]=0 or 15 mM, various pH	R^-	$pK_{a,app}$	2.93 ± 0.01	2.95 ± 0.02	2.99 ± 0.03	3.05 ± 0.02	3.16 ± 0.03	—
		$R^- \cdot CD$		3.40 ± 0.03	3.35 ± 0.03	3.34 ± 0.03	3.32 ± 0.03	3.34 ± 0.03	—
		$R^- \cdot mCD$		3.88 ± 0.01	3.857 ± 0.009	3.855 ± 0.008	3.85 ± 0.02	3.85 ± 0.02	—
		C^-		4.52 ± 0.01	4.526 ± 0.009	4.534 ± 0.008	4.552 ± 0.006	4.584 ± 0.009	—
		$C^- \cdot CD$		4.919 ± 0.009	4.891 ± 0.006	4.866 ± 0.005	4.84 ± 0.01	4.814 ± 0.006	—
		$C^- \cdot mCD$		5.337 ± 0.007	5.328 ± 0.005	5.321 ± 0.009	5.31 ± 0.01	5.29 ± 0.02	—

Table S4: Residuals statistics. The last column labelled "Ideal" gives the ideal values that would be obtained in the case of residuals with normally distributed noise. Residual statistics: the skewness represent the symmetry residuals around the mean; the kurtosis represent the presence of distribution tails (positive values indicating larger tails than for the normal distribution). All statistics were performed from electrophoretic mobilities in 10^{-8} $m^2.V^{-1}.s^{-1}$ units.

		Step-by-step	Global	Ideal
Residual statistics	Mean	-0.0008	0.0011	0
	σ	0.0272	0.0259	σ
	Skewness	-0.62	-0.34	0
	Kurtosis	4.94	4.25	0
Linear regression μ_{exp}—μ_{fitted}	Intercept	-0.007	0.003	0
	Slope	1.006	1.002	1
	R^2	0.9982	0.9981	1
Student's t-distribution	ν	2.73	4.30	∞
	Localization	0.0004	0.0009	0
	Scale	0.0165	0.0191	σ

Table S5: $\eta \cdot \mu$ viscosity-mobility product, $\eta \cdot \mu^{\infty}$ viscosity-mobility product corrected at I = 0 (10^{-11} N.V^{-1}), and equivalent hydrodynamic radius R_e (Å).

	$\eta \cdot \mu$		$\eta \cdot \mu^{\infty}$		R_e	
	*p*H = 7 Fits	Global fit	*p*H = 7 Fits	Global fit	*p*H = 7 Fits	Global fit
R	1.51 ± 0.04	1.486 ± 0.003	1.72 ± 0.01	1.690 ± 0.005	4.94 ± 0.04	5.02 ± 0.01
R · *CD*	1.05 ± 0.04	0.99 ± 0.02	1.24 ± 0.04	1.19 ± 0.02	6.8 ± 0.2	7.2 ± 0.1
R · *mCD*	0.843 ± 0.009	0.856 ± 0.006	1.036 ± 0.009	1.048 ± 0.007	8.2 ± 0.1	8.10 ± 0.05
C	2.06 ± 0.02	2.056 ± 0.003	2.27 ± 0.04	2.257 ± 0.005	3.7 ± 0.1	3.76 ± 0.01
C · *CD*	1.14 ± 0.04	1.13 ± 0.01	1.34 ± 0.04	1.33 ± 0.01	6.3 ± 0.2	6.38 ± 0.07
C · *mCD*	0.92 ± 0.03	0.954 ± 0.006	1.11 ± 0.03	1.148 ± 0.007	7.6 ± 0.2	7.39 ± 0.04
