# Supplementary material for: Encapsulation of phenolic acids into cyclodextrins: A global statistical analysis of the effects of pH, temperature and concentrations on binding constants measured by ACE methods
Source: Electrophoresis. 2022 Jul 10;43(23-24):2290–301. doi: 10.1002/elps.202200075 (PMC10083966; doi:10.1002/elps.202200075)
Supplement: Supplementary file 2 — The full data and python scripts used in this work are provided within a single Python‐script.rar archive file. [file ELPS-43-2290-s002.pdf]

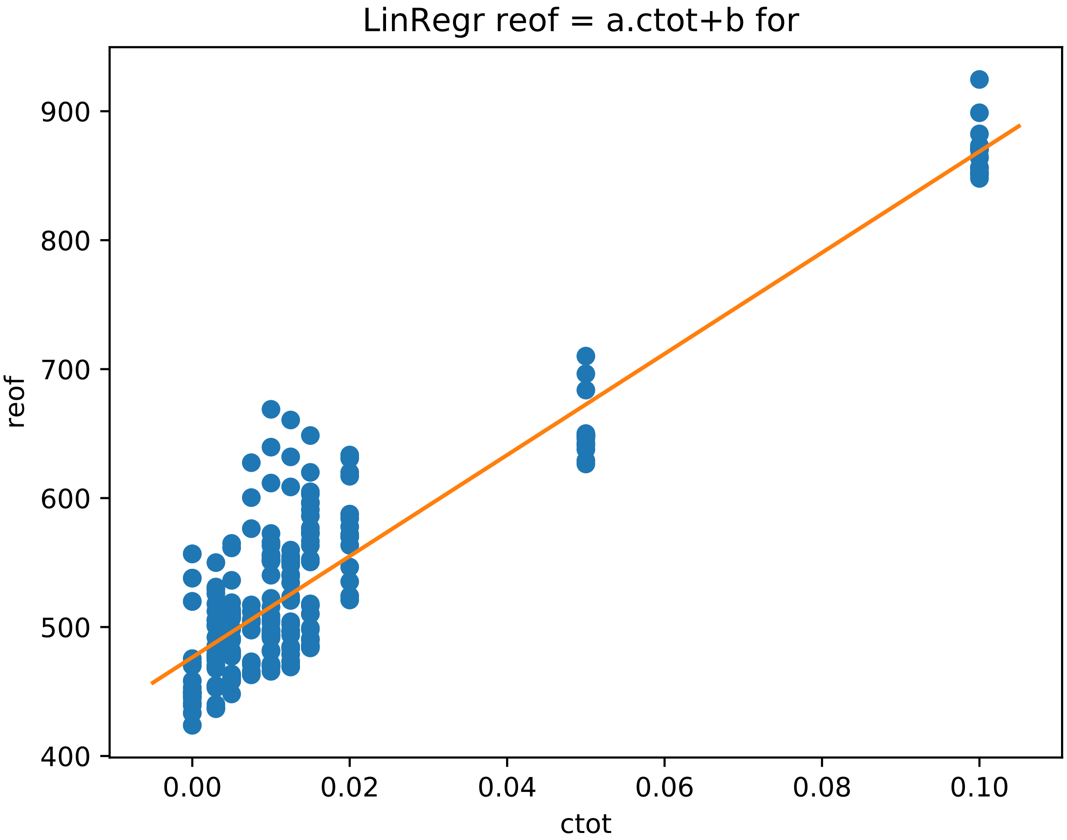
LinRegr reof = a.ctot+b for
reof
ctot
900
800
700
600
500
400
0.00
0.02
0.04
0.06
0.08
0.10

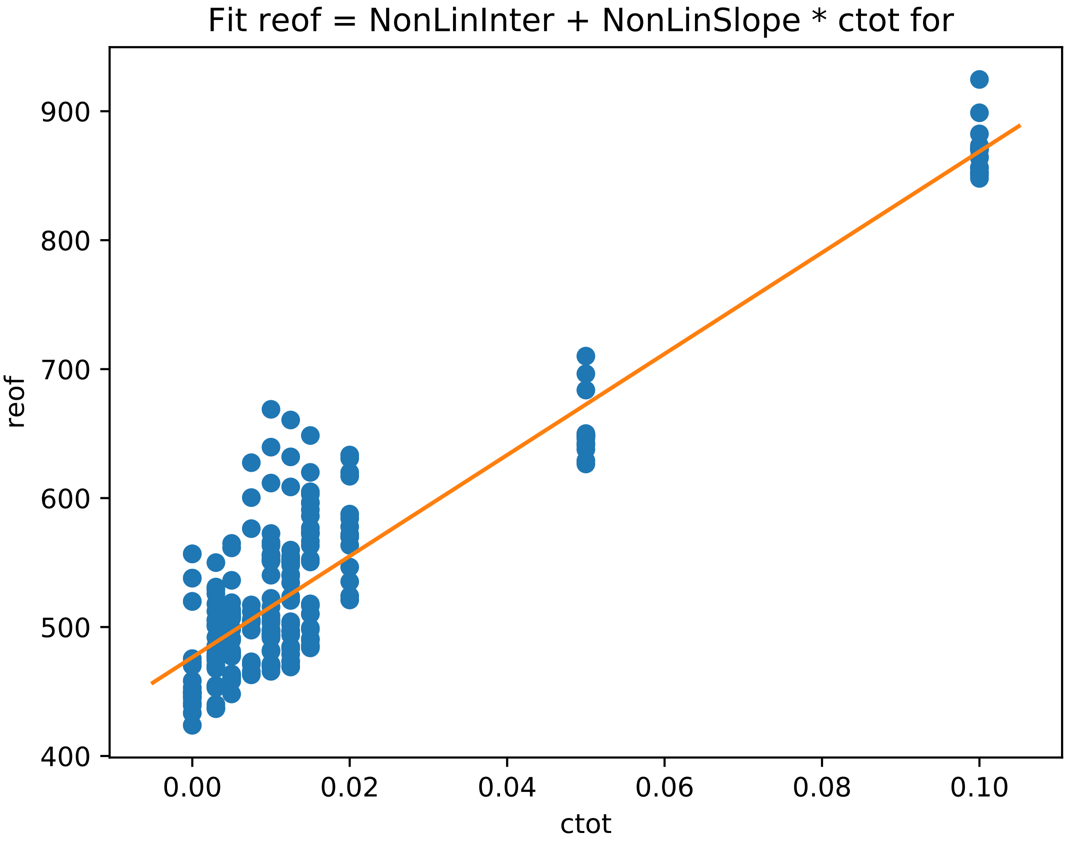
Fit reof = NonLinInter + NonLinSlope * ctot for
reof
900
800
700
600
500
400
0.00
0.02
0.04
0.06
0.08
0.10
ctot

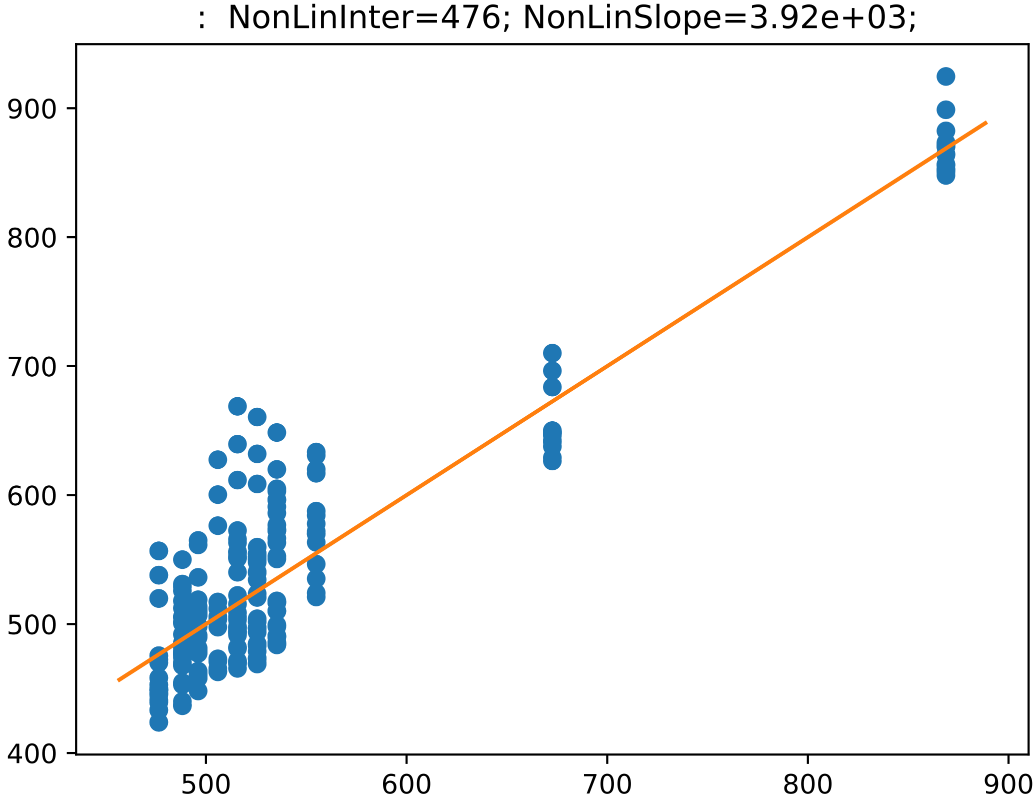

: NonLinInter=476; NonLinSlope=3.92e+03;
900
800
700
600
500
400
500
600
700
800
900

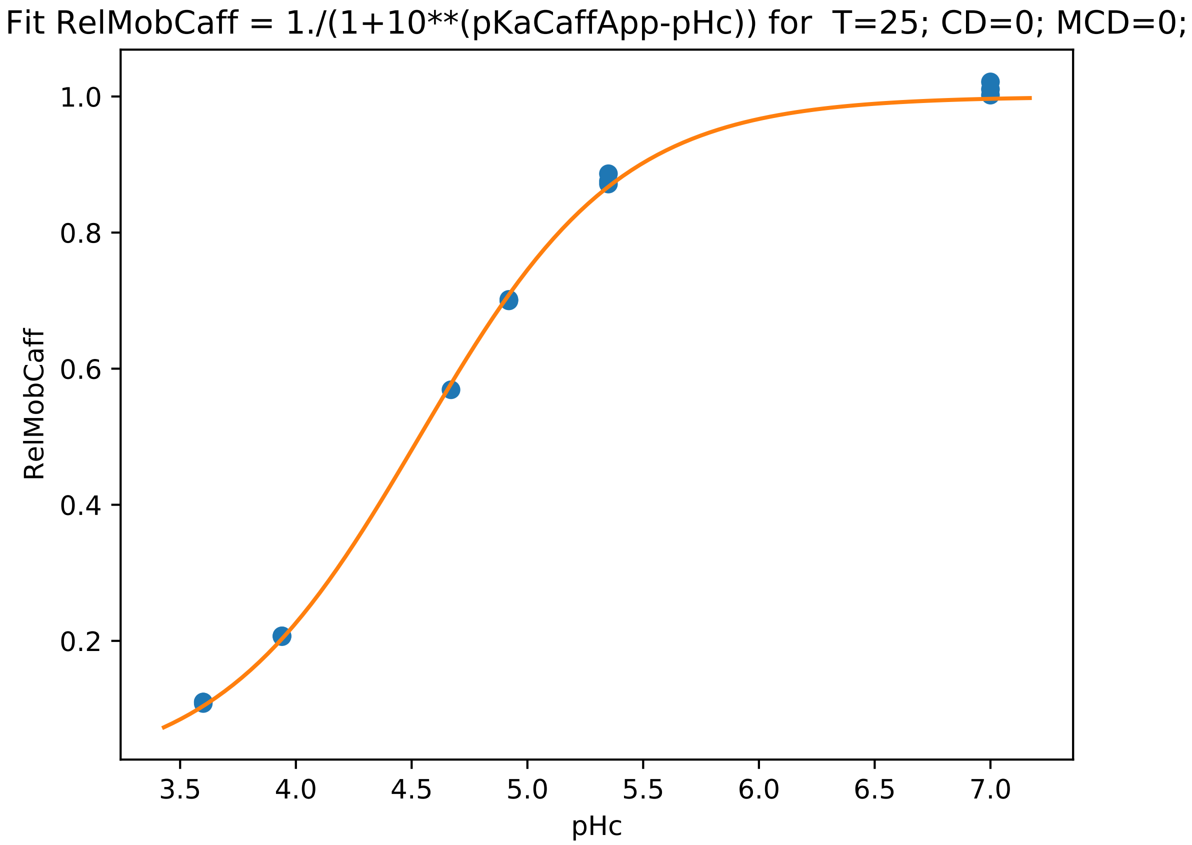
Fit RelMobCaff = 1./(1+10**(pKaCaffApp-pHc)) for T=25; CD=0; MCD=0;
RelMobCaff
pHc
1.0
0.8
0.6
0.4
0.2
3.5
4.0
4.5
5.0
5.5
6.0
6.5
7.0

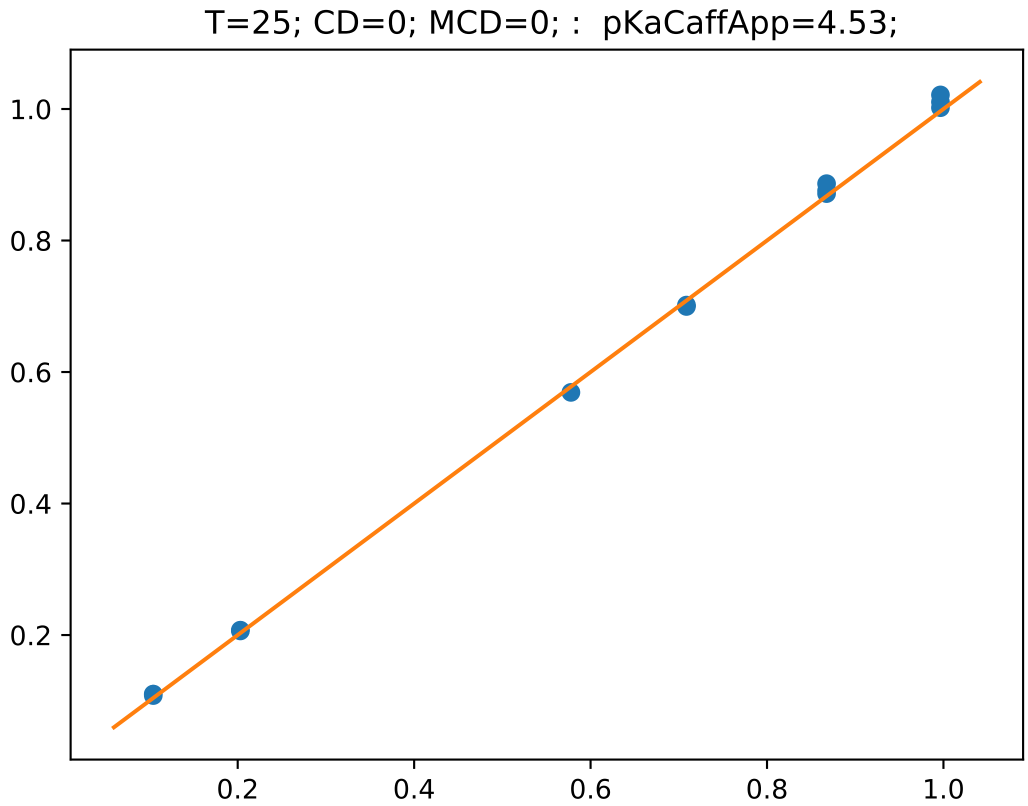

T=25; CD=0; MCD=0; : pKaCaffApp=4.53;
1.0
0.8
0.6
0.4
0.2
0.2
0.4
0.6
0.8
1.0

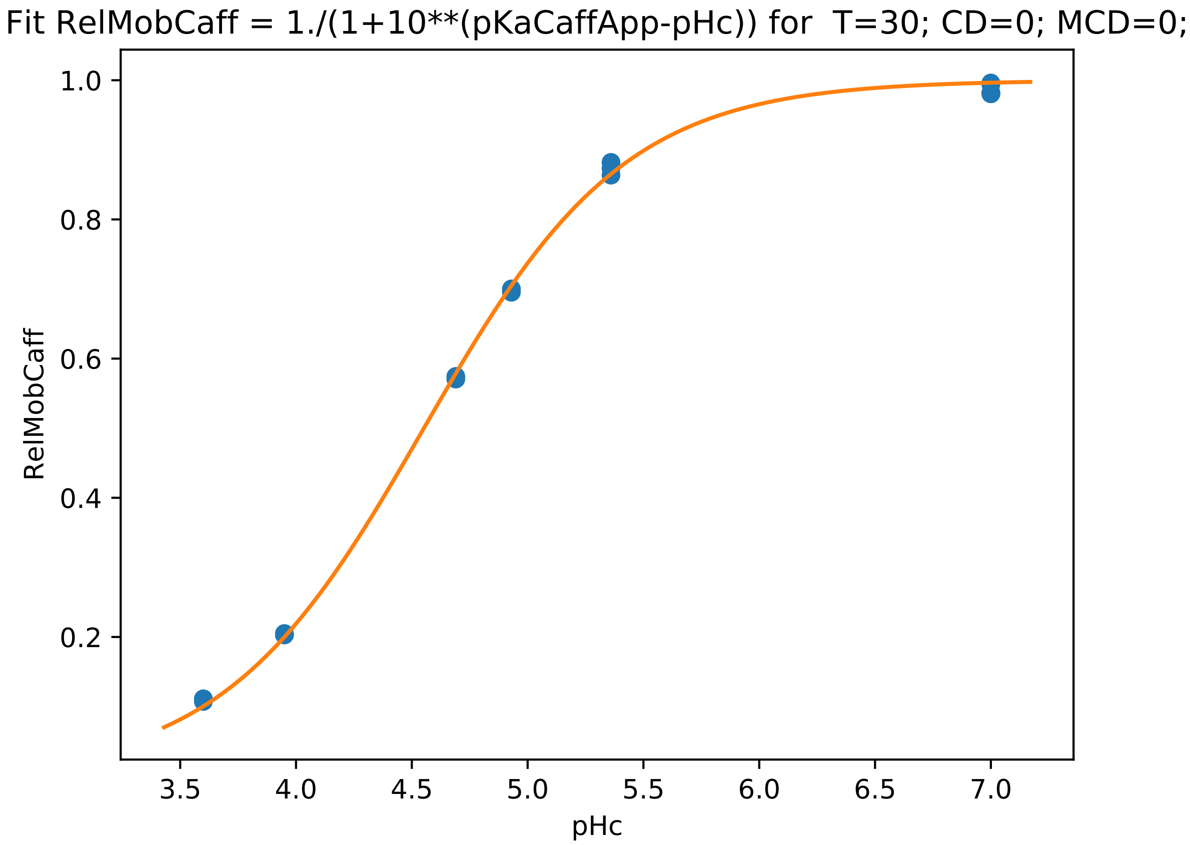
Fit RelMobCaff = 1./(1+10**(pKaCaffApp-pHc)) for T=30; CD=0; MCD=0;
RelMobCaff
pHc
1.0
0.8
0.6
0.4
0.2
3.5
4.0
4.5
5.0
5.5
6.0
6.5
7.0

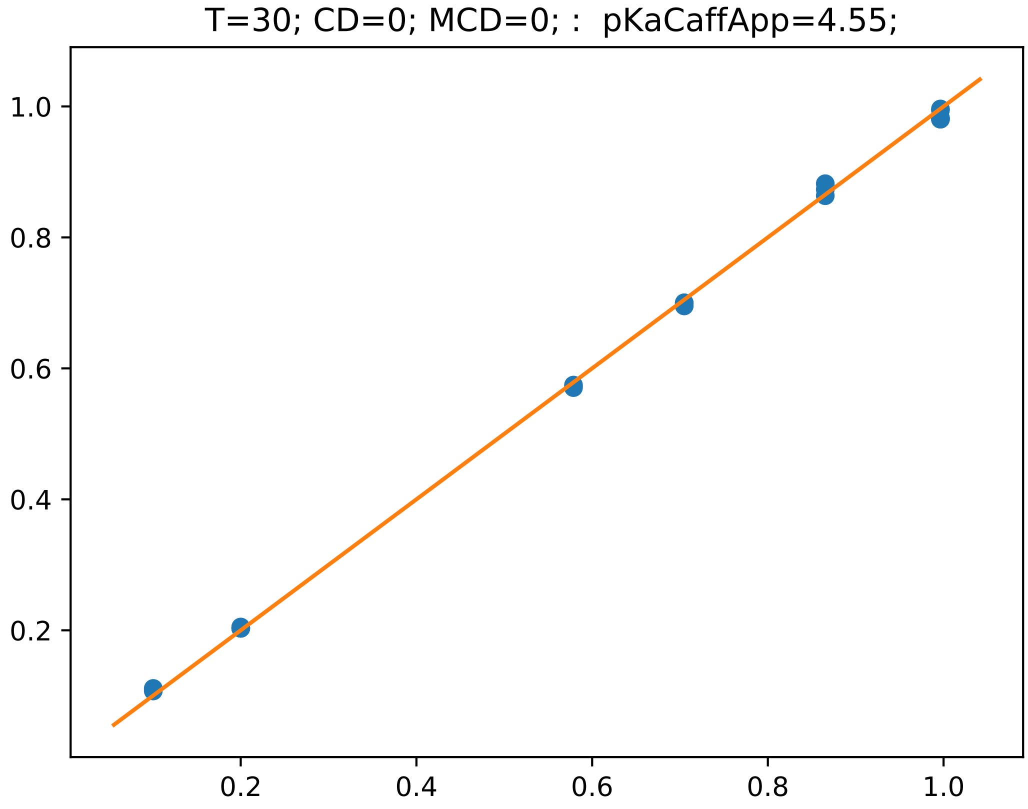
T=30; CD=0; MCD=0; : pKaCaffApp=4.55;
1.0
0.8
0.6
0.4
0.2
0.2
0.4
0.6
0.8
1.0

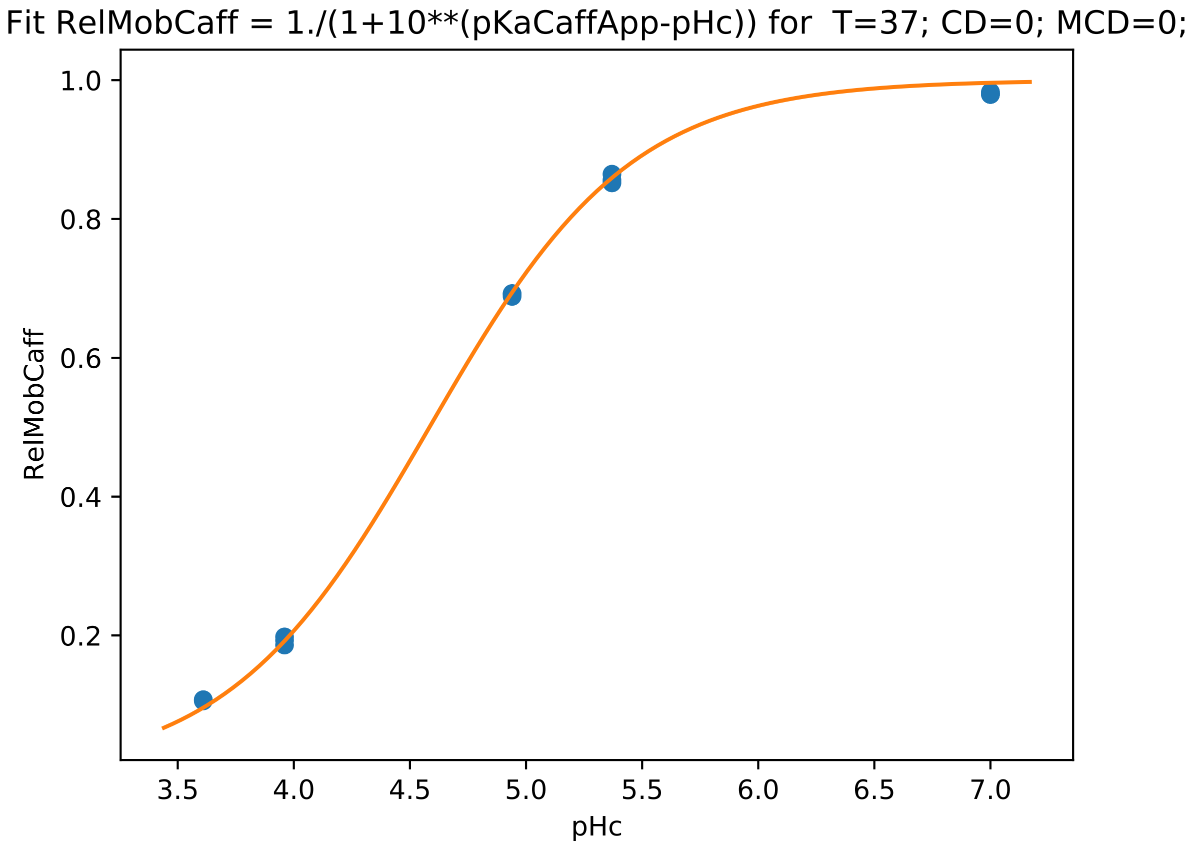

Fit RelMobCaff = 1./(1+10**(pKaCaffApp-pHc)) for T=37; CD=0; MCD=0;
1.0
0.8
0.6
0.4
0.2
RelMobCaff
3.5
4.0
4.5
5.0
5.5
6.0
6.5
7.0
pHc

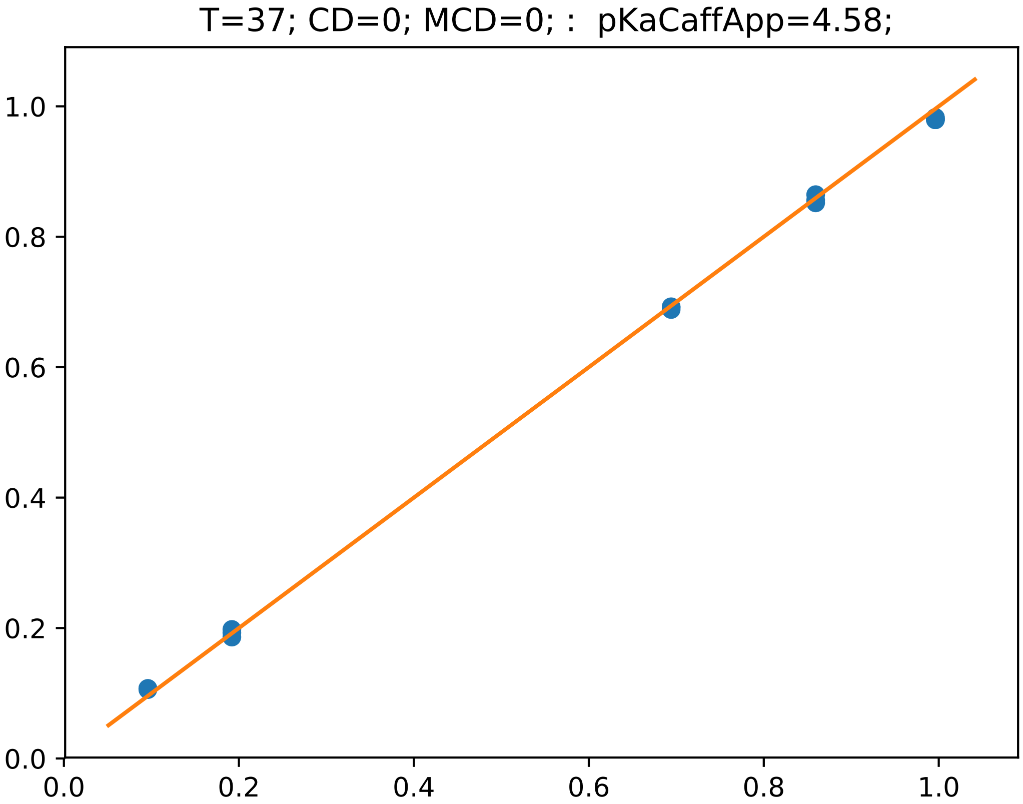
T=37; CD=0; MCD=0; : pKaCaffApp=4.58;
0.0
0.2
0.4
0.6
0.8
1.0
0.0
0.2
0.4
0.6
0.8
1.0

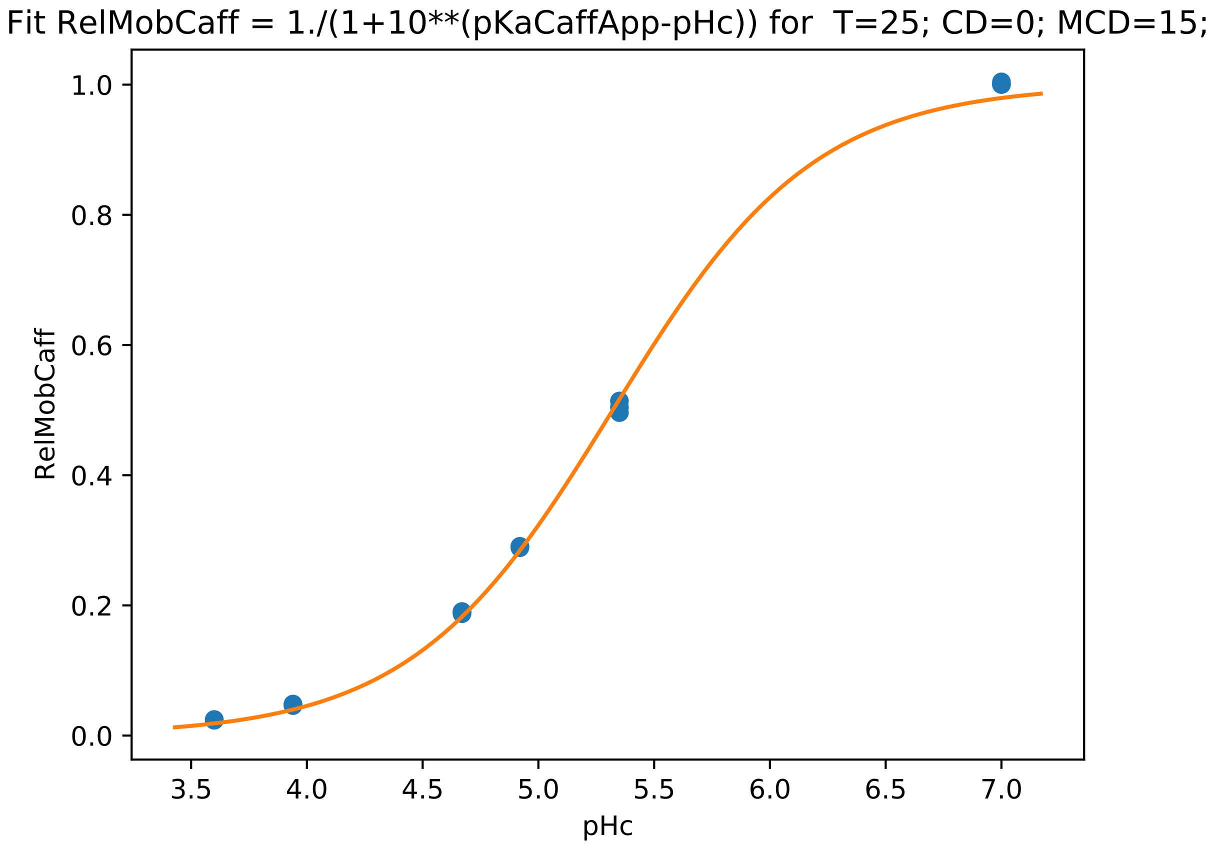
Fit RelMobCaff = 1./(1+10**(pKaCaffApp-pHc)) for T=25; CD=0; MCD=15;
RelMobCaff
0.0
0.2
0.4
0.6
0.8
1.0
3.5
4.0
4.5
5.0
5.5
6.0
6.5
7.0
pHc

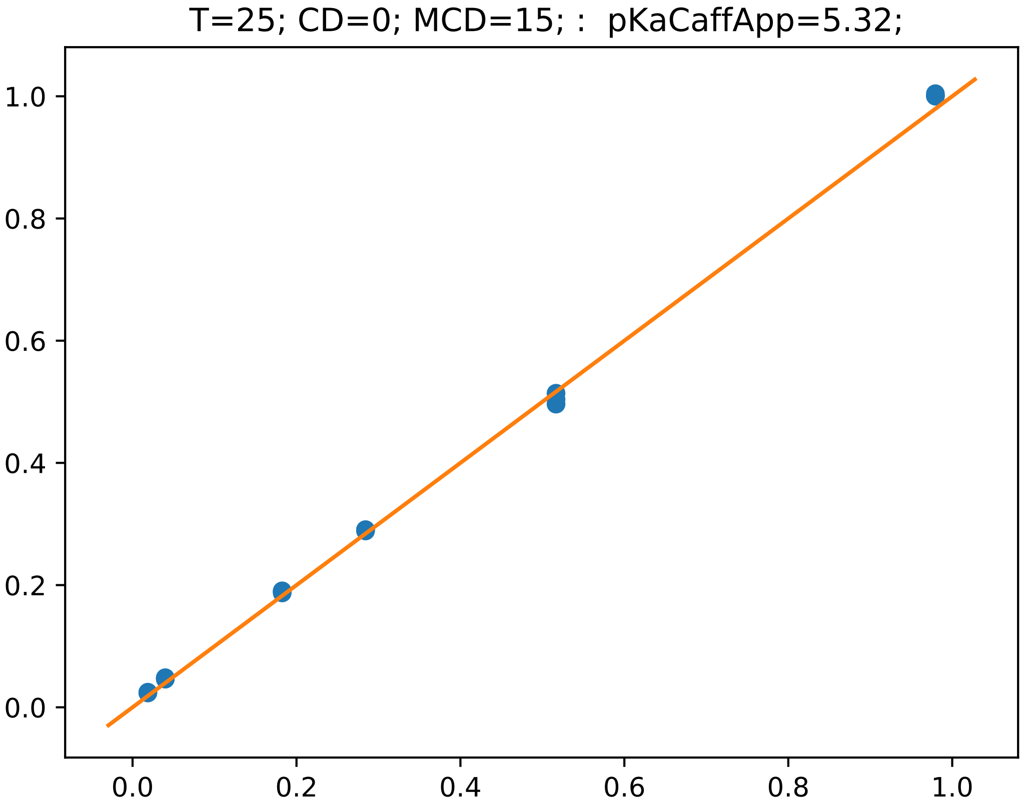
T=25; CD=0; MCD=15; : pKaCaffApp=5.32;
1.0
0.8
0.6
0.4
0.2
0.0
0.0
0.2
0.4
0.6
0.8
1.0

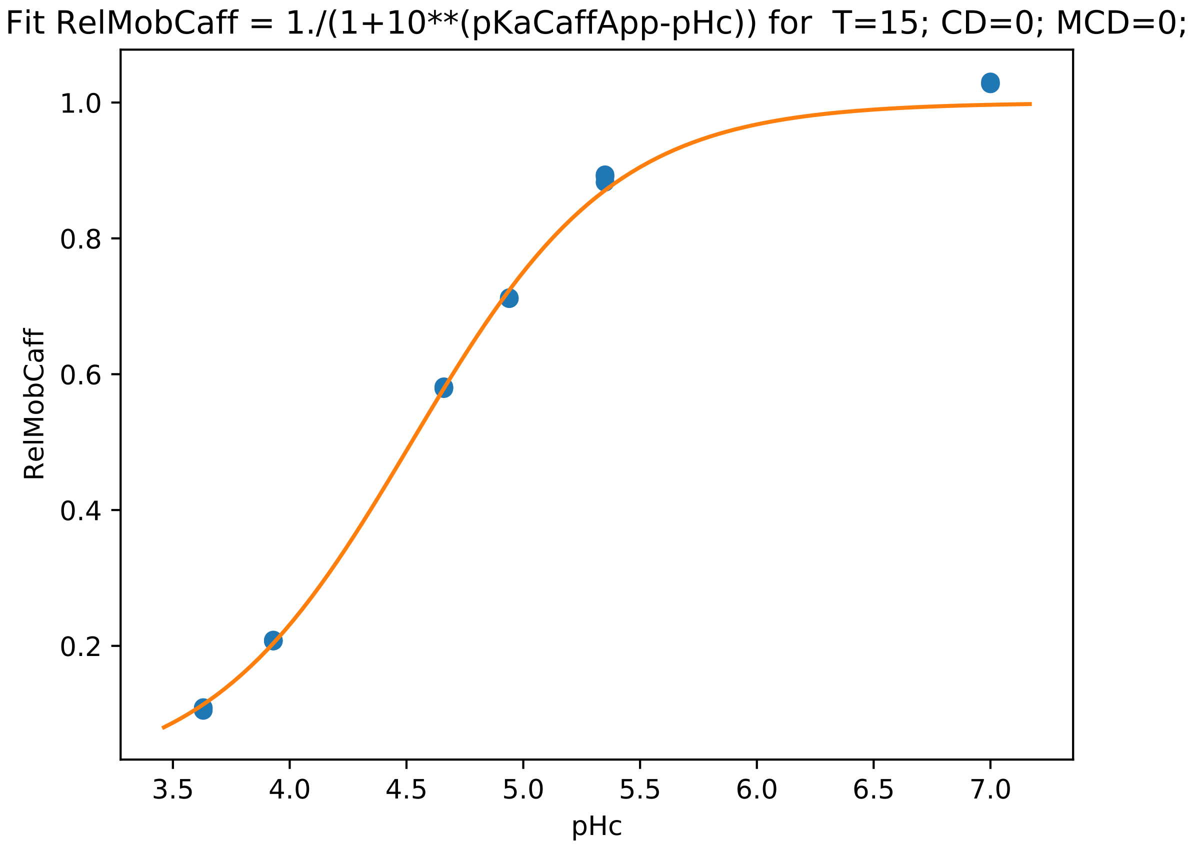

Fit RelMobCaff = 1./(1+10**(pKaCaffApp-pHc)) for T=15; CD=0; MCD=0;
RelMobCaff
pHc
1.0
0.8
0.6
0.4
0.2
3.5
4.0
4.5
5.0
5.5
6.0
6.5
7.0

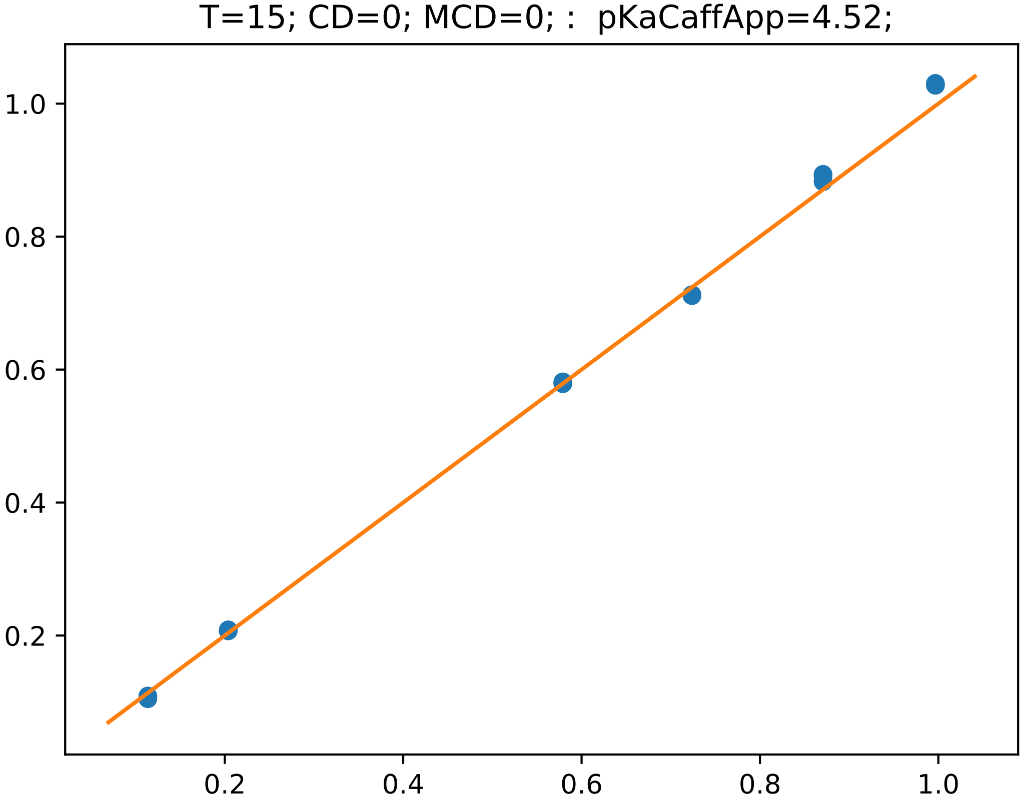
T=15; CD=0; MCD=0; : pKaCaffApp=4.52;
1.0
0.8
0.6
0.4
0.2
0.2
0.4
0.6
0.8
1.0

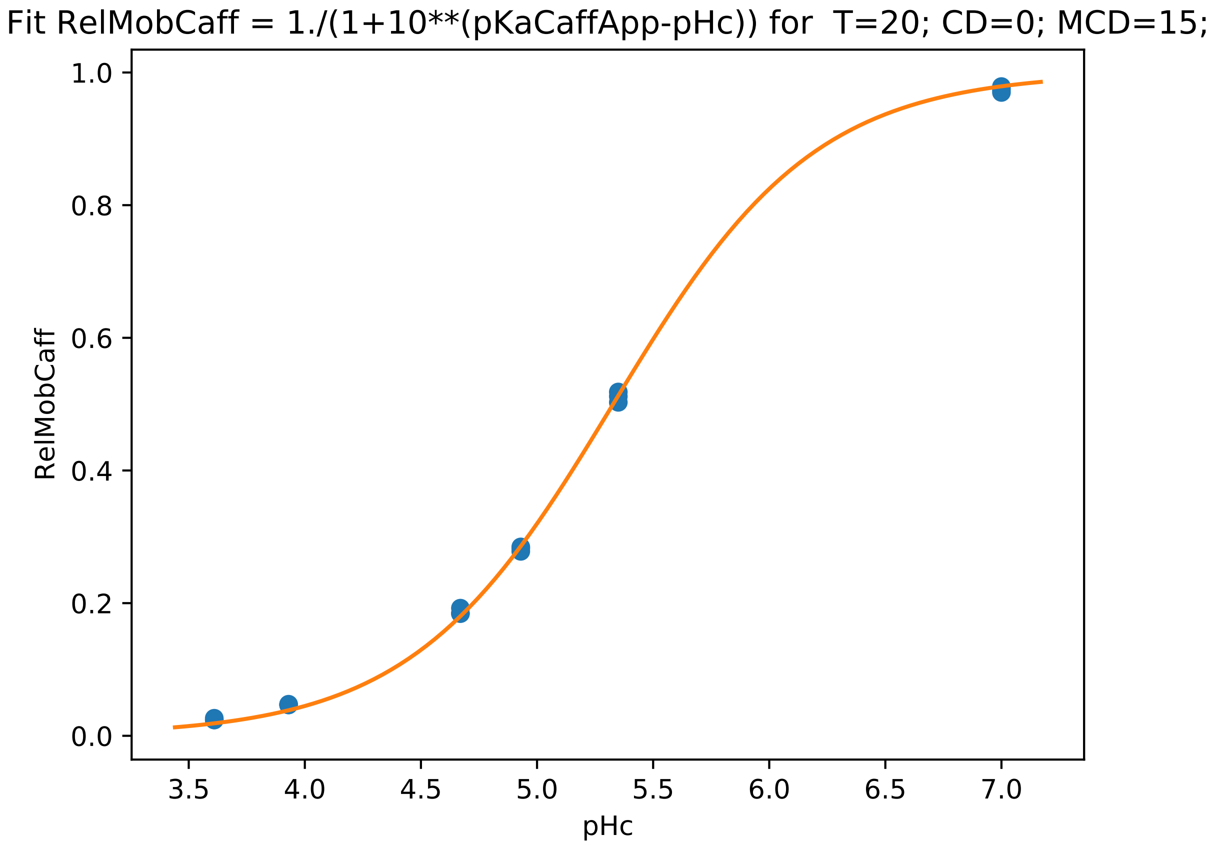

Fit RelMobCaff = 1./(1+10**(pKaCaffApp-pHc)) for T=20; CD=0; MCD=15;
RelMobCaff
pHc
1.0
0.8
0.6
0.4
0.2
0.0
3.5
4.0
4.5
5.0
5.5
6.0
6.5
7.0

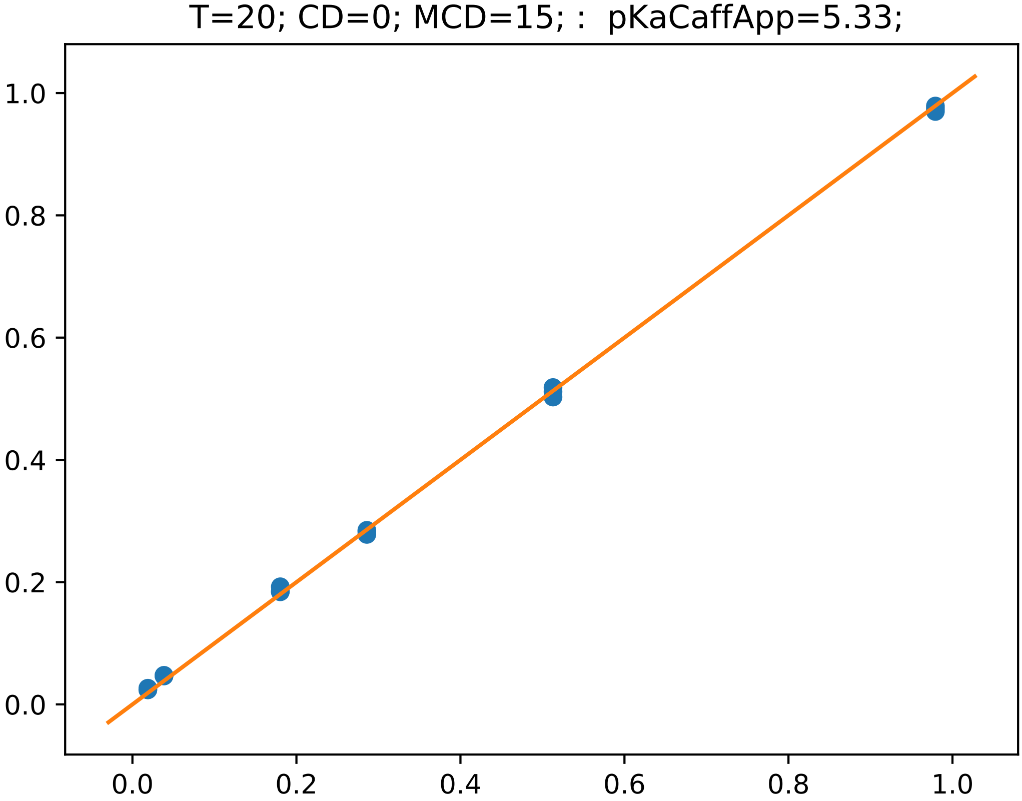
T=20; CD=0; MCD=15; : pKaCaffApp=5.33;
1.0
0.8
0.6
0.4
0.2
0.0
0.0
0.2
0.4
0.6
0.8
1.0

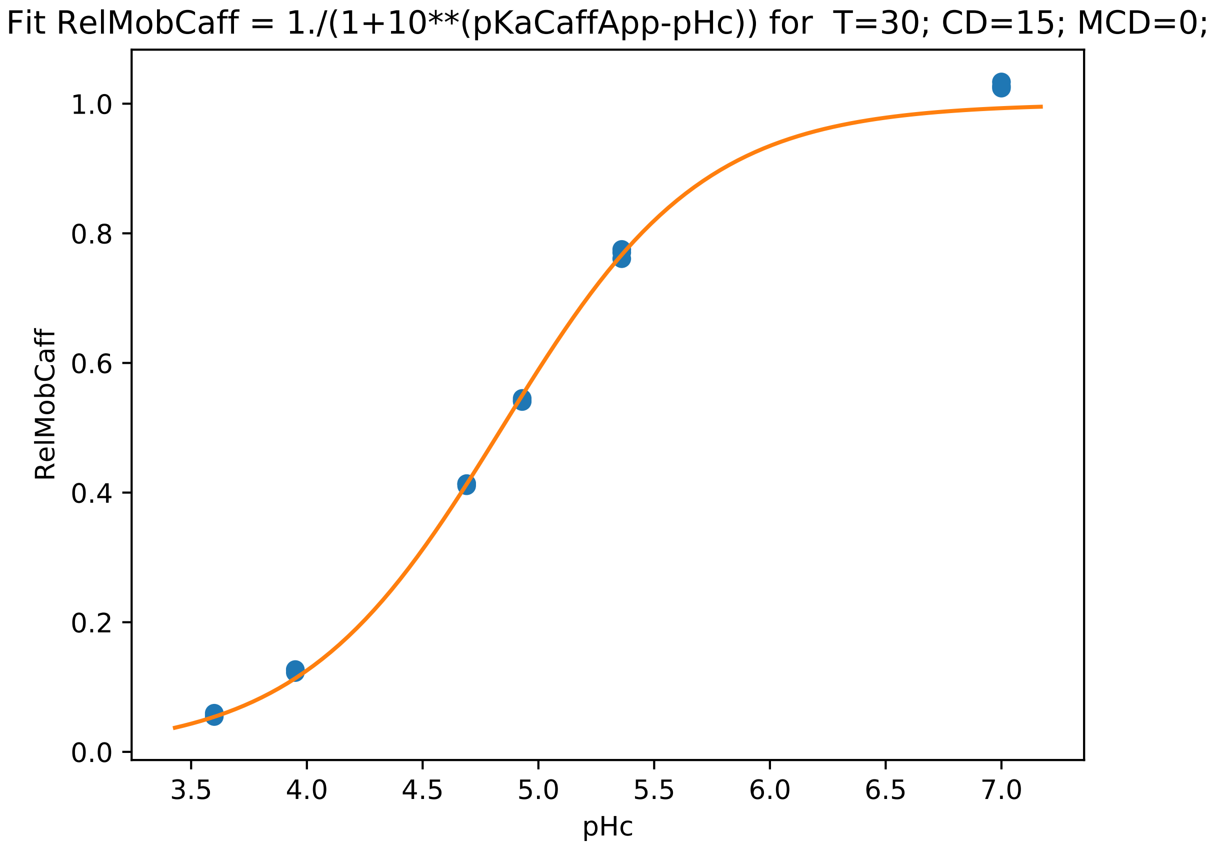
Fit RelMobCaff = 1./(1+10**(pKaCaffApp-pHc)) for T=30; CD=15; MCD=0;
RelMobCaff
pHc
0.0
0.2
0.4
0.6
0.8
1.0
3.5
4.0
4.5
5.0
5.5
6.0
6.5
7.0

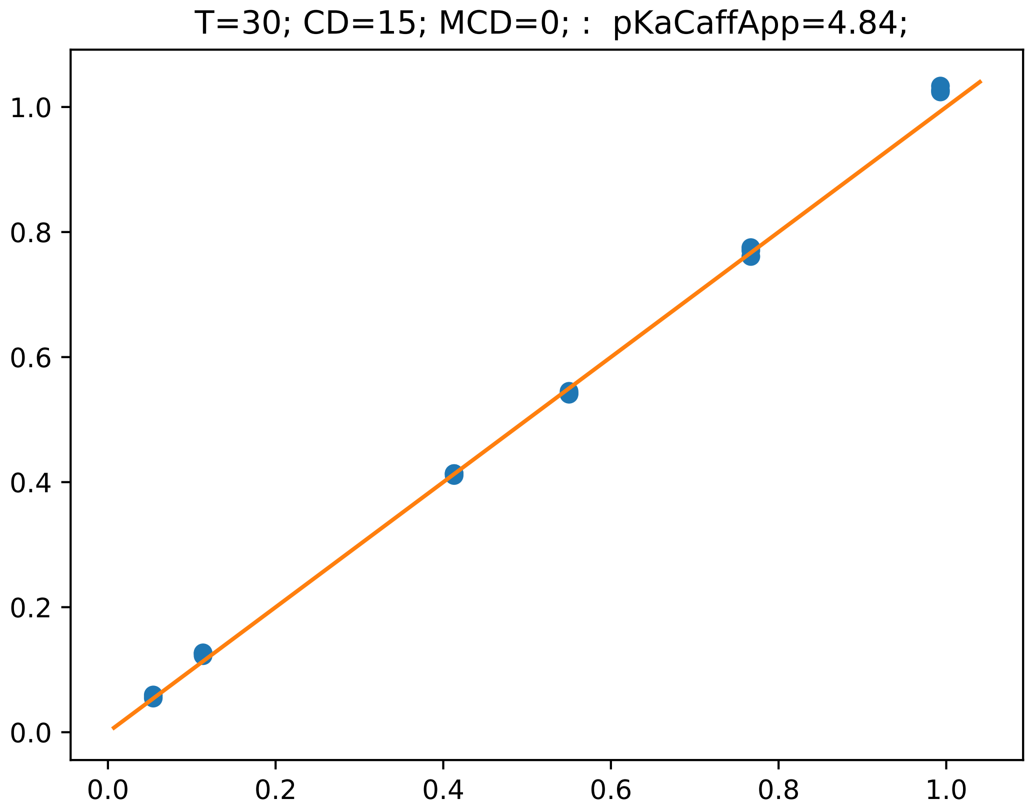
T=30; CD=15; MCD=0; : pKaCaffApp=4.84;
0.0
0.2
0.4
0.6
0.8
1.0
0.0
0.2
0.4
0.6
0.8
1.0

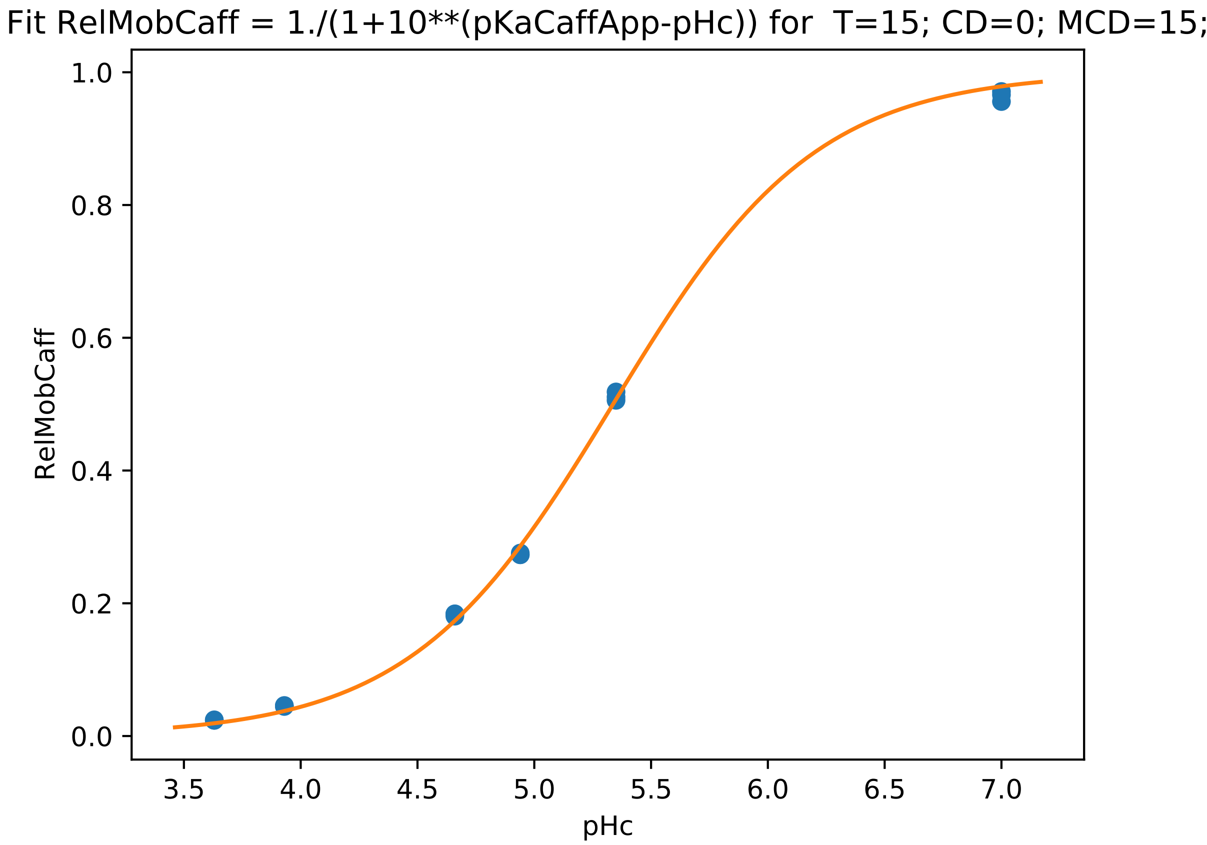
Fit RelMobCaff = 1./(1+10**(pKaCaffApp-pHc)) for T=15; CD=0; MCD=15;
RelMobCaff
pHc
1.0
0.8
0.6
0.4
0.2
0.0
3.5
4.0
4.5
5.0
5.5
6.0
6.5
7.0

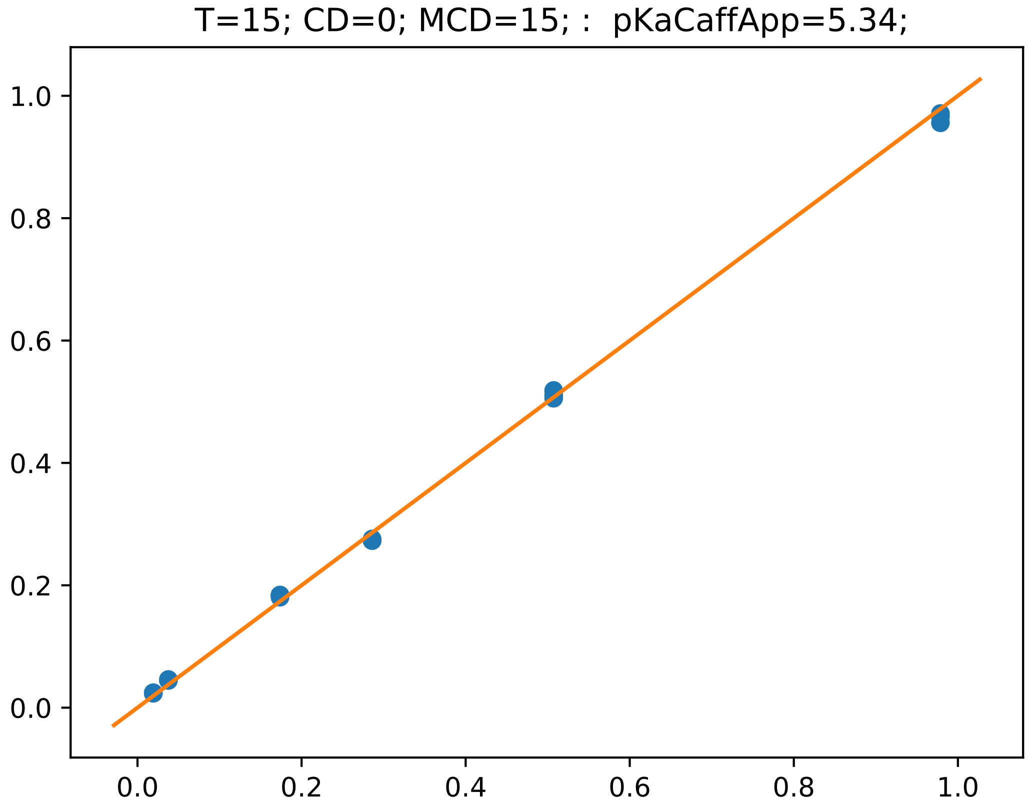
T=15; CD=0; MCD=15; : pKaCaffApp=5.34;
1.0
0.8
0.6
0.4
0.2
0.0
0.0
0.2
0.4
0.6
0.8
1.0

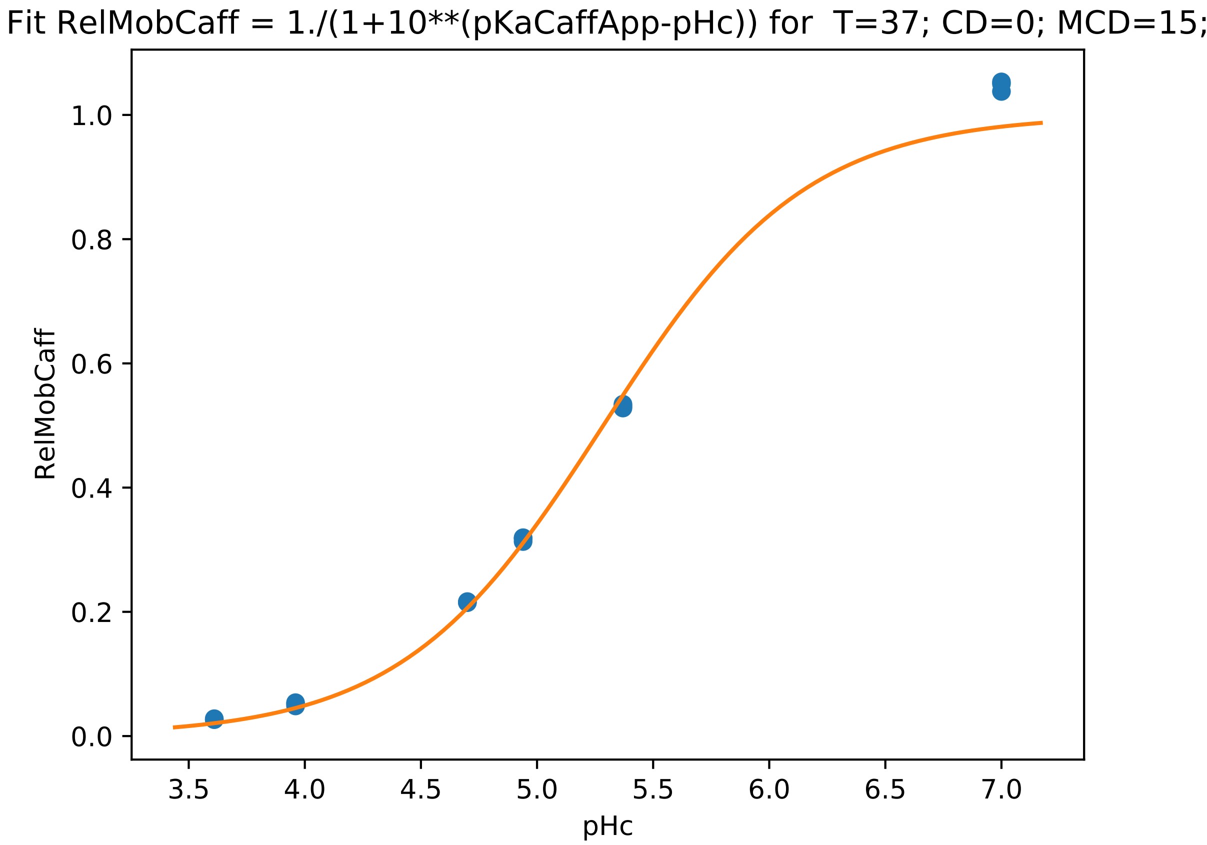

Fit RelMobCaff = 1./(1+10**(pKaCaffApp-pHc)) for T=37; CD=0; MCD=15;
RelMobCaff
pHc
1.0
0.8
0.6
0.4
0.2
0.0
3.5
4.0
4.5
5.0
5.5
6.0
6.5
7.0

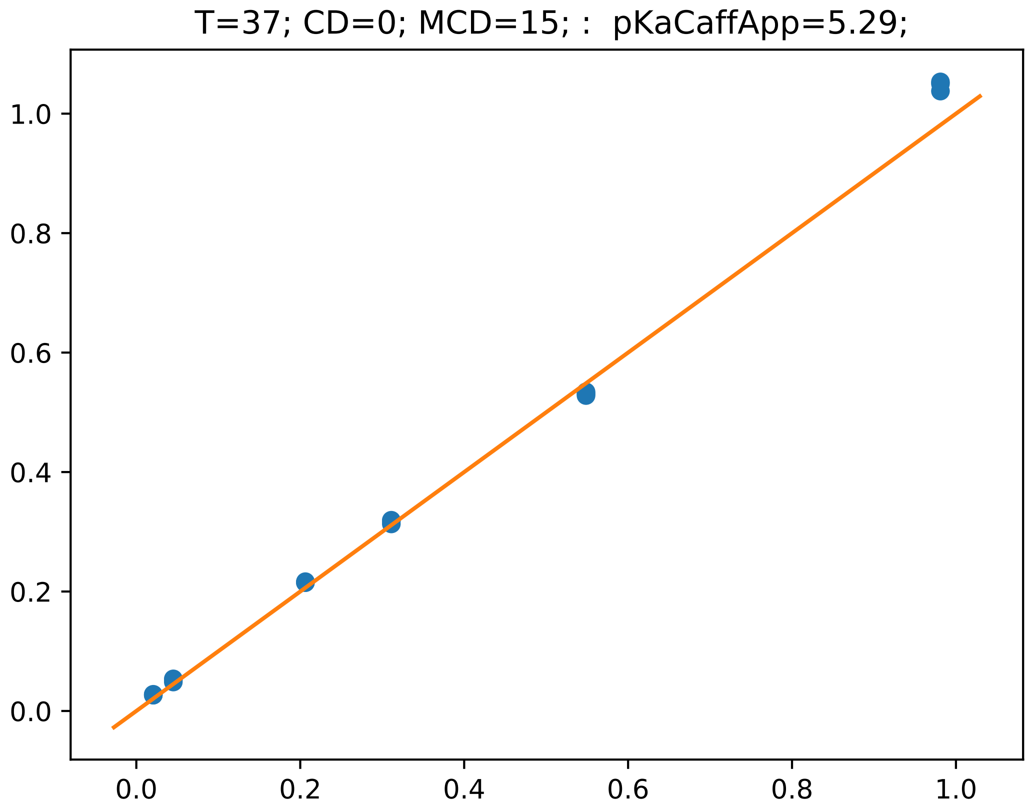
T=37; CD=0; MCD=15; : pKaCaffApp=5.29;
1.0
0.8
0.6
0.4
0.2
0.0
0.0
0.2
0.4
0.6
0.8
1.0

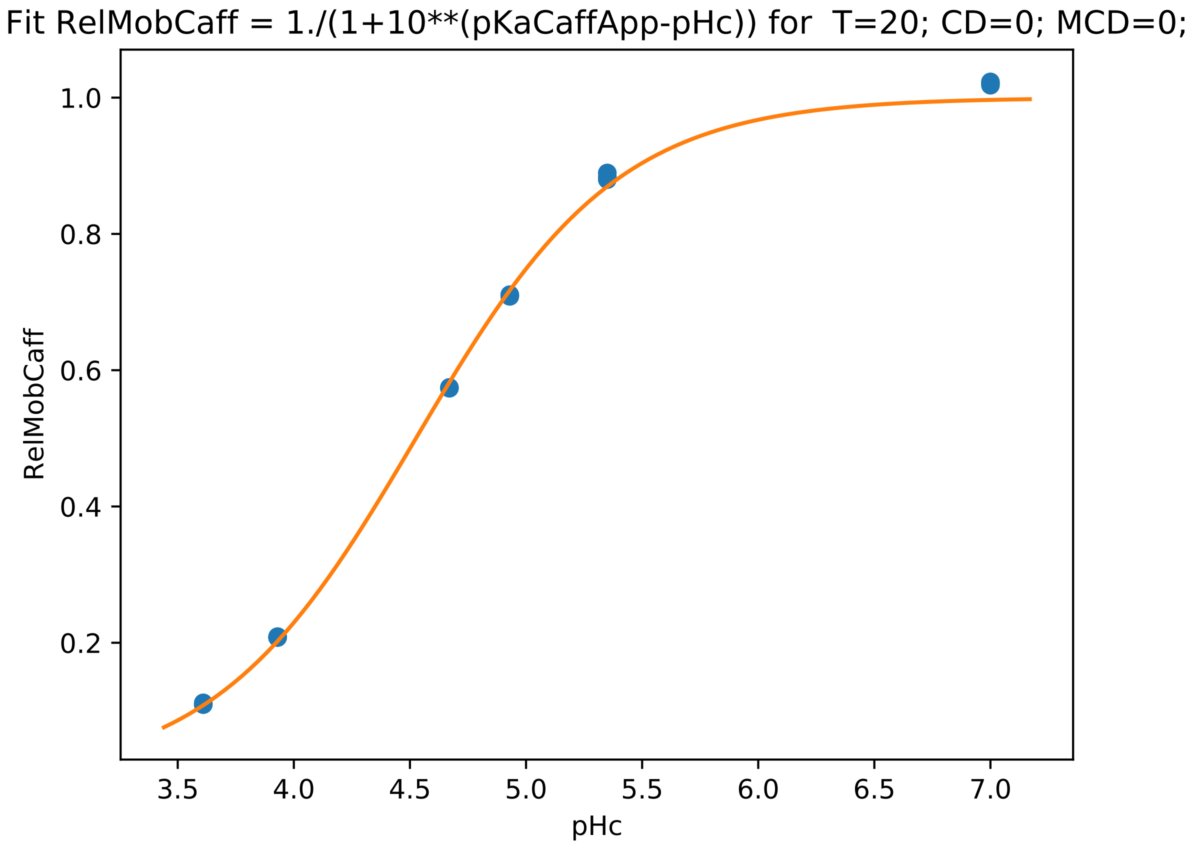

Fit RelMobCaff = 1./(1+10**(pKaCaffApp-pHc)) for T=20; CD=0; MCD=0;
RelMobCaff
pHc
1.0
0.8
0.6
0.4
0.2
3.5
4.0
4.5
5.0
5.5
6.0
6.5
7.0

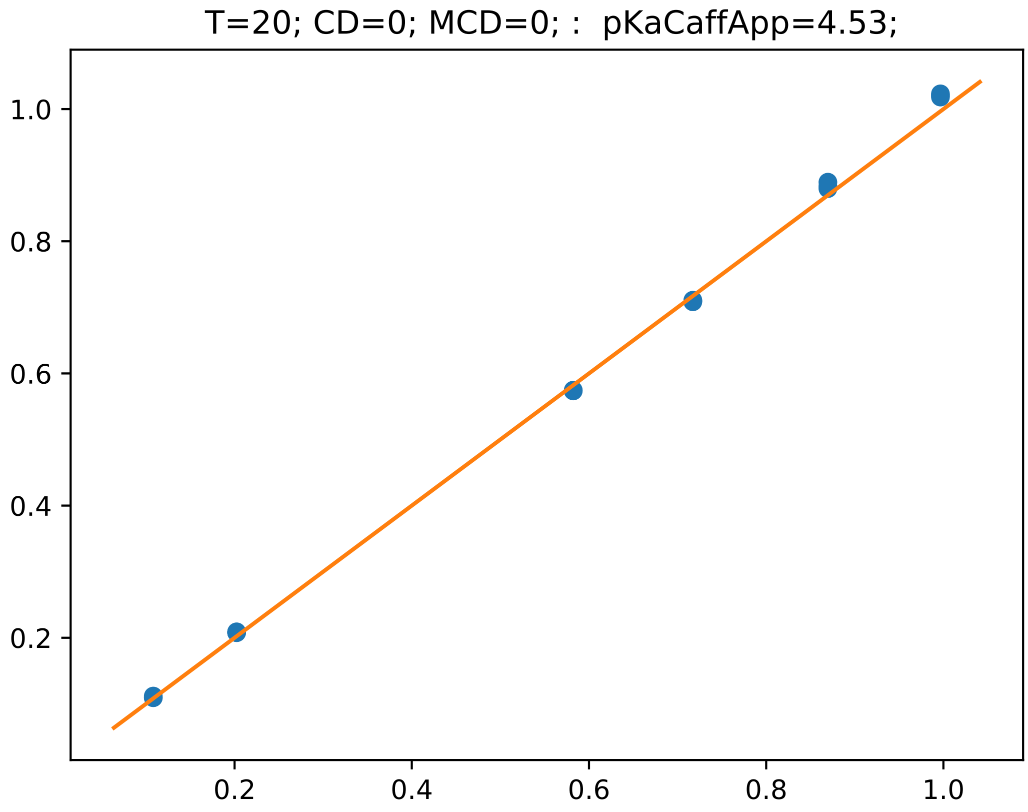

T=20; CD=0; MCD=0; : pKaCaffApp=4.53;
1.0
0.8
0.6
0.4
0.2
0.2
0.4
0.6
0.8
1.0

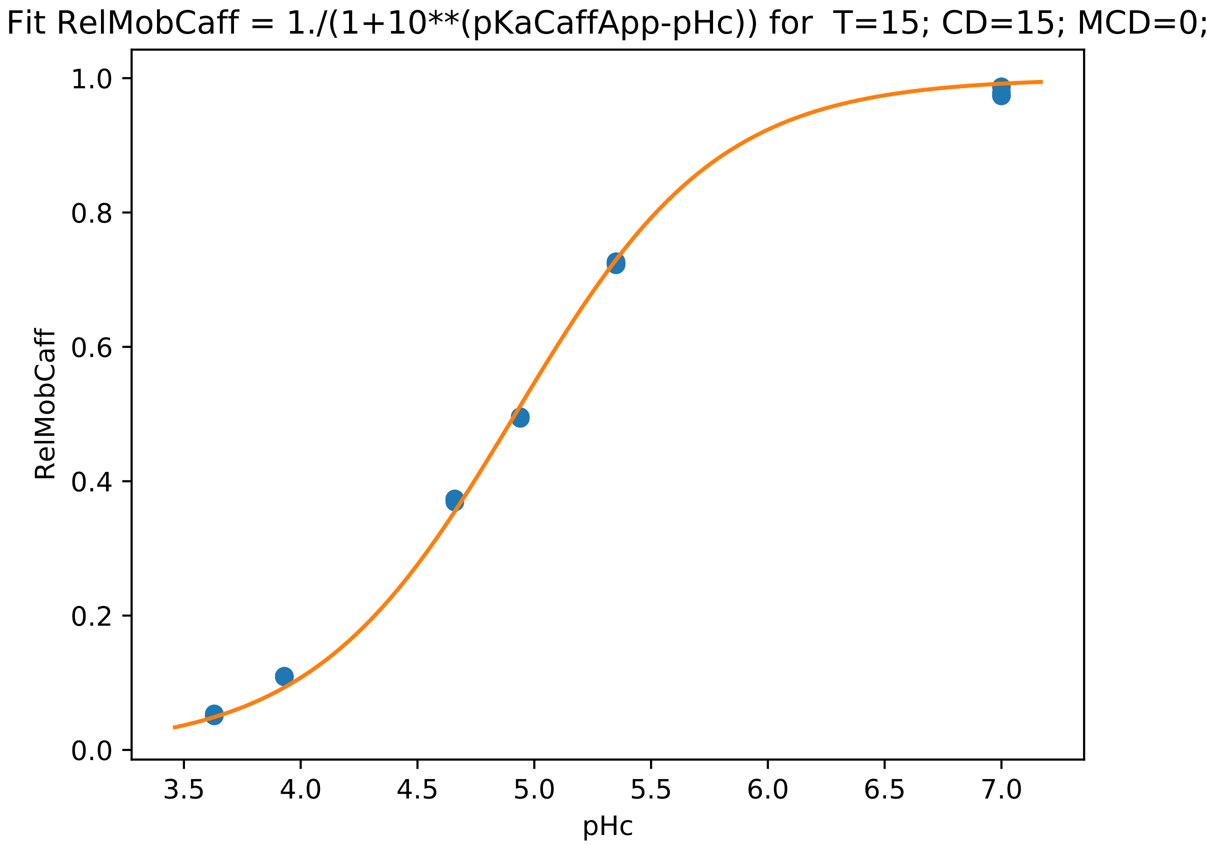
Fit RelMobCaff = 1./(1+10**(pKaCaffApp-pHc)) for T=15; CD=15; MCD=0;
RelMobCaff
pHc
0.0
0.2
0.4
0.6
0.8
1.0
3.5
4.0
4.5
5.0
5.5
6.0
6.5
7.0

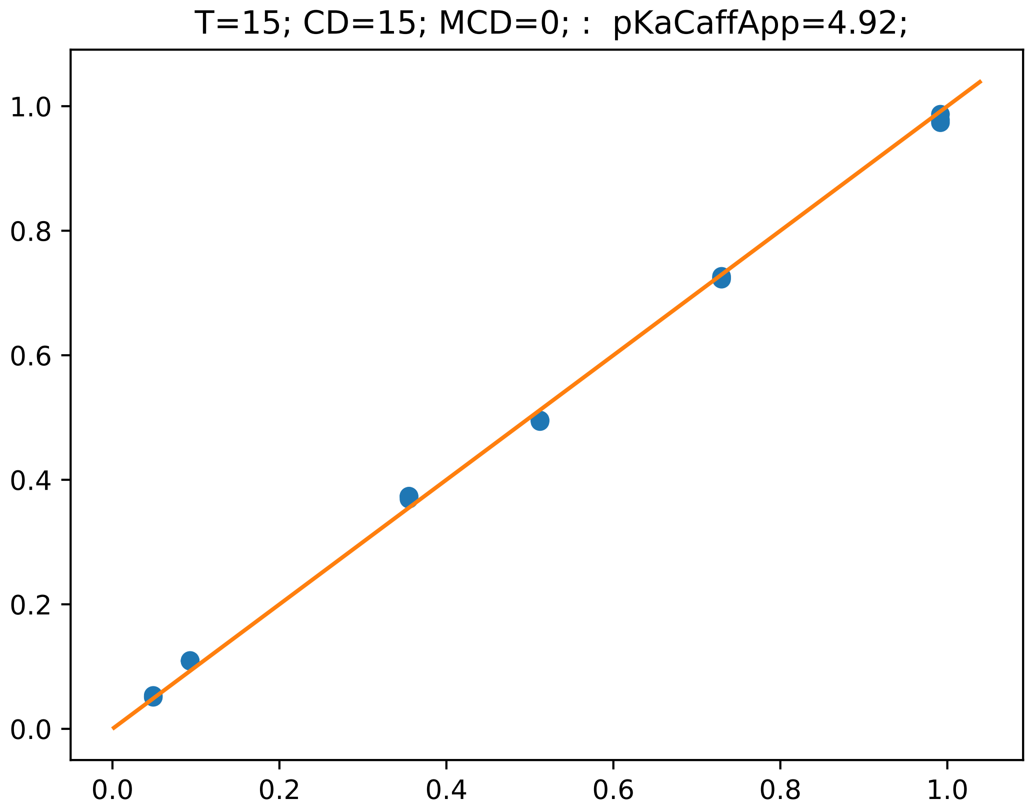
T=15; CD=15; MCD=0; : pKaCaffApp=4.92;
1.0
0.8
0.6
0.4
0.2
0.0
0.0
0.2
0.4
0.6
0.8
1.0

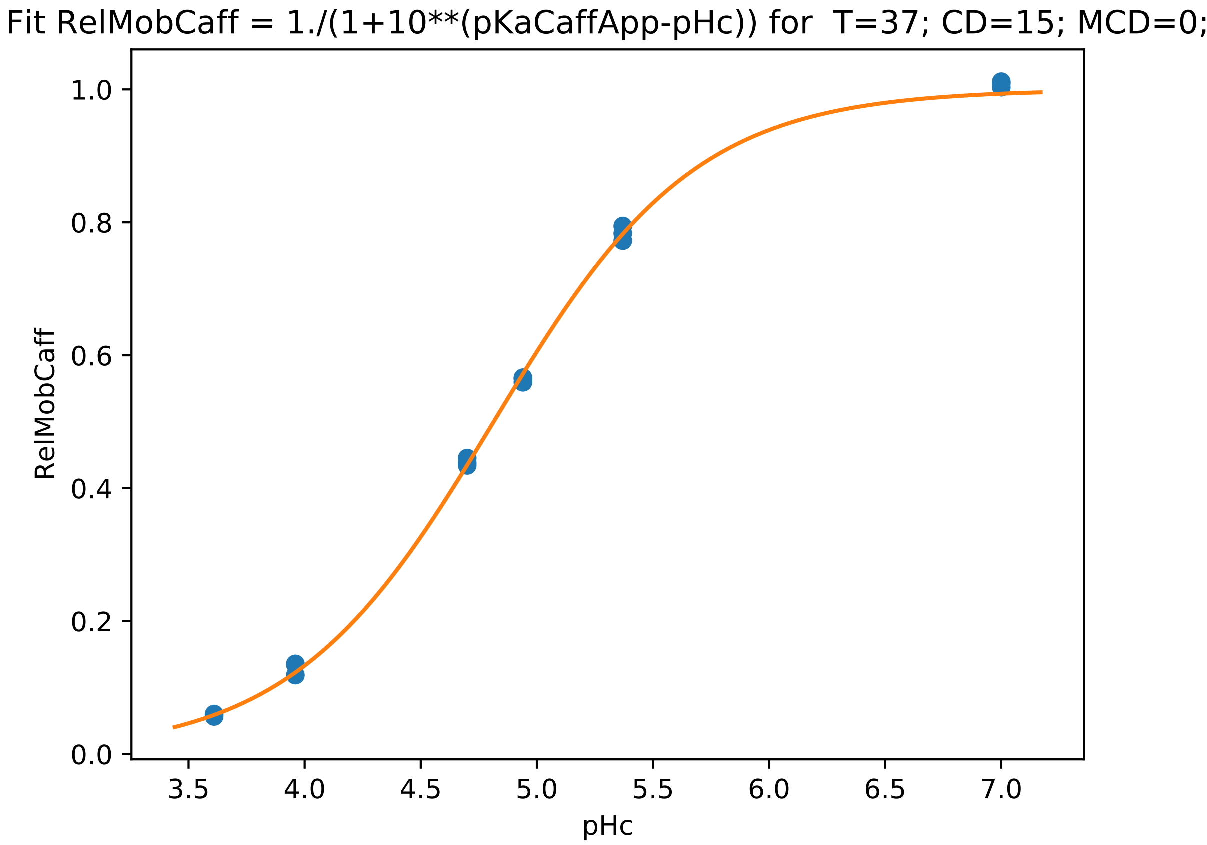

Fit RelMobCaff = 1./(1+10**(pKaCaffApp-pHc)) for T=37; CD=15; MCD=0;
RelMobCaff
pHc
0.0
0.2
0.4
0.6
0.8
1.0
3.5
4.0
4.5
5.0
5.5
6.0
6.5
7.0

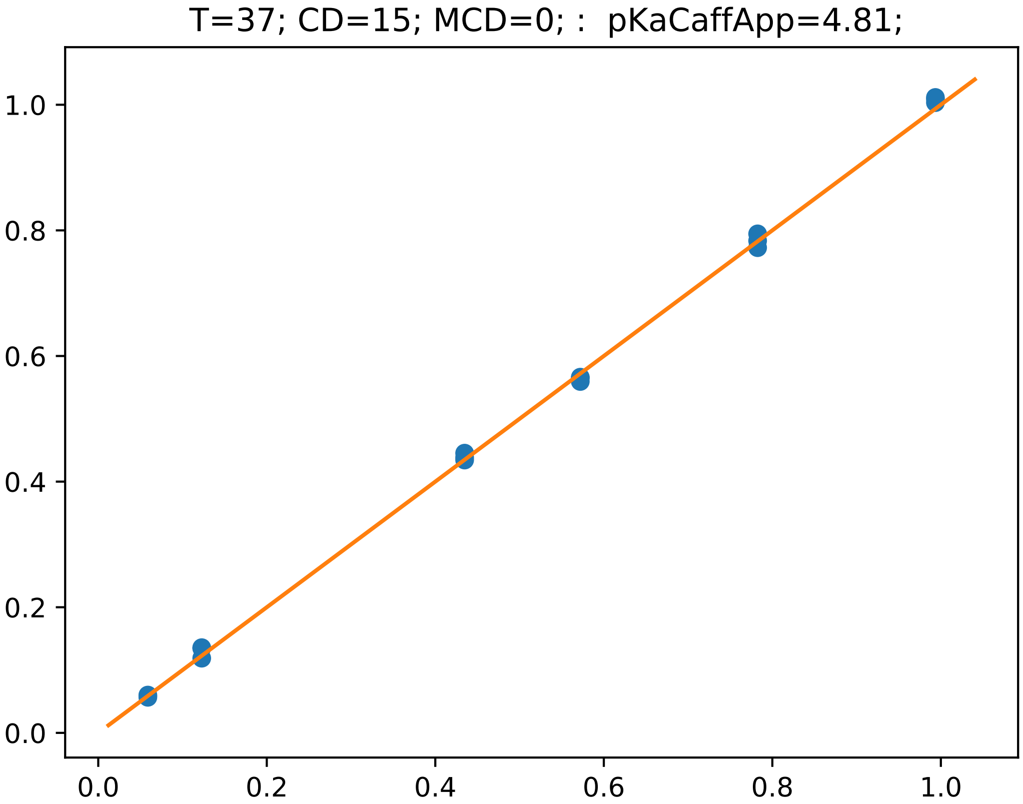
T=37; CD=15; MCD=0; : pKaCaffApp=4.81;
1.0
0.8
0.6
0.4
0.2
0.0
0.0
0.2
0.4
0.6
0.8
1.0

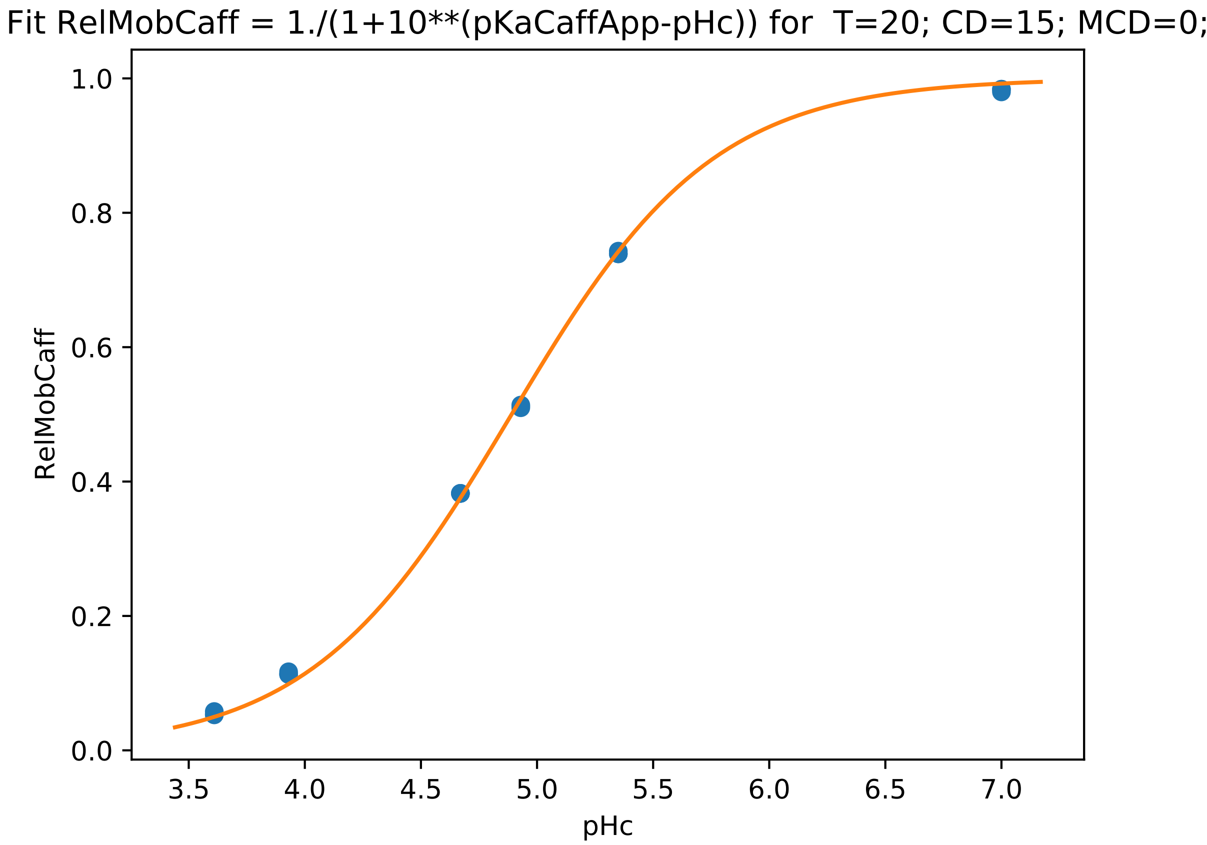

Fit RelMobCaff = 1./(1+10**(pKaCaffApp-pHc)) for T=20; CD=15; MCD=0;
RelMobCaff
pHc
0.0
0.2
0.4
0.6
0.8
1.0
3.5
4.0
4.5
5.0
5.5
6.0
6.5
7.0

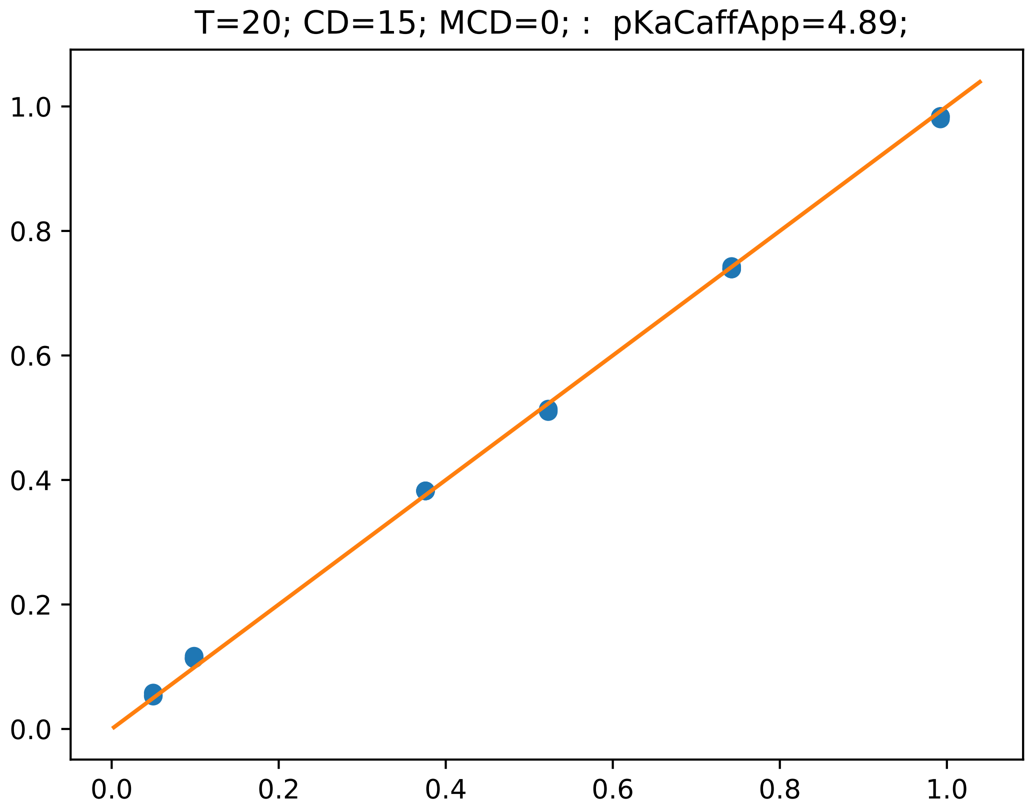
T=20; CD=15; MCD=0; : pKaCaffApp=4.89;
1.0
0.8
0.6
0.4
0.2
0.0
0.0
0.2
0.4
0.6
0.8
1.0

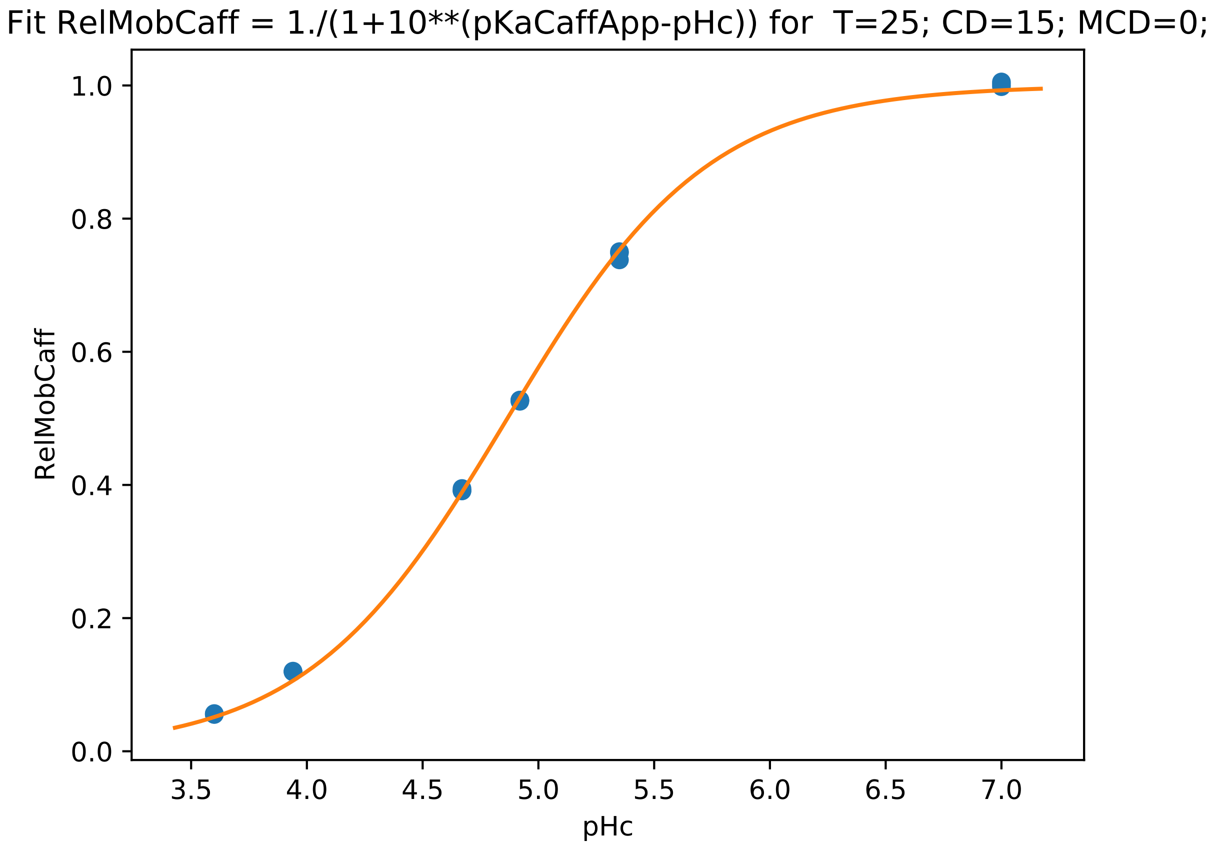

Fit RelMobCaff = 1./(1+10**(pKaCaffApp-pHc)) for T=25; CD=15; MCD=0;
RelMobCaff
pHc
1.0
0.8
0.6
0.4
0.2
0.0
3.5
4.0
4.5
5.0
5.5
6.0
6.5
7.0

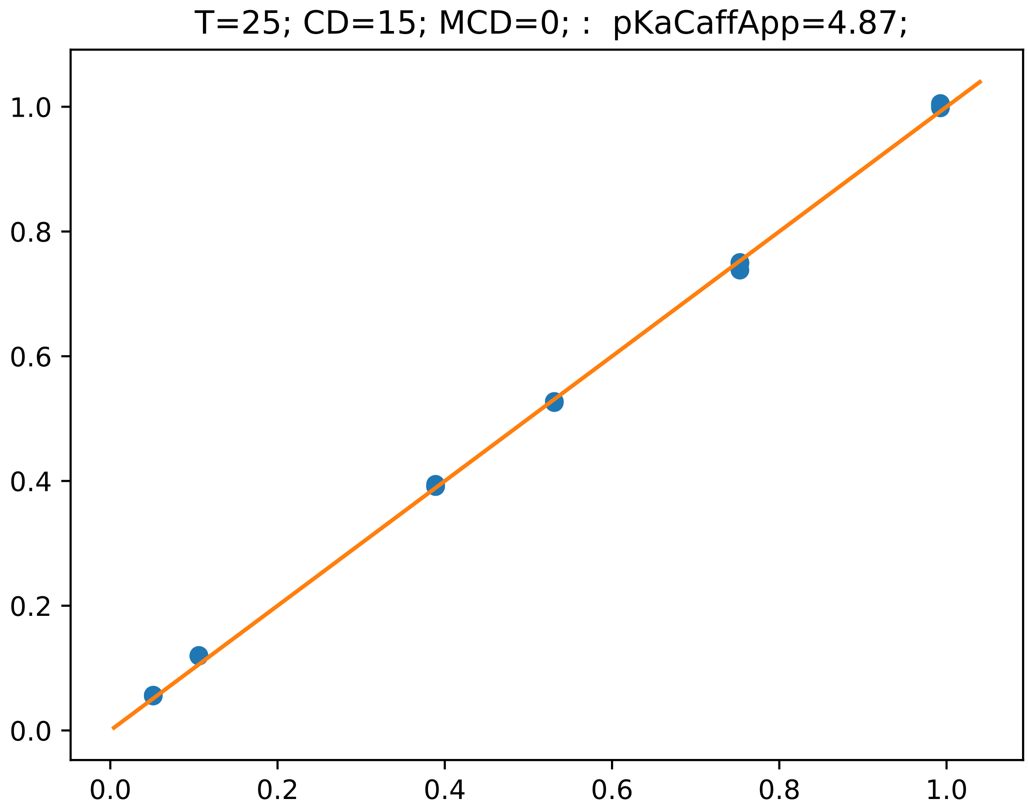

T=25; CD=15; MCD=0; : pKaCaffApp=4.87;
0.0
0.2
0.4
0.6
0.8
1.0
0.0
0.2
0.4
0.6
0.8
1.0

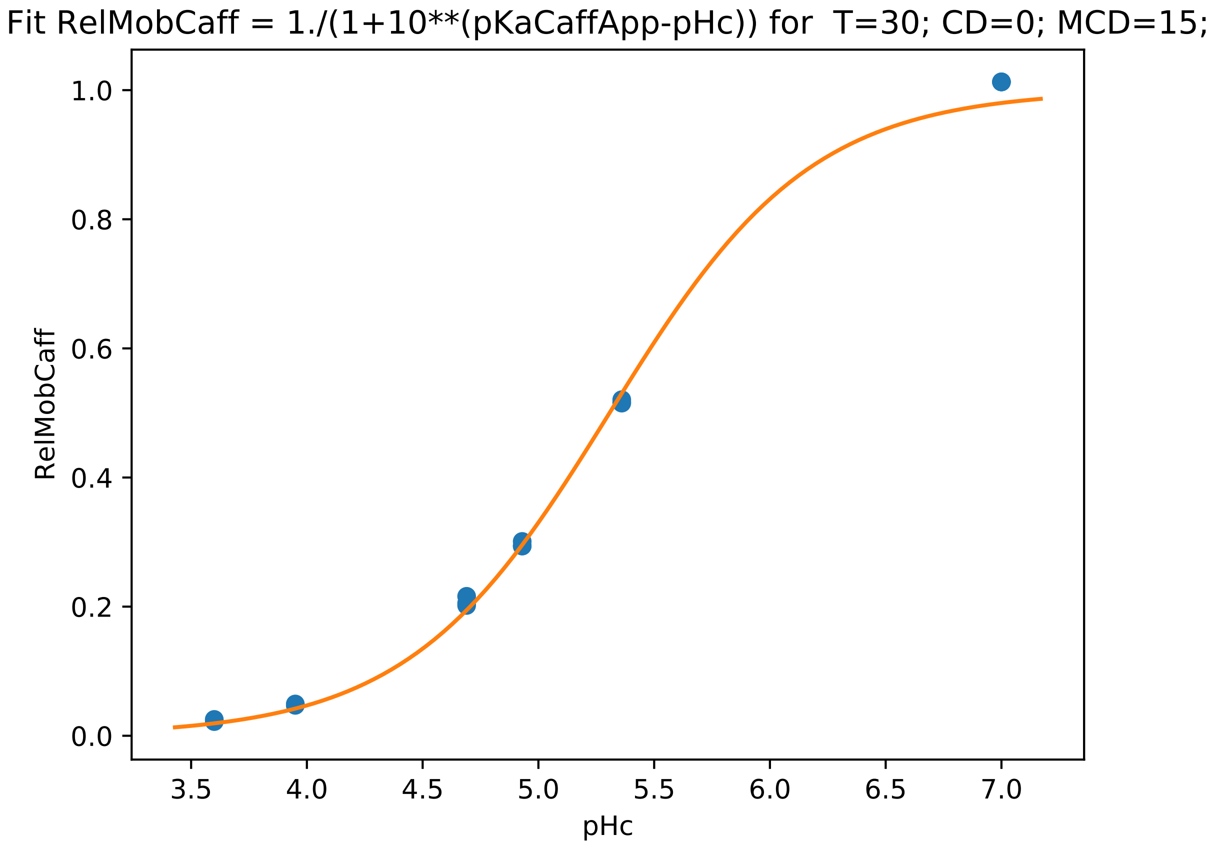

Fit RelMobCaff = 1./(1+10**(pKaCaffApp-pHc)) for T=30; CD=0; MCD=15;
RelMobCaff
pHc
1.0
0.8
0.6
0.4
0.2
0.0
3.5
4.0
4.5
5.0
5.5
6.0
6.5
7.0

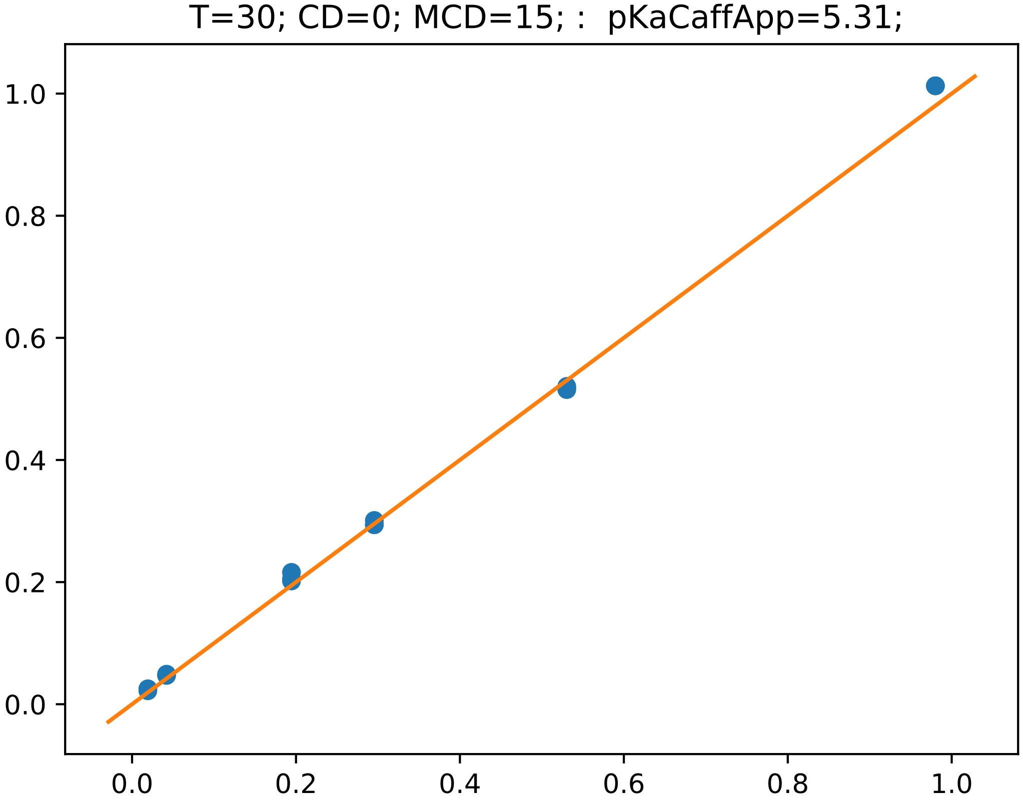
T=30; CD=0; MCD=15; : pKaCaffApp=5.31;
1.0
0.8
0.6
0.4
0.2
0.0
0.0
0.2
0.4
0.6
0.8
1.0

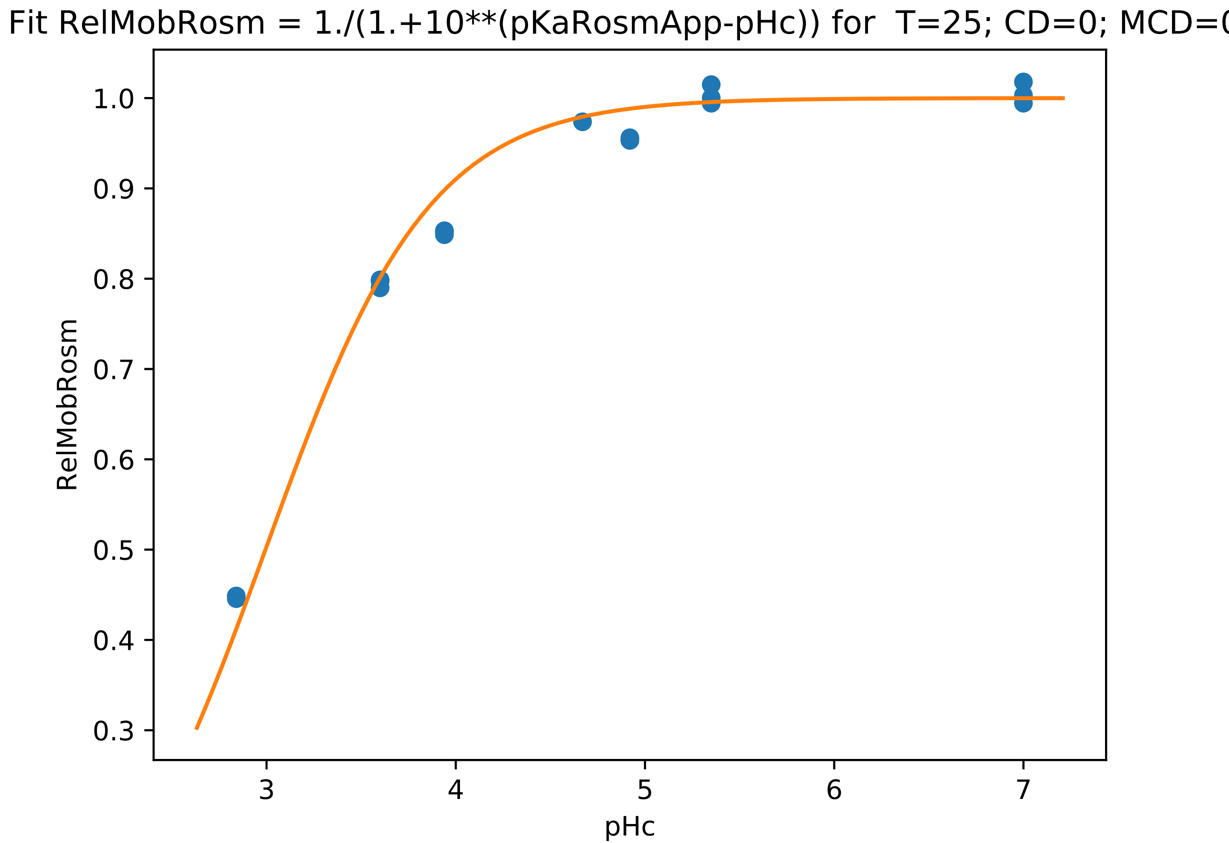

Fit RelMobRosm = 1./(1.+10**(pKaRosmApp-pHc)) for T=25; CD=0; MCD=0
1.0
0.9
0.8
0.7
0.6
0.5
0.4
0.3
RelMobRosm
3
4
5
6
7
pHc

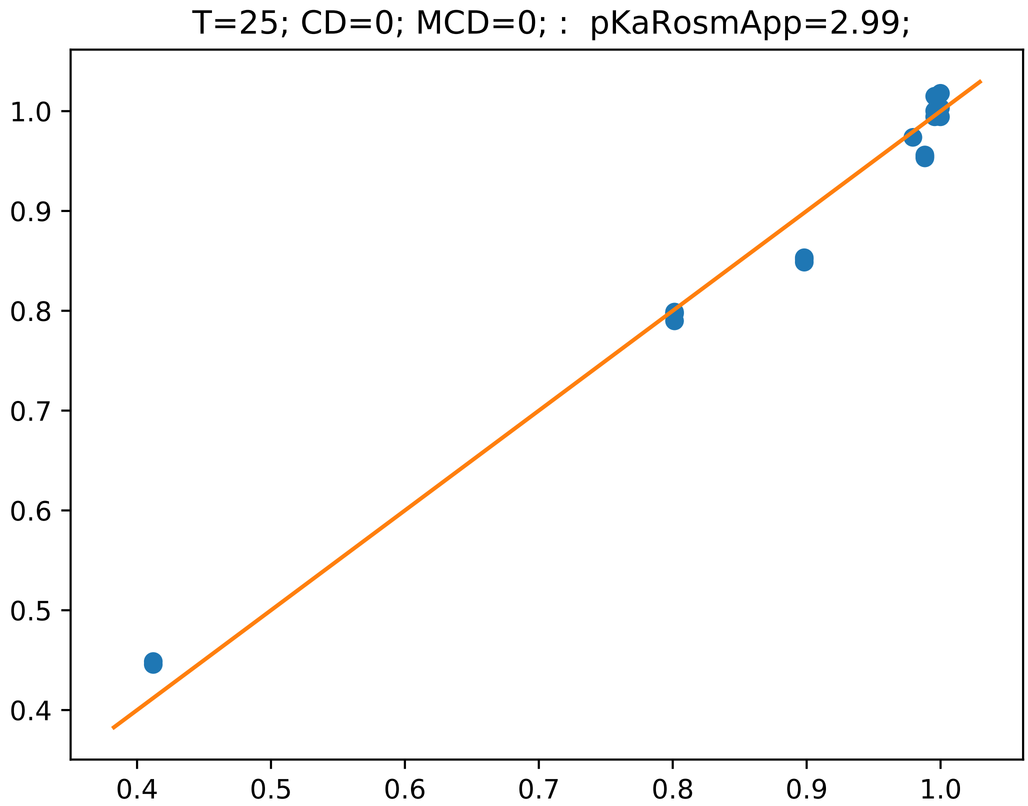
T=25; CD=0; MCD=0; : pKaRosmApp=2.99;
1.0
0.9
0.8
0.7
0.6
0.5
0.4
0.4
0.5
0.6
0.7
0.8
0.9
1.0

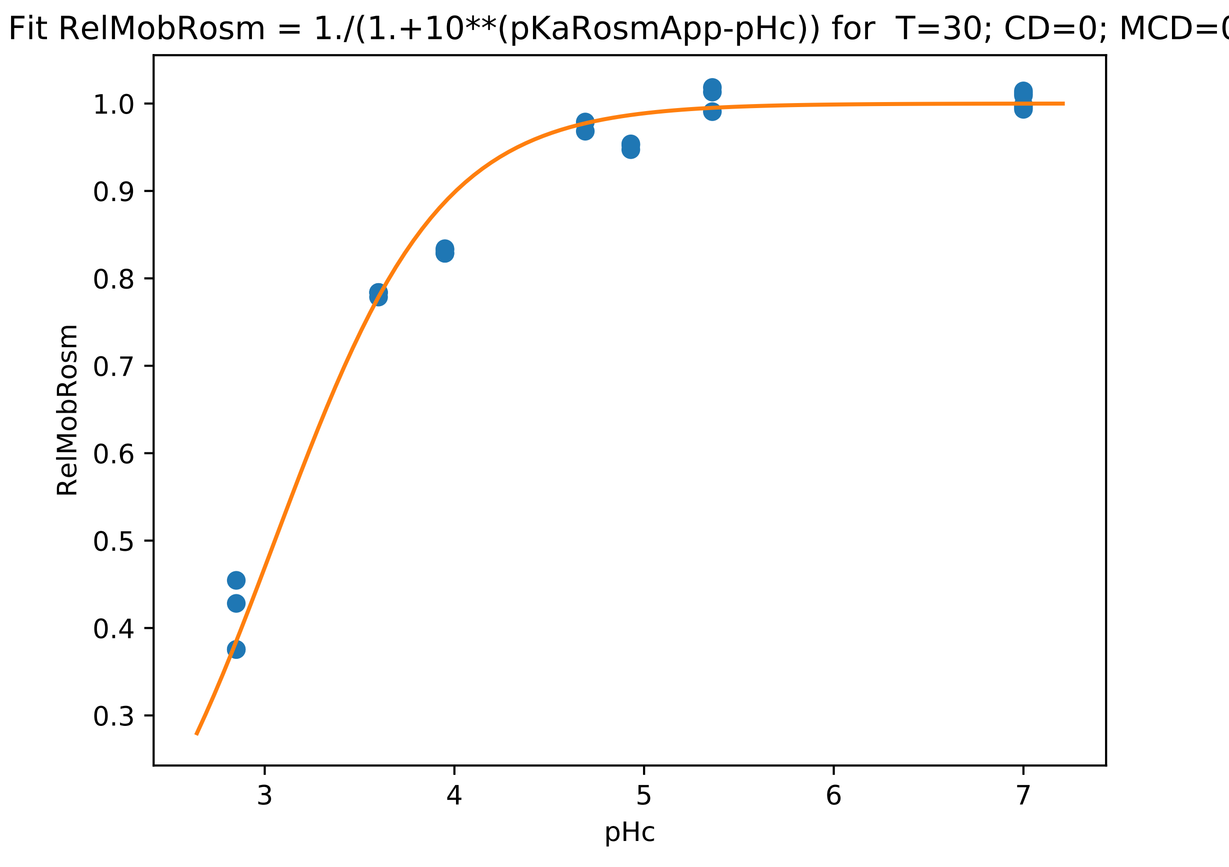
Fit RelMobRosm = 1./(1.+10**(pKaRosmApp-pHc)) for T=30; CD=0; MCD=0
RelMobRosm
1.0
0.9
0.8
0.7
0.6
0.5
0.4
0.3
3
4
5
6
7
pHc

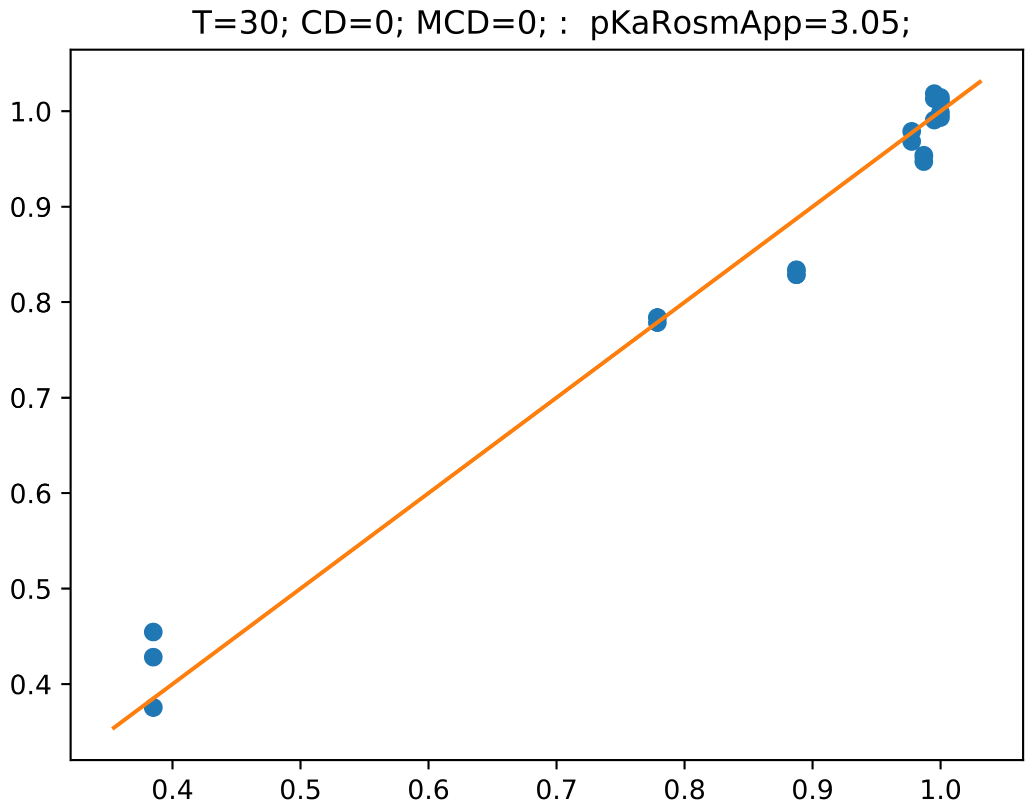
T=30; CD=0; MCD=0; : pKaRosmApp=3.05;
1.0
0.9
0.8
0.7
0.6
0.5
0.4
0.4
0.5
0.6
0.7
0.8
0.9
1.0

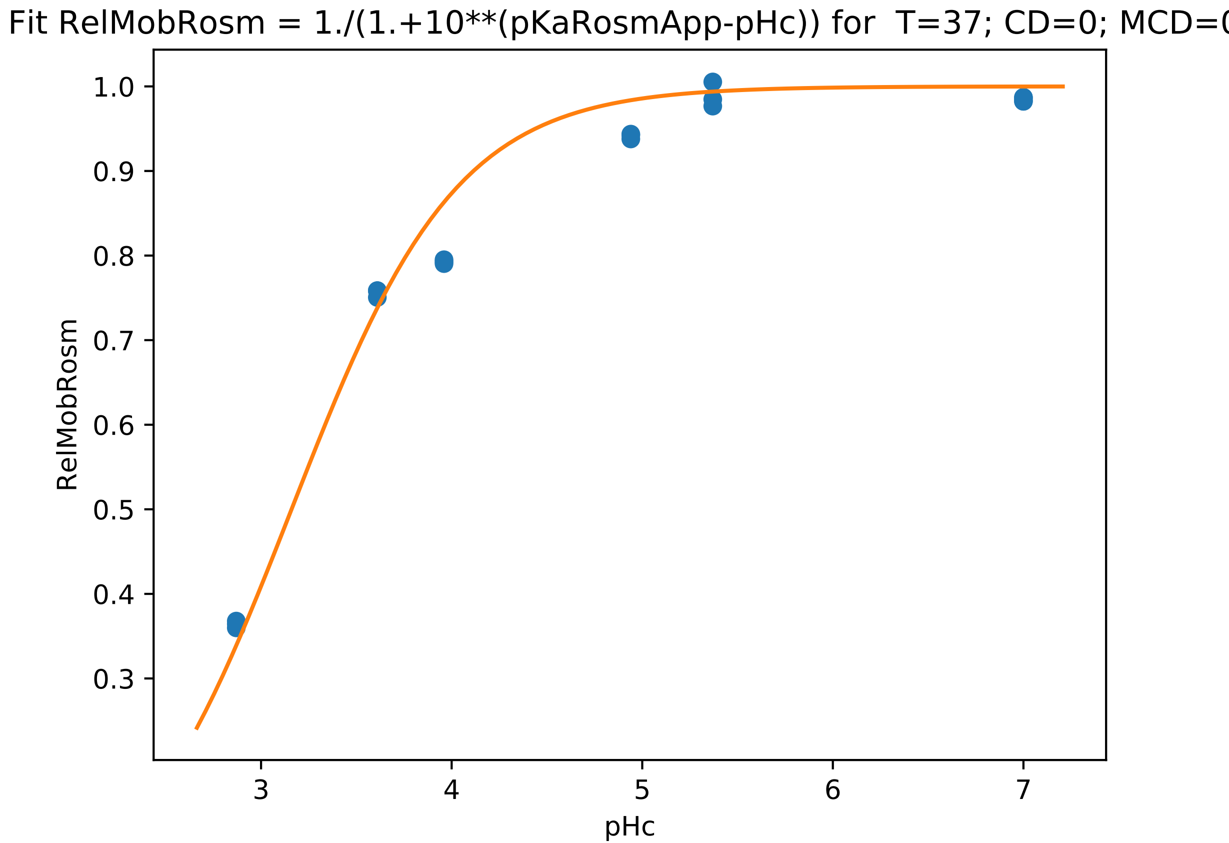
Fit RelMobRosm = 1./(1.+10**(pKaRosmApp-pHc)) for T=37; CD=0; MCD=0
RelMobRosm
pHc
1.0
0.9
0.8
0.7
0.6
0.5
0.4
0.3
3
4
5
6
7

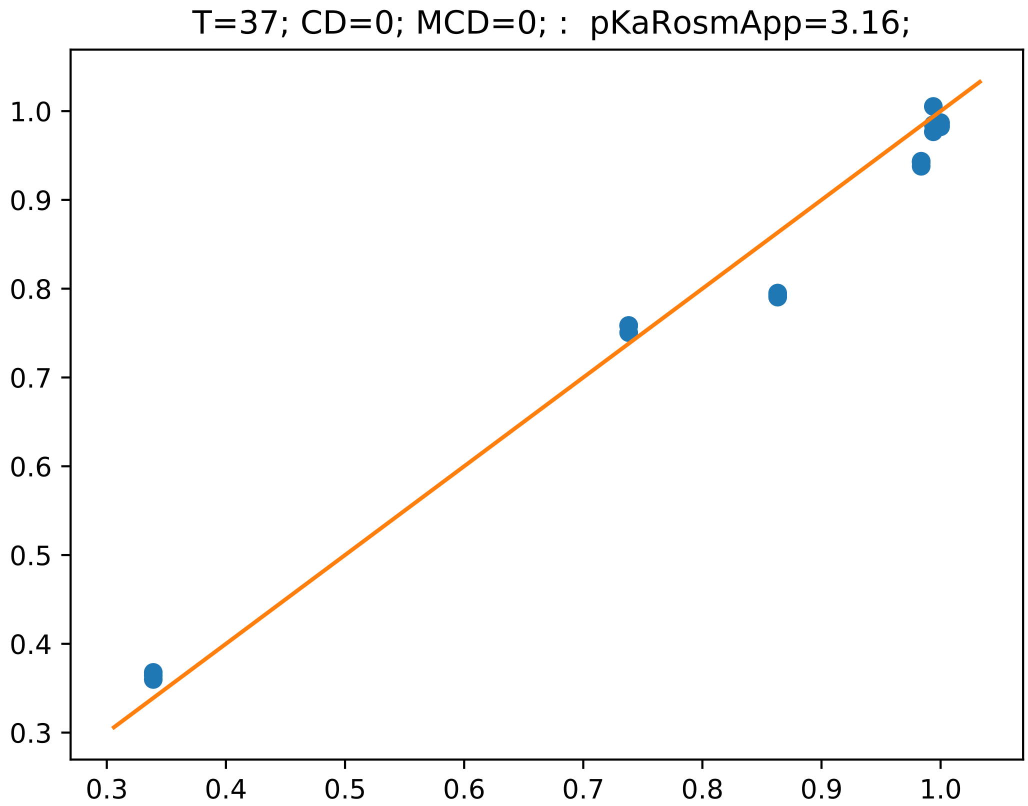

T=37; CD=0; MCD=0; : pKaRosmApp=3.16;
1.0
0.9
0.8
0.7
0.6
0.5
0.4
0.3
0.3
0.4
0.5
0.6
0.7
0.8
0.9
1.0

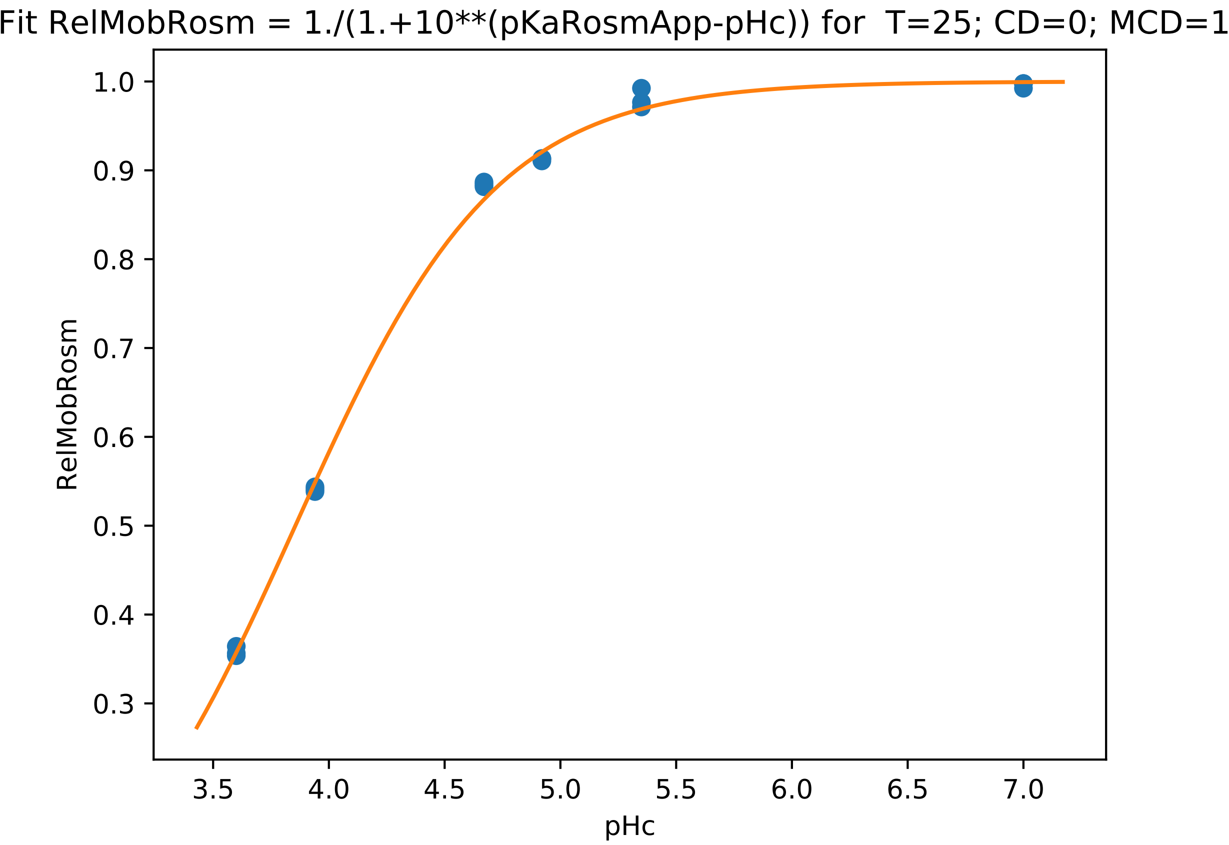
Fit RelMobRosm = 1./(1.+10**(pKaRosmApp-pHc)) for T=25; CD=0; MCD=1
RelMobRosm
pHc
1.0
0.9
0.8
0.7
0.6
0.5
0.4
0.3
3.5
4.0
4.5
5.0
5.5
6.0
6.5
7.0

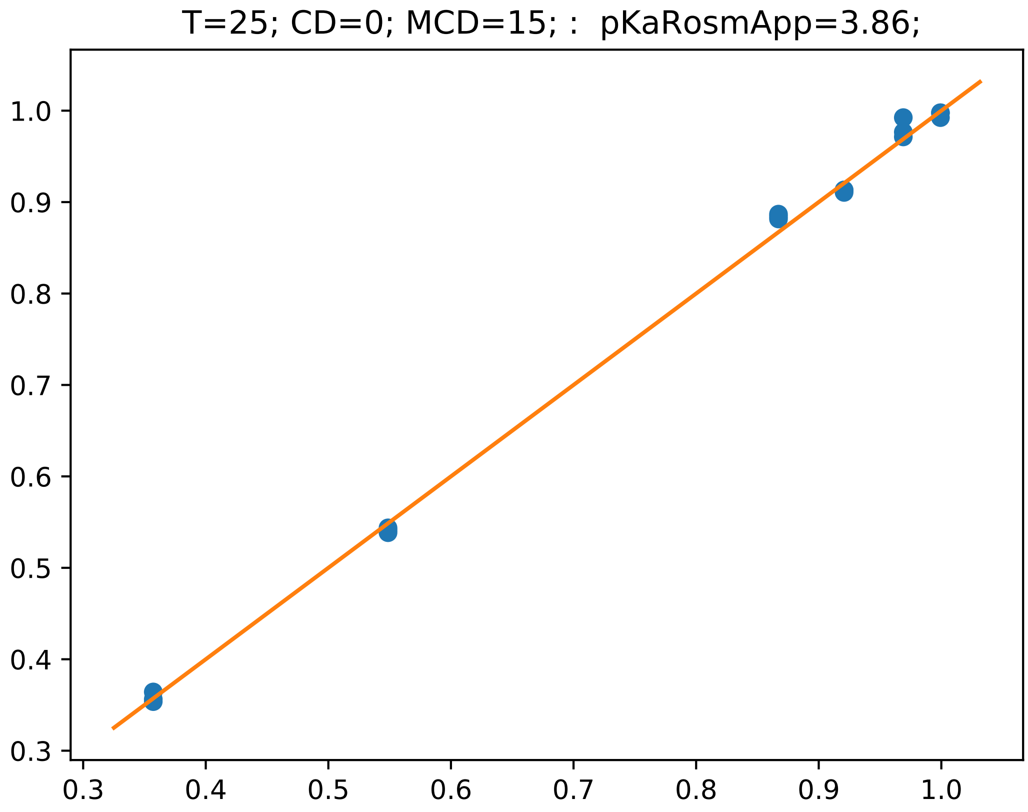
T=25; CD=0; MCD=15; : pKaRosmApp=3.86;
1.0
0.9
0.8
0.7
0.6
0.5
0.4
0.3
0.3
0.4
0.5
0.6
0.7
0.8
0.9
1.0

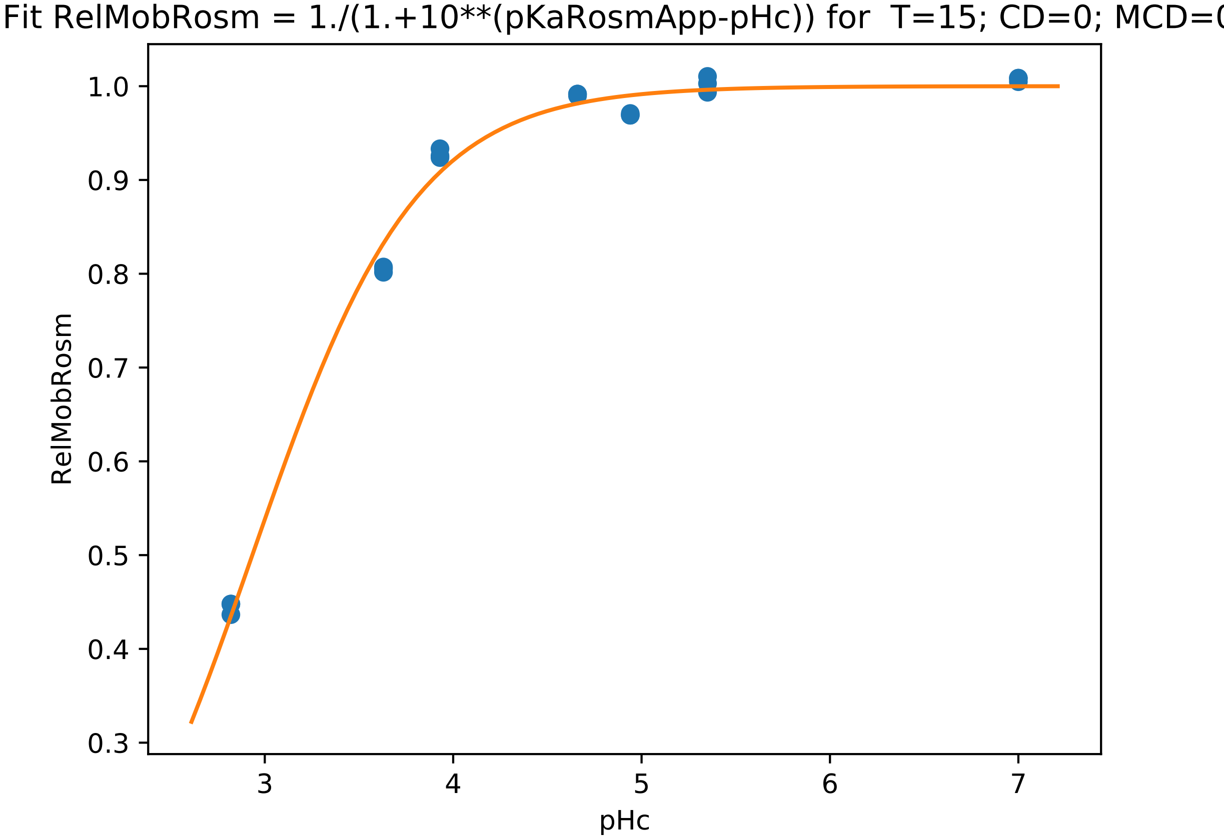
Fit RelMobRosm = 1./(1.+10**(pKaRosmApp-pHc)) for T=15; CD=0; MCD=0
RelMobRosm
1.0
0.9
0.8
0.7
0.6
0.5
0.4
0.3
3
4
5
6
7
pHc

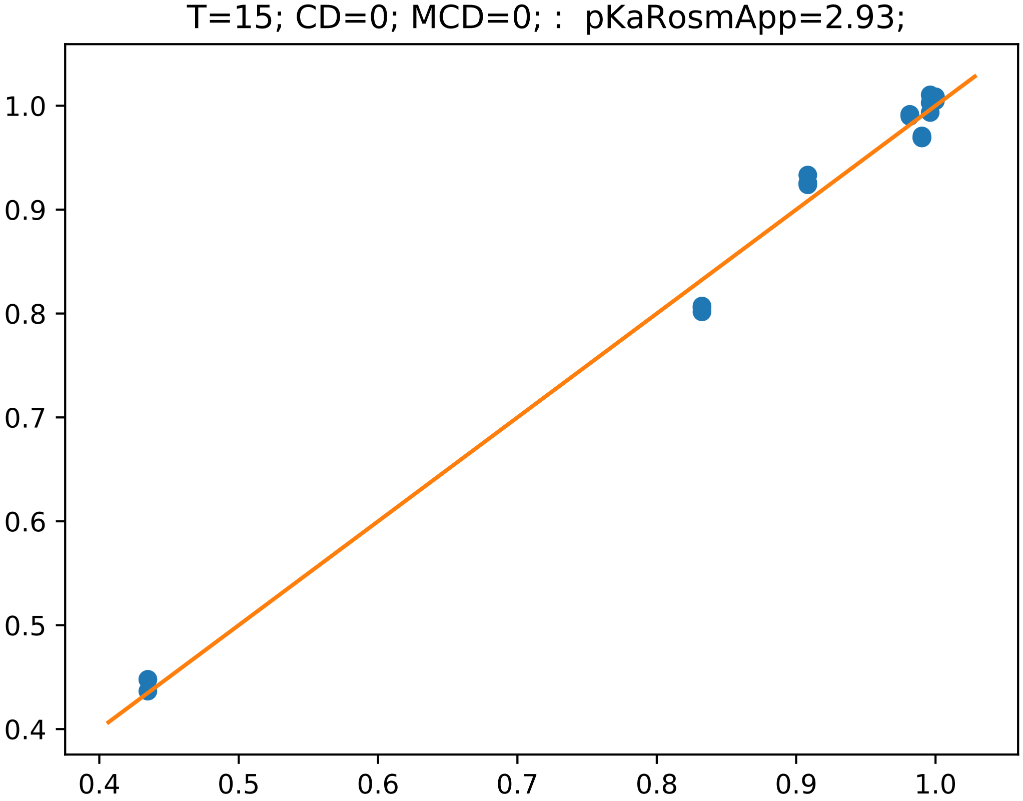

T=15; CD=0; MCD=0; : pKaRosmApp=2.93;
1.0
0.9
0.8
0.7
0.6
0.5
0.4
0.4
0.5
0.6
0.7
0.8
0.9
1.0

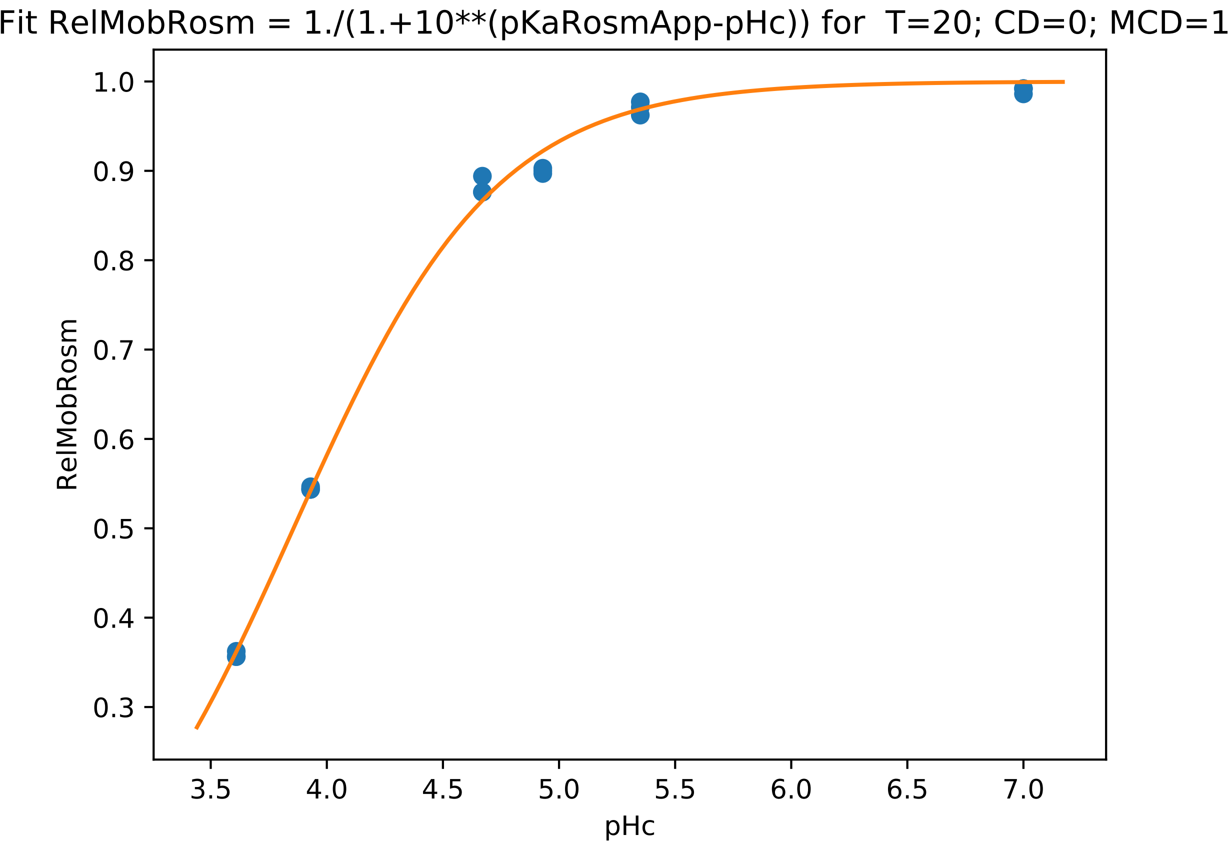

Fit RelMobRosm = 1./(1.+10**(pKaRosmApp-pHc)) for T=20; CD=0; MCD=1
RelMobRosm
pHc
1.0
0.9
0.8
0.7
0.6
0.5
0.4
0.3
3.5
4.0
4.5
5.0
5.5
6.0
6.5
7.0

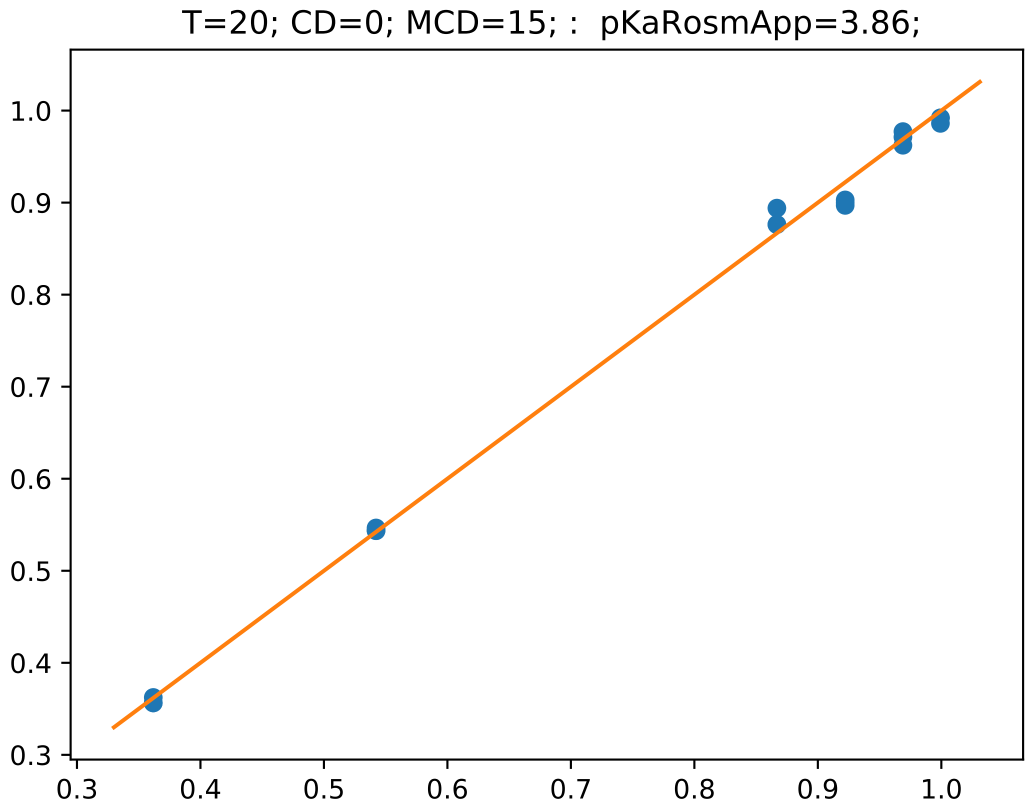
T=20; CD=0; MCD=15; : pKaRosmApp=3.86;
1.0
0.9
0.8
0.7
0.6
0.5
0.4
0.3
0.3
0.4
0.5
0.6
0.7
0.8
0.9
1.0

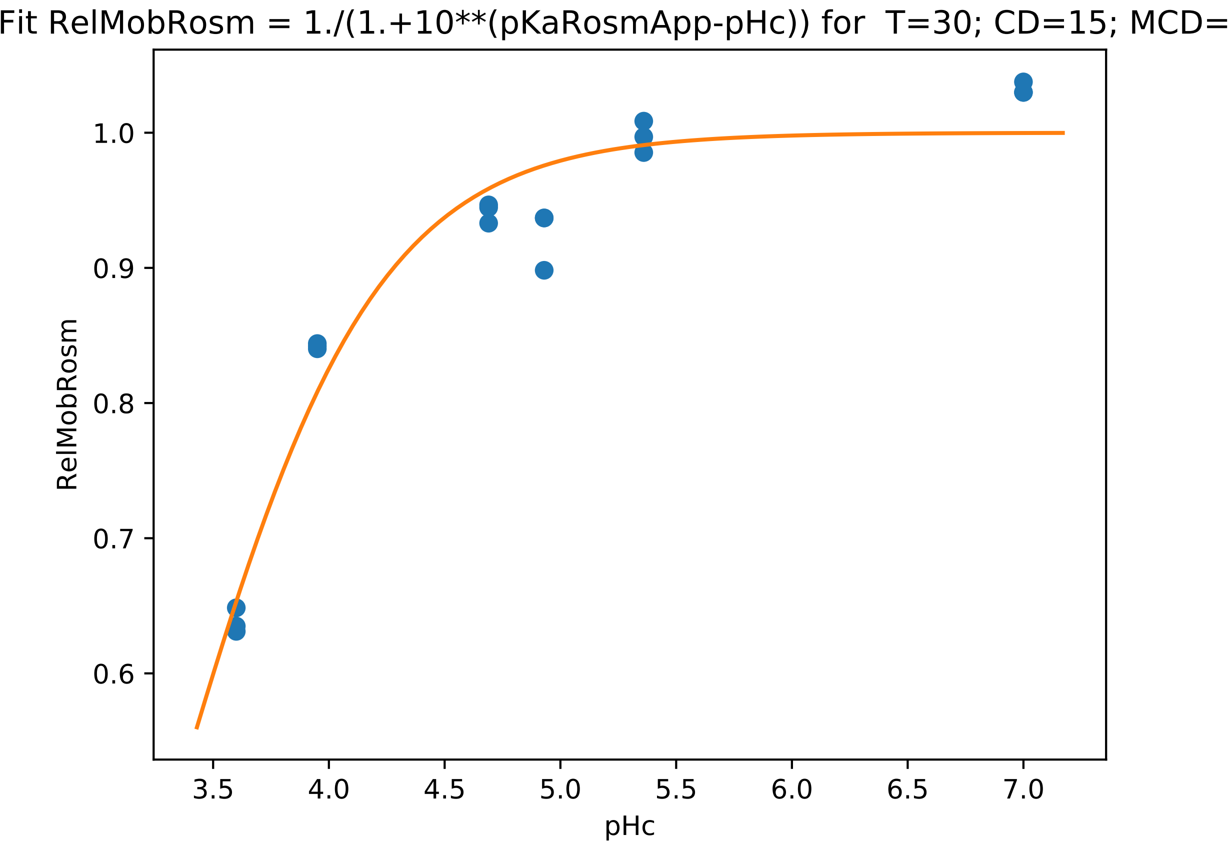

Fit RelMobRosm = 1./(1.+10**(pKaRosmApp-pHc)) for T=30; CD=15; MCD=
RelMobRosm
pHc
1.0
0.9
0.8
0.7
0.6
3.5
4.0
4.5
5.0
5.5
6.0
6.5
7.0

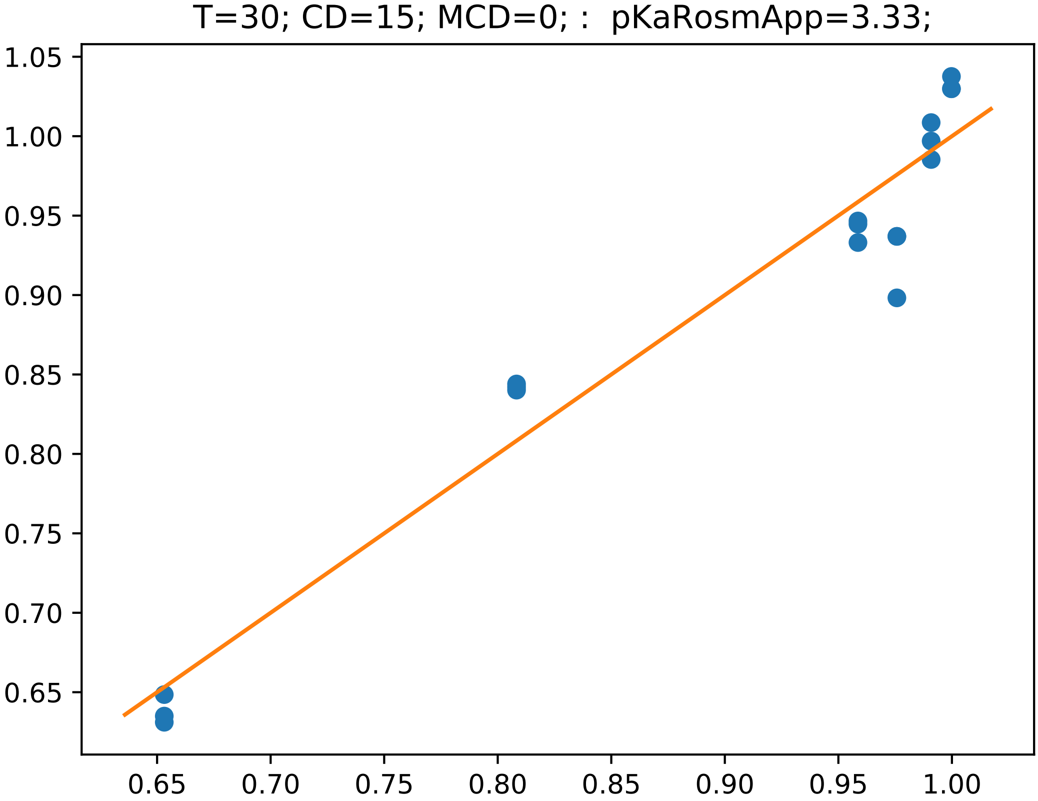
T=30; CD=15; MCD=0; : pKaRosmApp=3.33;
1.05
1.00
0.95
0.90
0.85
0.80
0.75
0.70
0.65
0.65
0.70
0.75
0.80
0.85
0.90
0.95
1.00

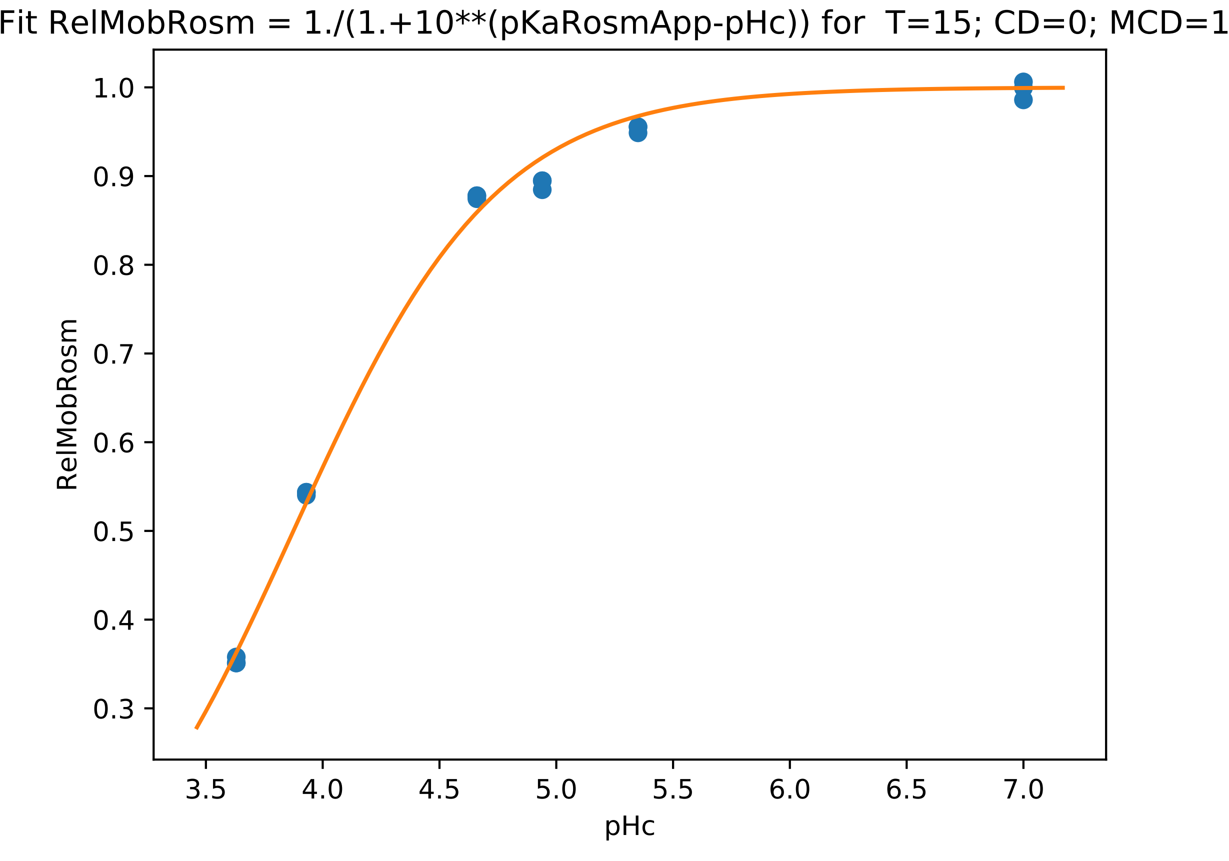

Fit RelMobRosm = 1./(1.+10**(pKaRosmApp-pHc)) for T=15; CD=0; MCD=1
RelMobRosm
pHc
1.0
0.9
0.8
0.7
0.6
0.5
0.4
0.3
3.5
4.0
4.5
5.0
5.5
6.0
6.5
7.0

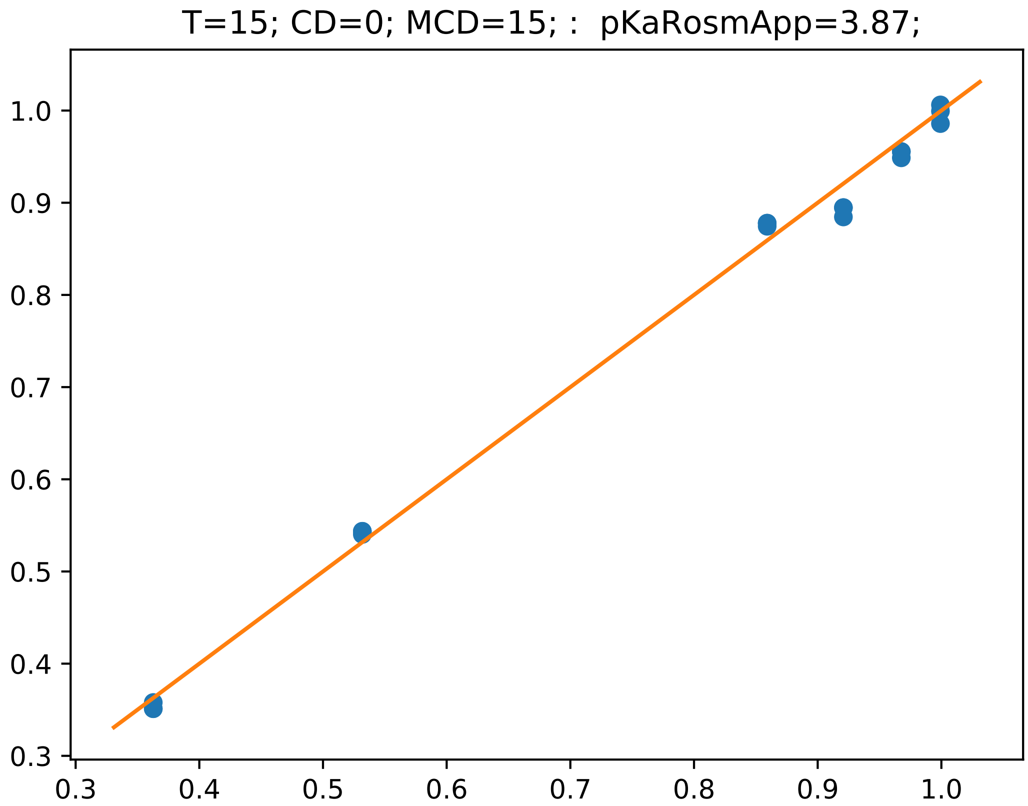

T=15; CD=0; MCD=15; : pKaRosmApp=3.87;
1.0
0.9
0.8
0.7
0.6
0.5
0.4
0.3
0.3
0.4
0.5
0.6
0.7
0.8
0.9
1.0

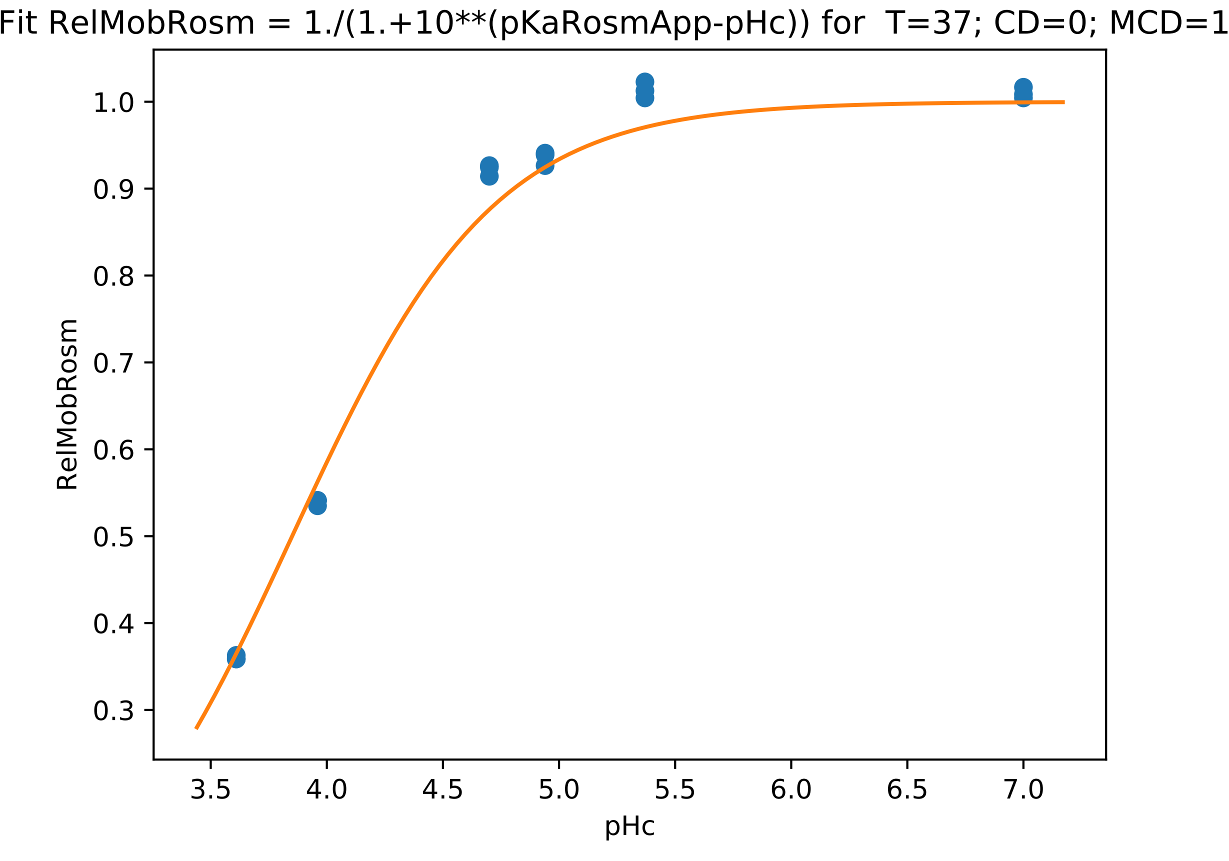
Fit RelMobRosm = 1./(1.+10**(pKaRosmApp-pHc)) for T=37; CD=0; MCD=1
RelMobRosm
pHc
1.0
0.9
0.8
0.7
0.6
0.5
0.4
0.3
3.5
4.0
4.5
5.0
5.5
6.0
6.5
7.0

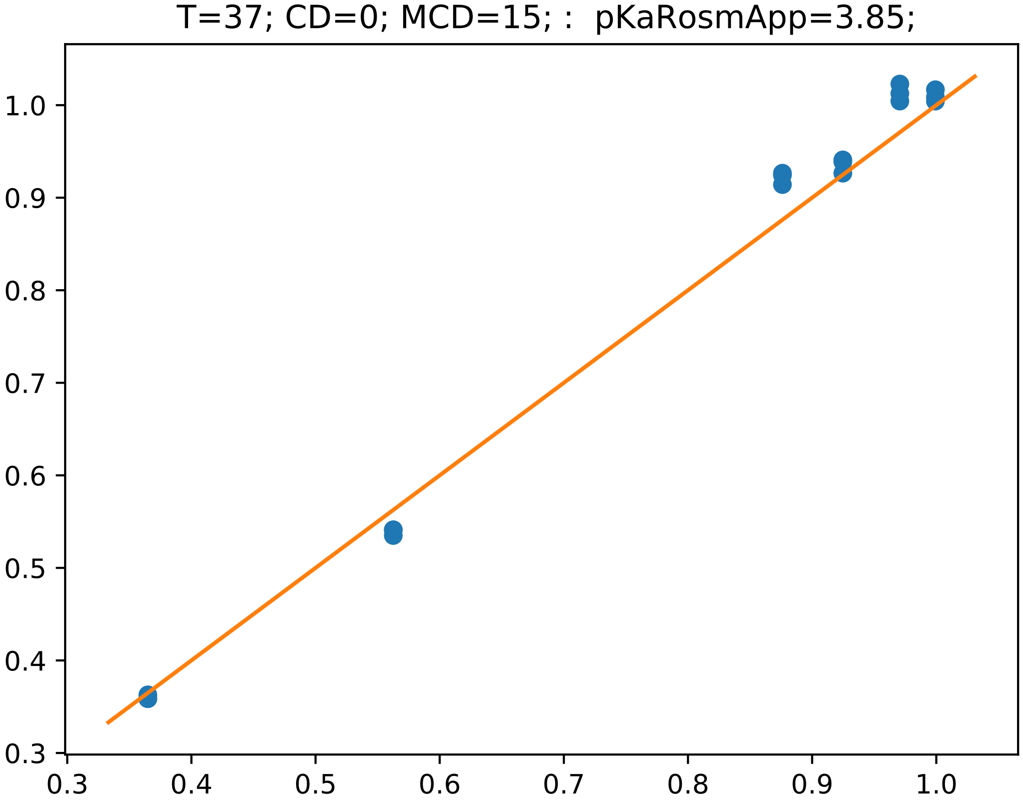
T=37; CD=0; MCD=15; : pKaRosmApp=3.85;
1.0
0.9
0.8
0.7
0.6
0.5
0.4
0.3
0.3
0.4
0.5
0.6
0.7
0.8
0.9
1.0

Fit RelMobRosm = 1./(1.+10**(pKaRosmApp-pHc)) for T=20; CD=0; MCD=0

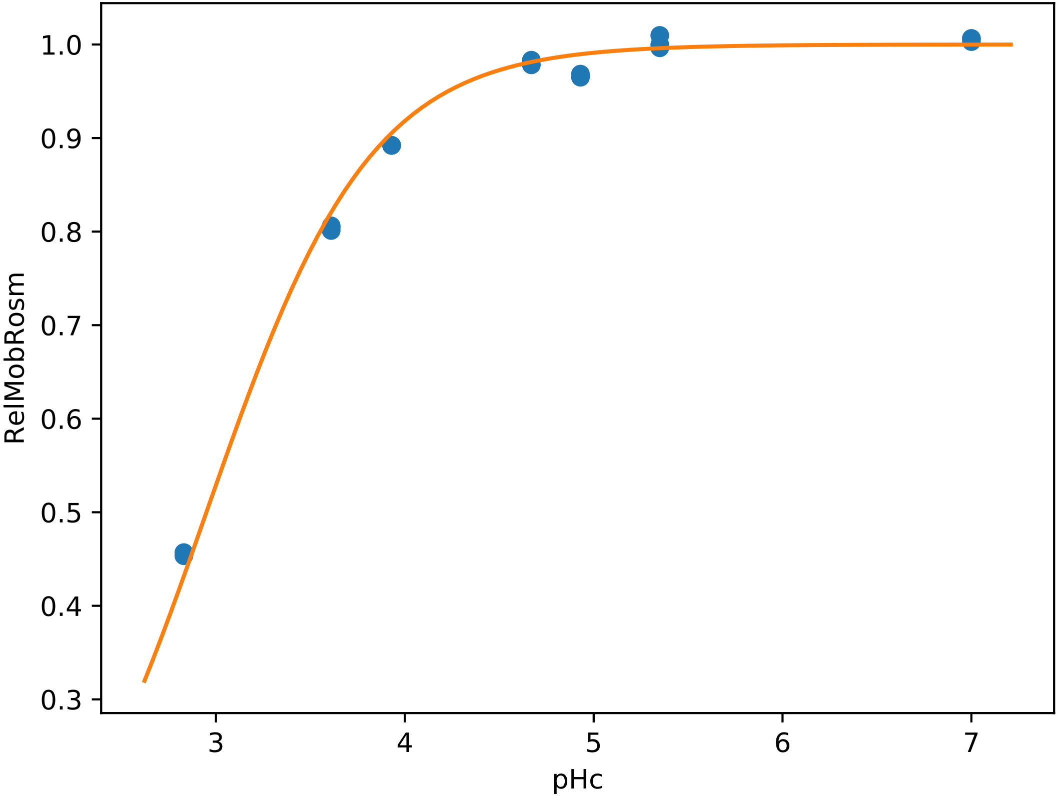

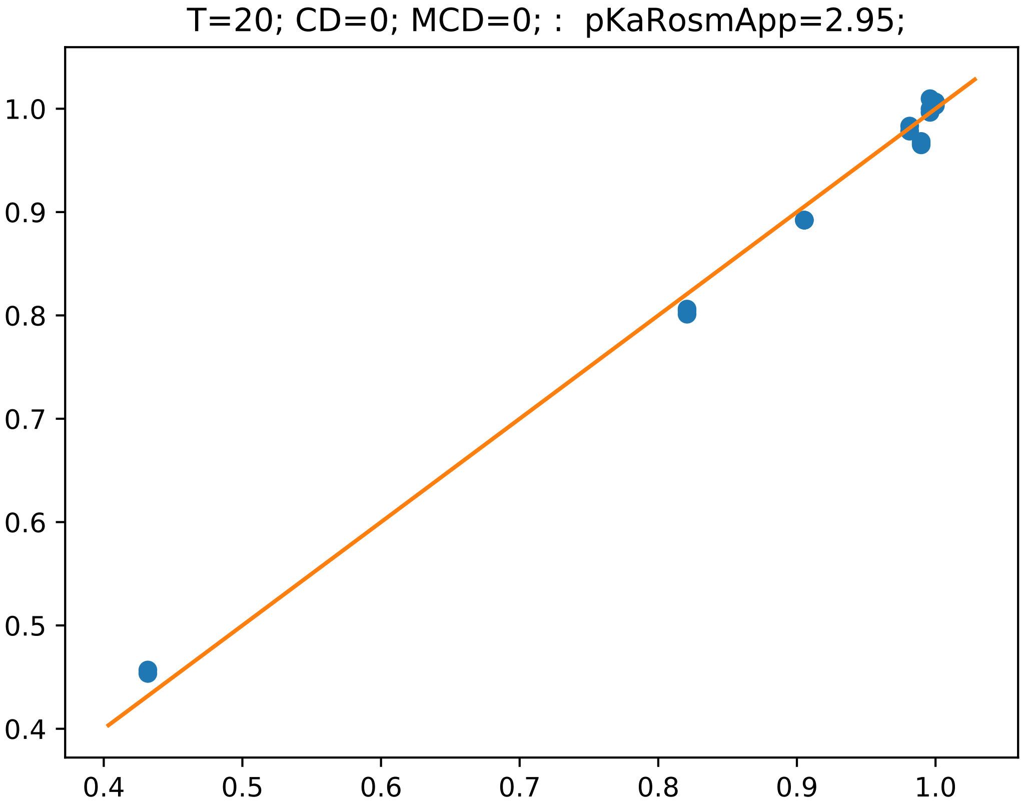

T=20; CD=0; MCD=0; : pKaRosmApp=2.95;
1.0
0.9
0.8
0.7
0.6
0.5
0.4
0.4
0.5
0.6
0.7
0.8
0.9
1.0

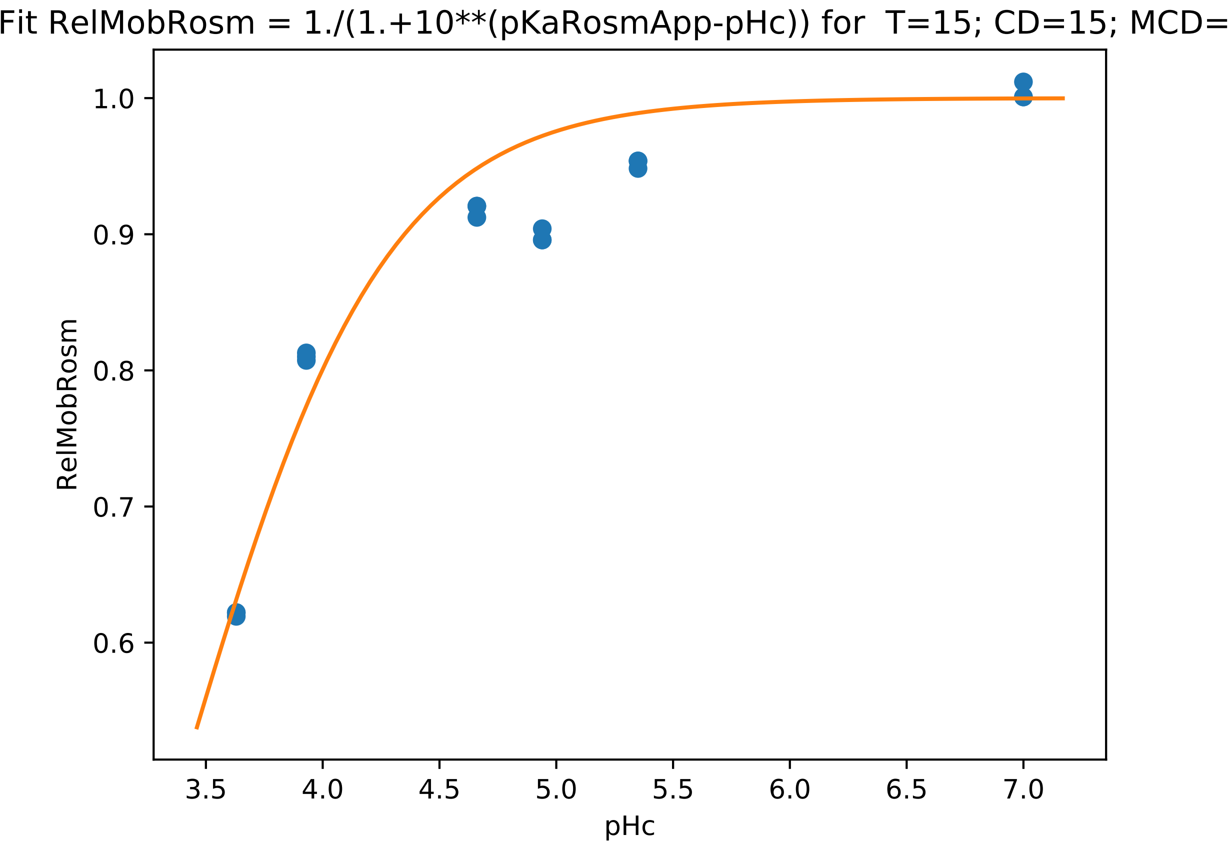

Fit RelMobRosm = 1./(1.+10**(pKaRosmApp-pHc)) for T=15; CD=15; MCD=
RelMobRosm
pHc
1.0
0.9
0.8
0.7
0.6
3.5
4.0
4.5
5.0
5.5
6.0
6.5
7.0

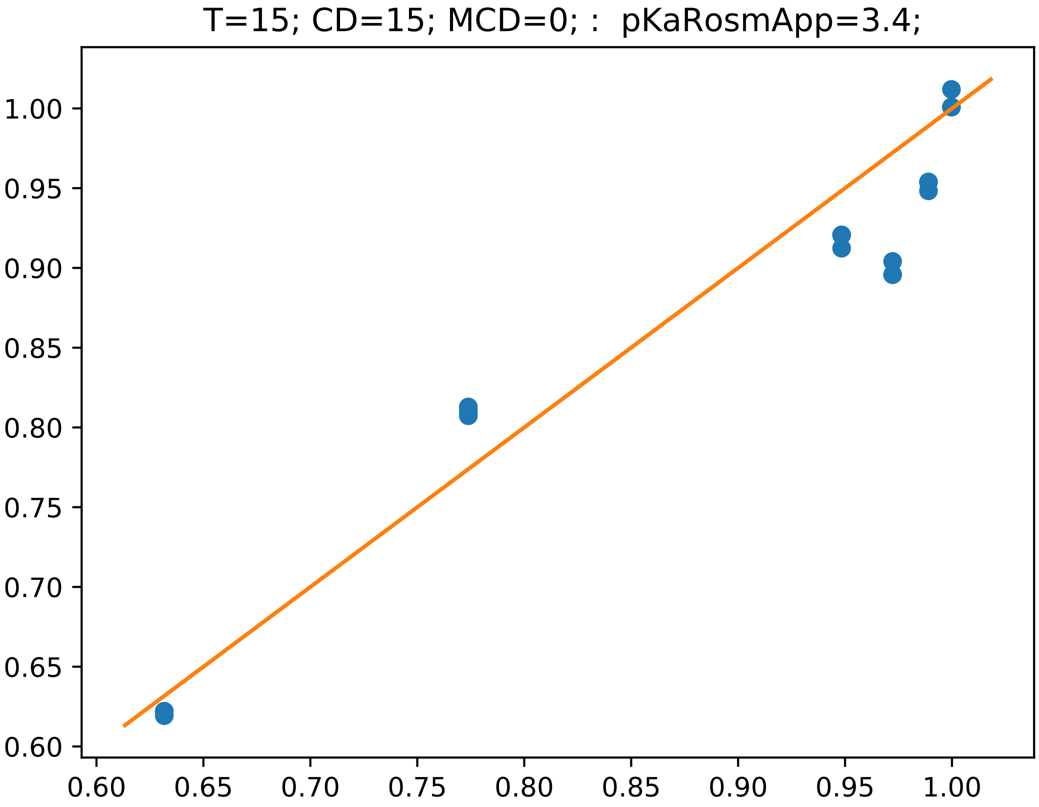
T=15; CD=15; MCD=0; : pKaRosmApp=3.4;
1.00
0.95
0.90
0.85
0.80
0.75
0.70
0.65
0.60
0.60
0.65
0.70
0.75
0.80
0.85
0.90
0.95
1.00

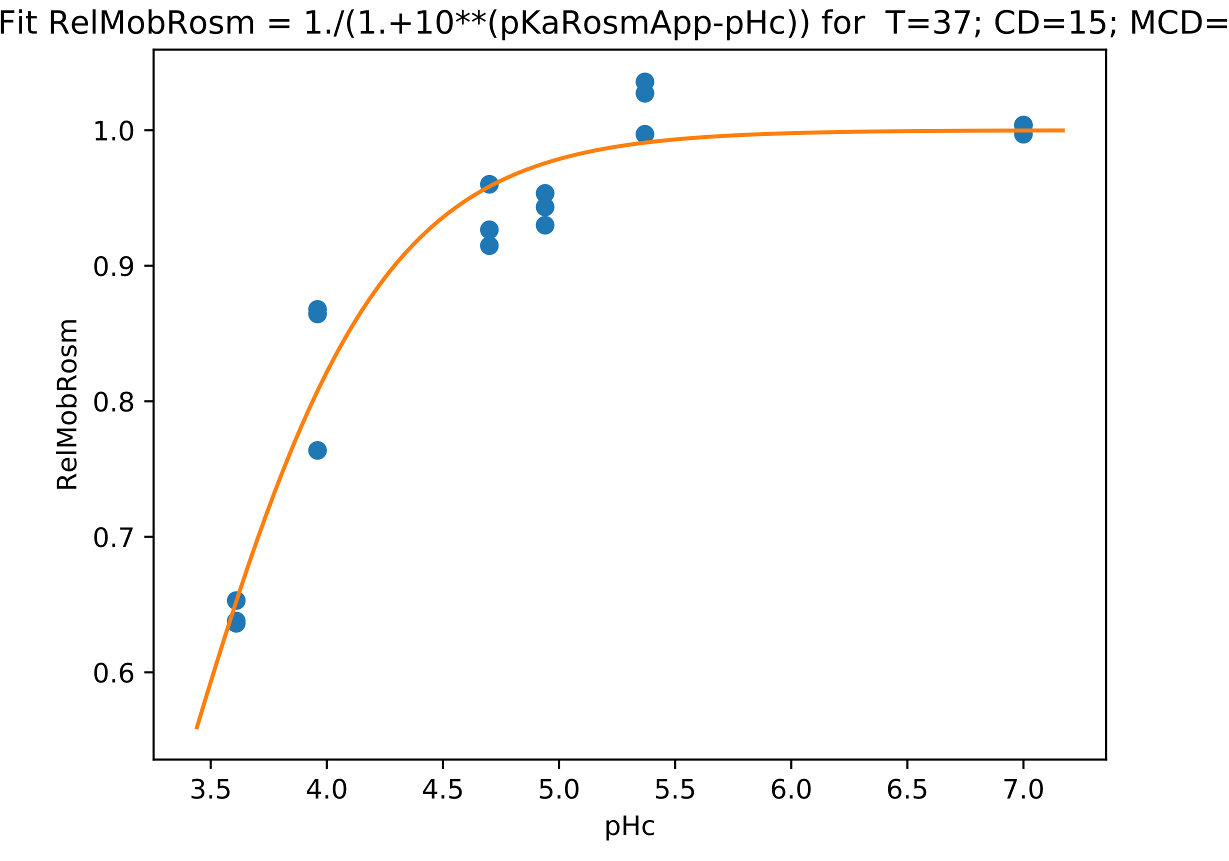

Fit RelMobRosm = 1./(1.+10**(pKaRosmApp-pHc)) for T=37; CD=15; MCD=
RelMobRosm
pHc
1.0
0.9
0.8
0.7
0.6
3.5
4.0
4.5
5.0
5.5
6.0
6.5
7.0

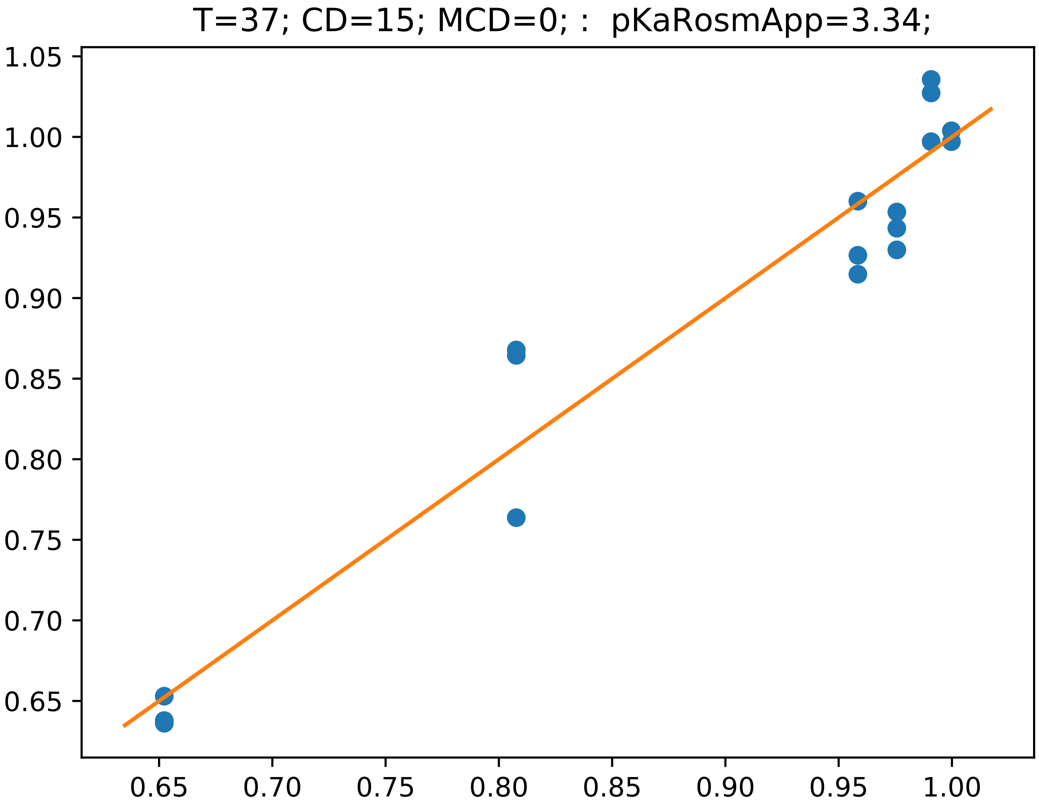
T=37; CD=15; MCD=0; : pKaRosmApp=3.34;
1.05
1.00
0.95
0.90
0.85
0.80
0.75
0.70
0.65
0.65
0.70
0.75
0.80
0.85
0.90
0.95
1.00

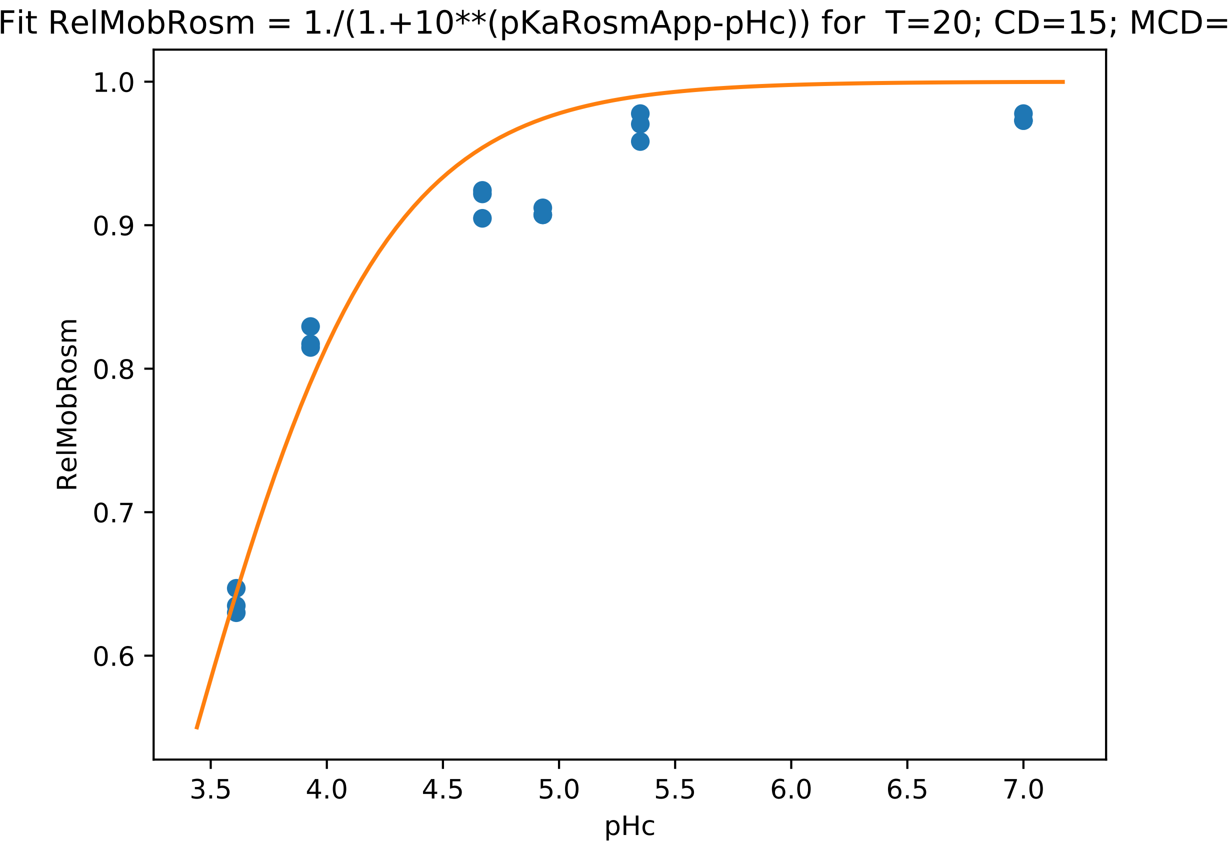
Fit RelMobRosm = 1./(1.+10**(pKaRosmApp-pHc)) for T=20; CD=15; MCD=
RelMobRosm
pHc
1.0
0.9
0.8
0.7
0.6
3.5
4.0
4.5
5.0
5.5
6.0
6.5
7.0

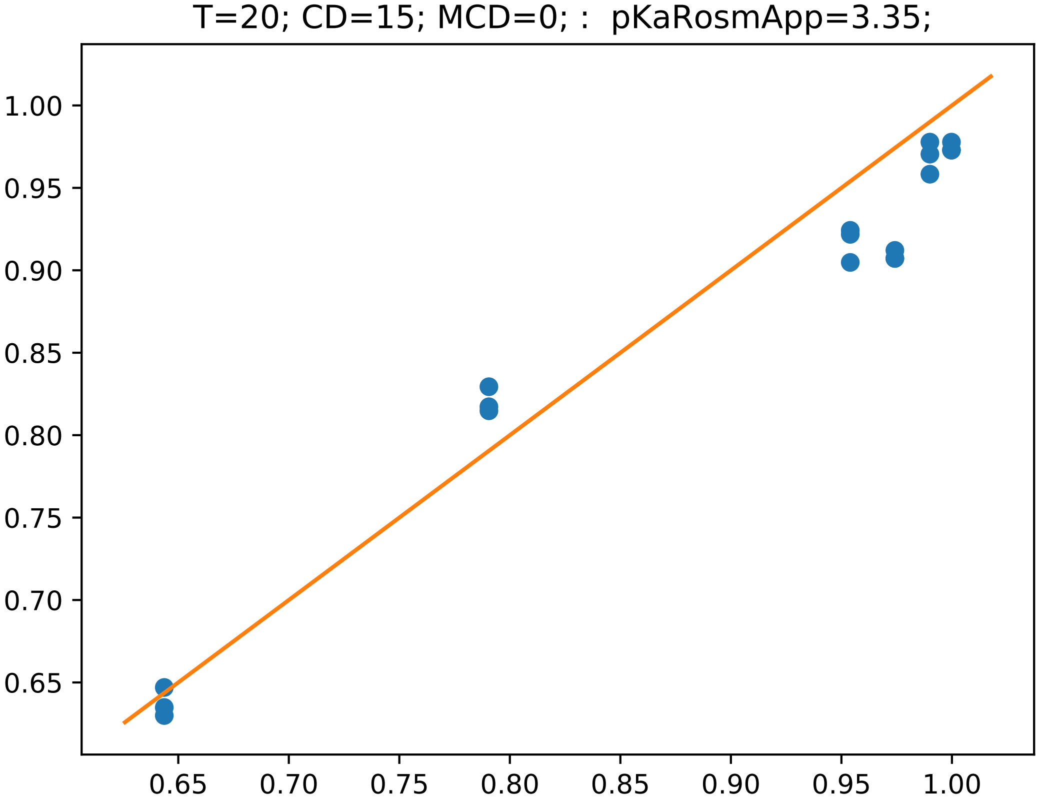

T=20; CD=15; MCD=0; : pKaRosmApp=3.35;
1.00
0.95
0.90
0.85
0.80
0.75
0.70
0.65
0.65
0.70
0.75
0.80
0.85
0.90
0.95
1.00

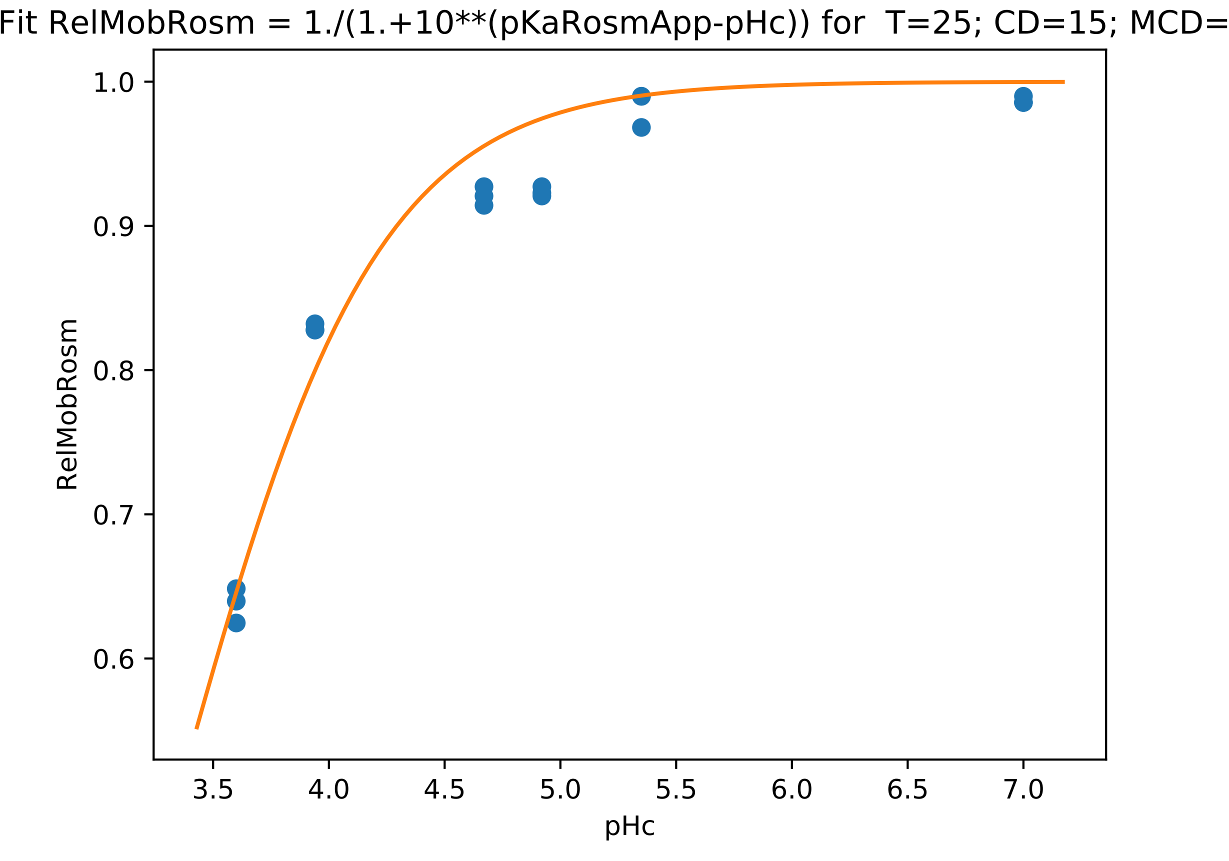

Fit RelMobRosm = 1./(1.+10**(pKaRosmApp-pHc)) for T=25; CD=15; MCD=
1.0
0.9
0.8
0.7
0.6
RelMobRosm
3.5
4.0
4.5
5.0
5.5
6.0
6.5
7.0
pHc

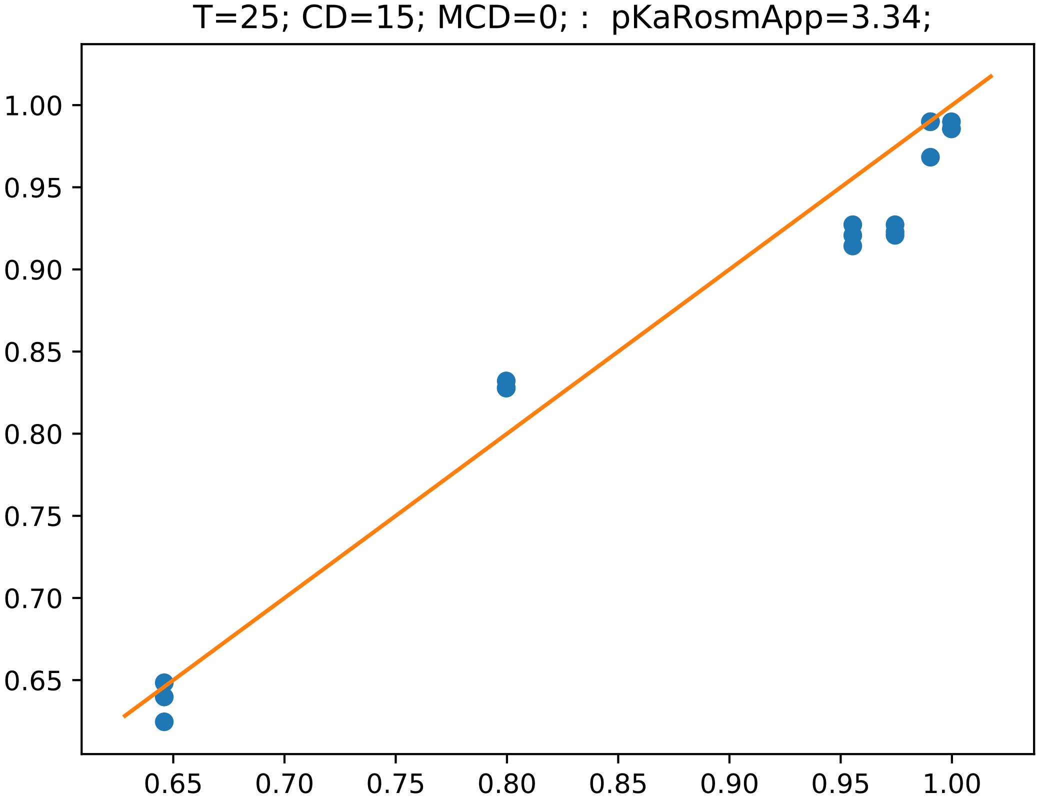
T=25; CD=15; MCD=0; : pKaRosmApp=3.34;
1.00
0.95
0.90
0.85
0.80
0.75
0.70
0.65
0.65
0.70
0.75
0.80
0.85
0.90
0.95
1.00

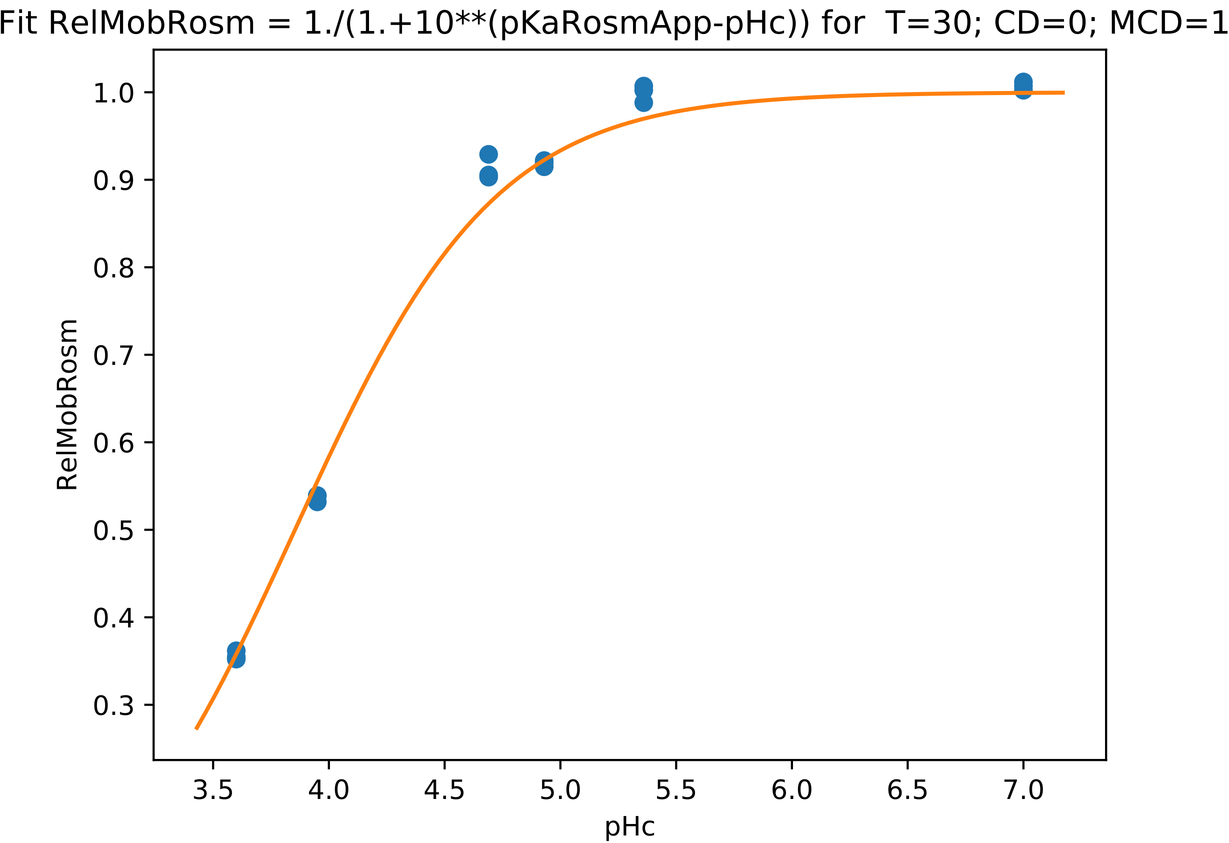
Fit RelMobRosm = 1./(1.+10**(pKaRosmApp-pHc)) for T=30; CD=0; MCD=1
1.0
0.9
0.8
0.7
0.6
0.5
0.4
0.3
RelMobRosm
3.5
4.0
4.5
5.0
5.5
6.0
6.5
7.0
pHc

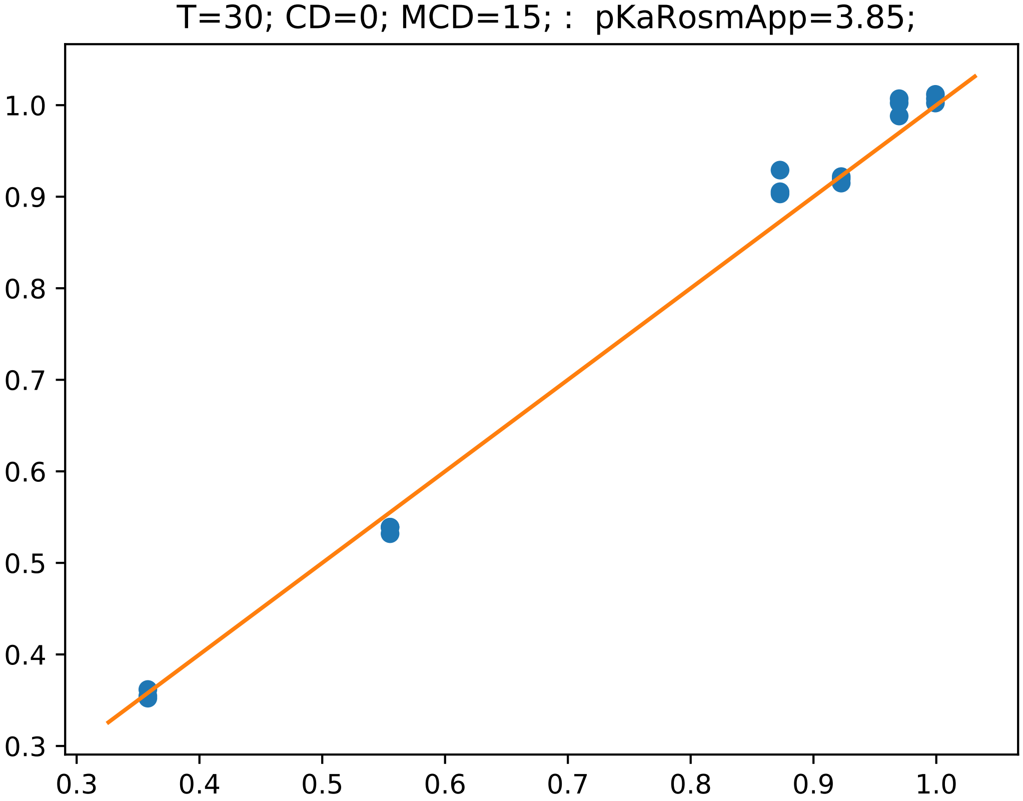
T=30; CD=0; MCD=15; : pKaRosmApp=3.85;
1.0
0.9
0.8
0.7
0.6
0.5
0.4
0.3
0.3
0.4
0.5
0.6
0.7
0.8
0.9
1.0

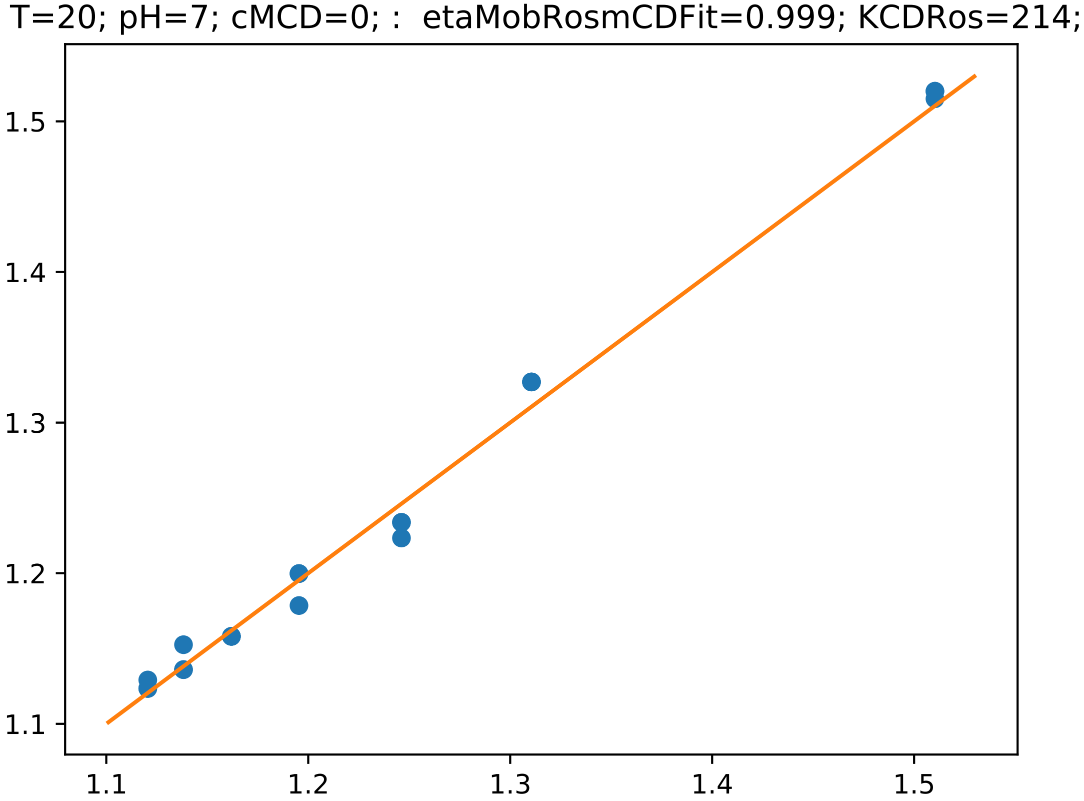
T=20; pH=7; cMCD=0; : etaMobRosmCDFit=0.999; KCDRos=214;
1.5
1.4
1.3
1.2
1.1
1.1
1.2
1.3
1.4
1.5

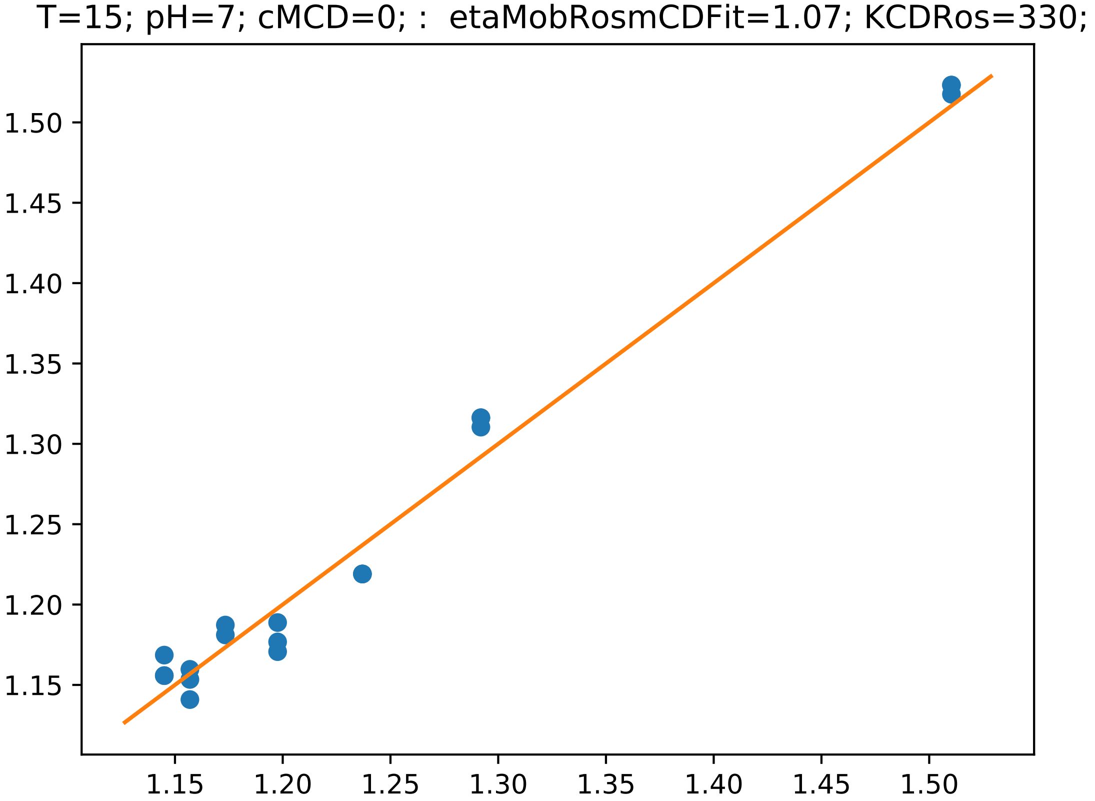

T=15; pH=7; cMCD=0; : etaMobRosmCDFit=1.07; KCDRos=330;
1.50
1.45
1.40
1.35
1.30
1.25
1.20
1.15
1.15
1.20
1.25
1.30
1.35
1.40
1.45
1.50

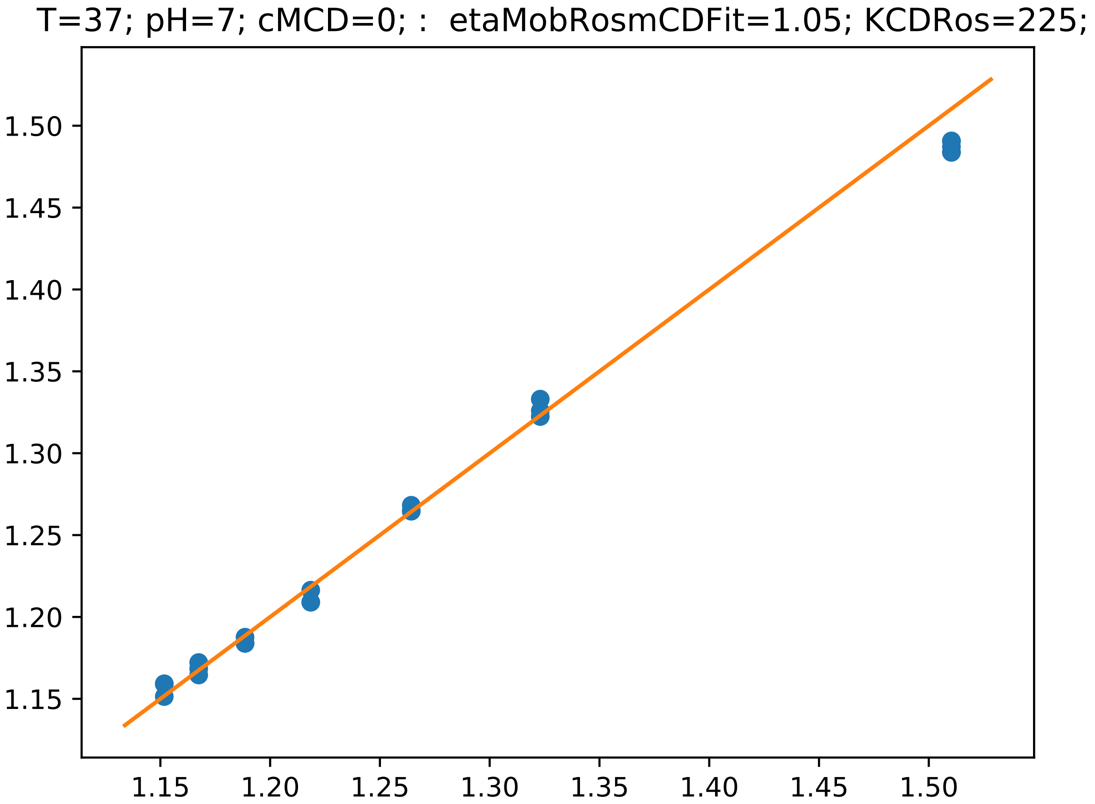

T=37; pH=7; cMCD=0; : etaMobRosmCDFit=1.05; KCDRos=225;
1.50
1.45
1.40
1.35
1.30
1.25
1.20
1.15
1.15
1.20
1.25
1.30
1.35
1.40
1.45
1.50

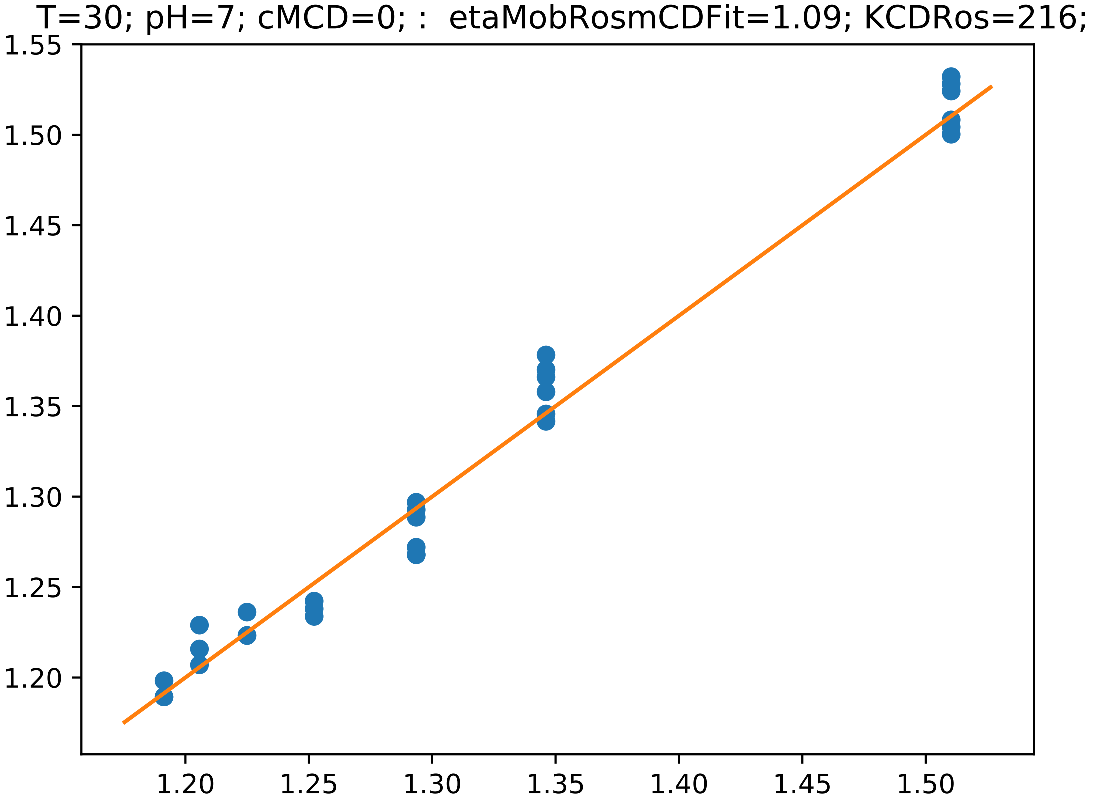
T=30; pH=7; cMCD=0; : etaMobRosmCDFit=1.09; KCDRos=216;
1.55
1.50
1.45
1.40
1.35
1.30
1.25
1.20
1.20
1.25
1.30
1.35
1.40
1.45
1.50

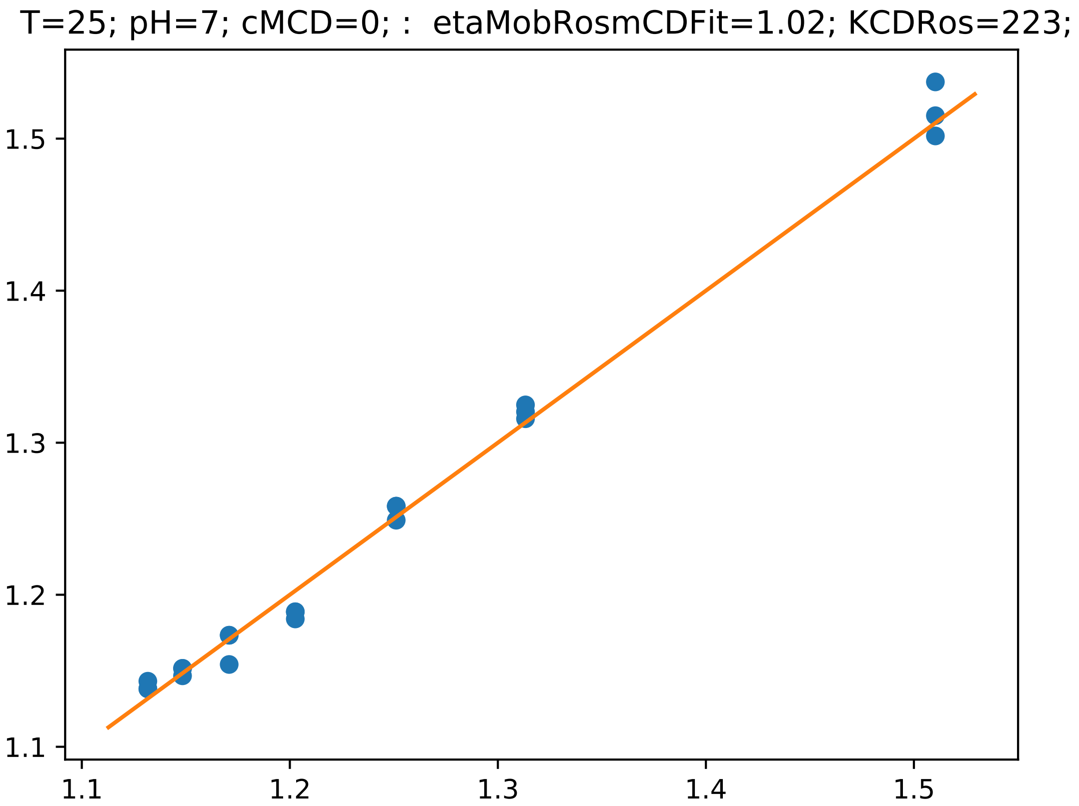
T=25; pH=7; cMCD=0; : etaMobRosmCDFit=1.02; KCDRos=223;
1.5
1.4
1.3
1.2
1.1
1.1
1.2
1.3
1.4
1.5

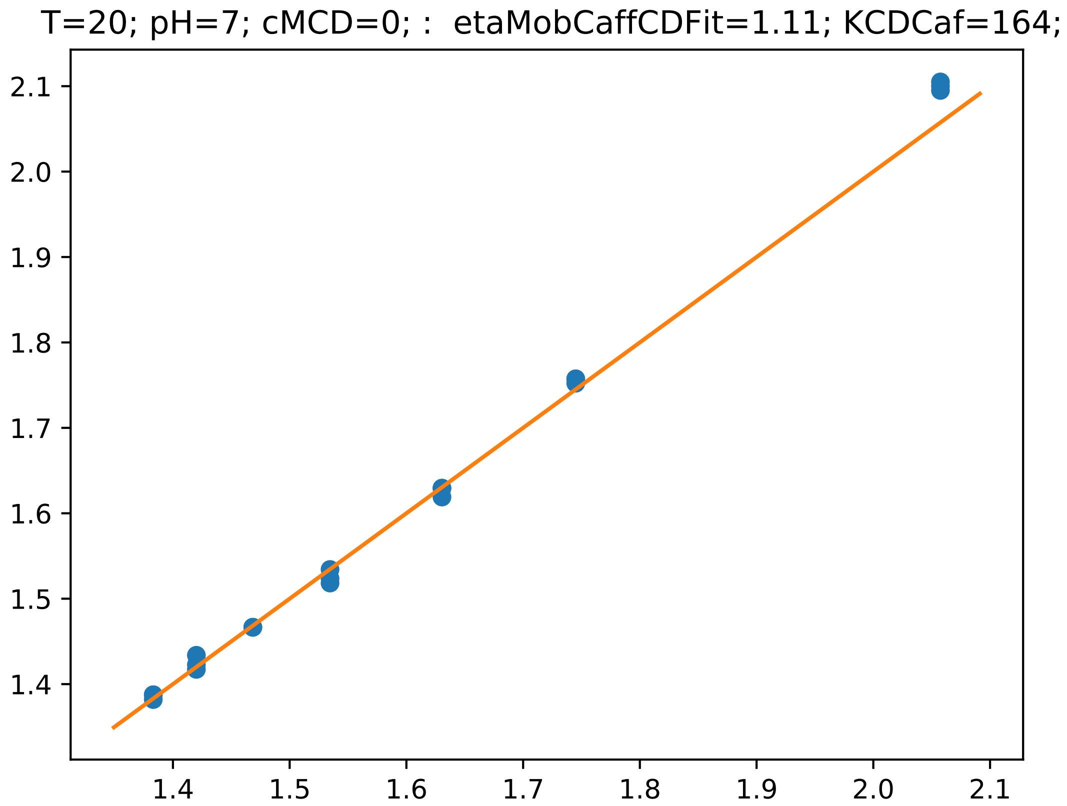
T=20; pH=7; cMCD=0; : etaMobCaffCDFit=1.11; KCDCaf=164;
2.1
2.0
1.9
1.8
1.7
1.6
1.5
1.4
1.4
1.5
1.6
1.7
1.8
1.9
2.0
2.1

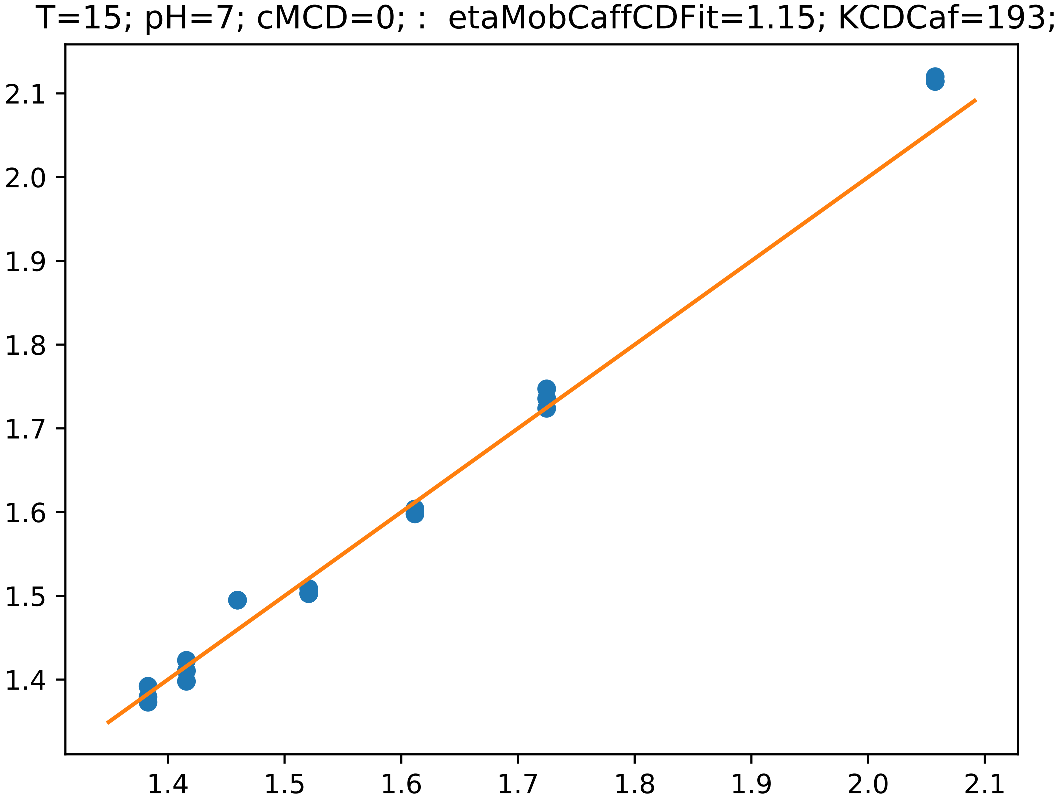

T=15; pH=7; cMCD=0; : etaMobCaffCDFit=1.15; KCDCaf=193;
2.1
2.0
1.9
1.8
1.7
1.6
1.5
1.4
1.4
1.5
1.6
1.7
1.8
1.9
2.0
2.1

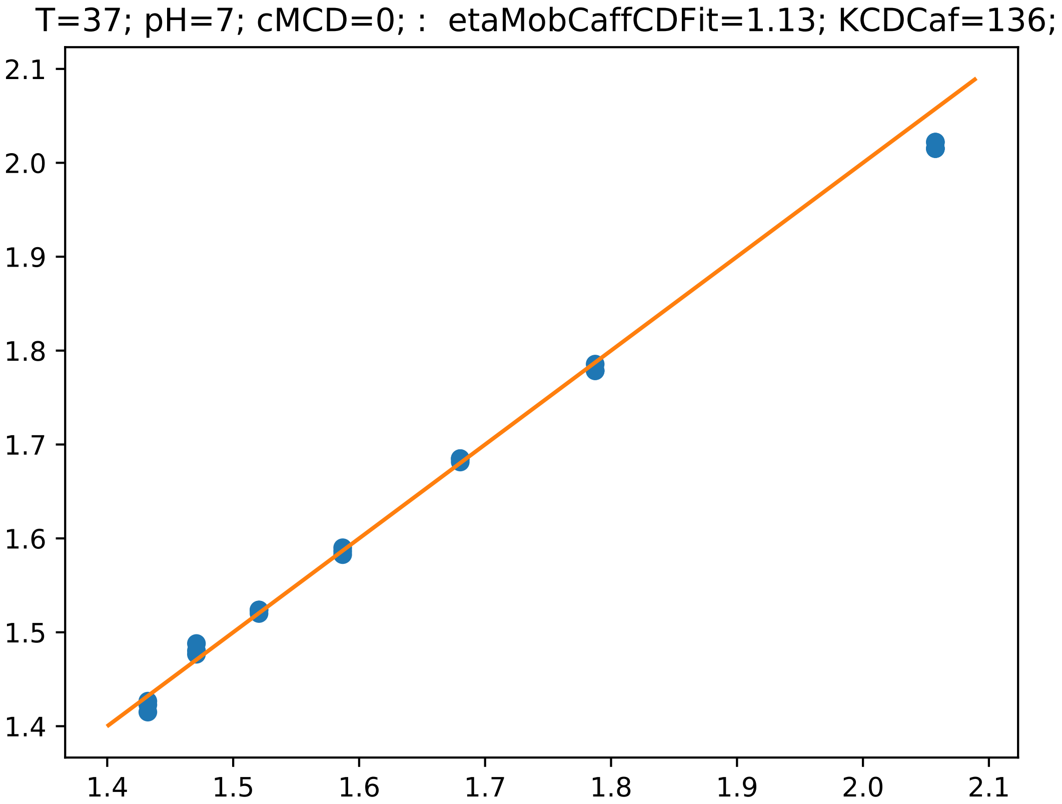
T=37; pH=7; cMCD=0; : etaMobCaffCDFit=1.13; KCDCaf=136;
2.1
2.0
1.9
1.8
1.7
1.6
1.5
1.4
1.4
1.5
1.6
1.7
1.8
1.9
2.0
2.1

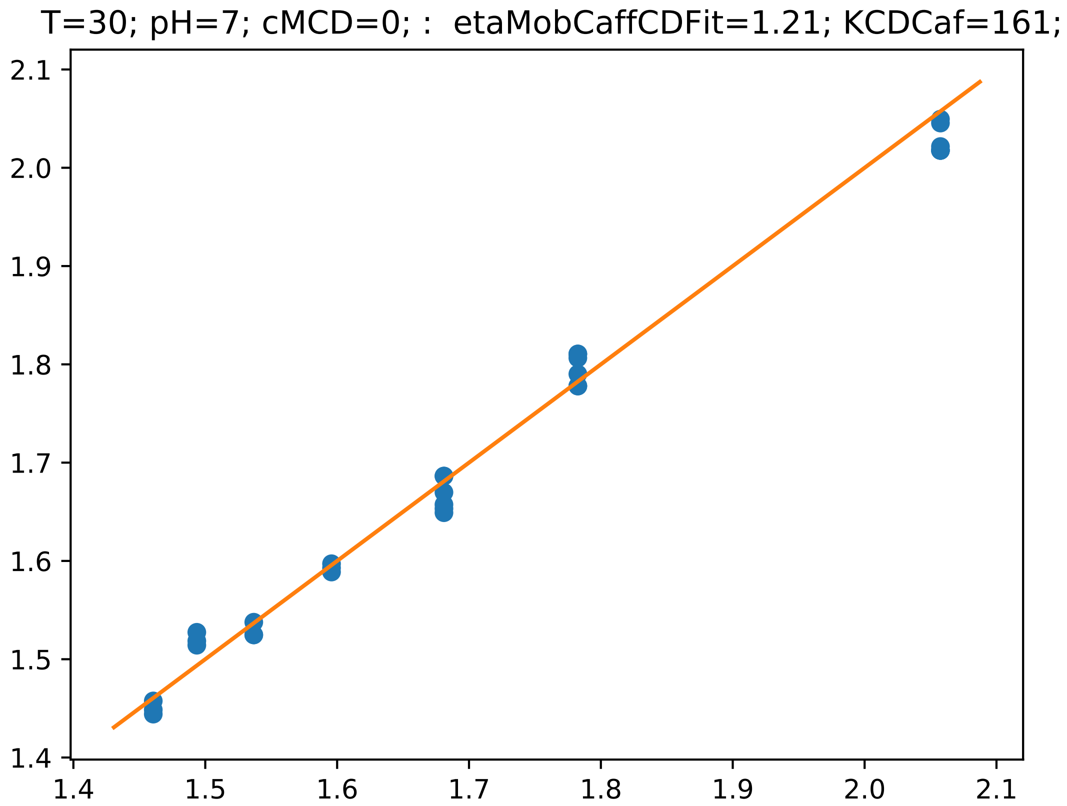

T=30; pH=7; cMCD=0; : etaMobCaffCDFit=1.21; KCDCaf=161;
2.1
2.0
1.9
1.8
1.7
1.6
1.5
1.4
1.4
1.5
1.6
1.7
1.8
1.9
2.0
2.1

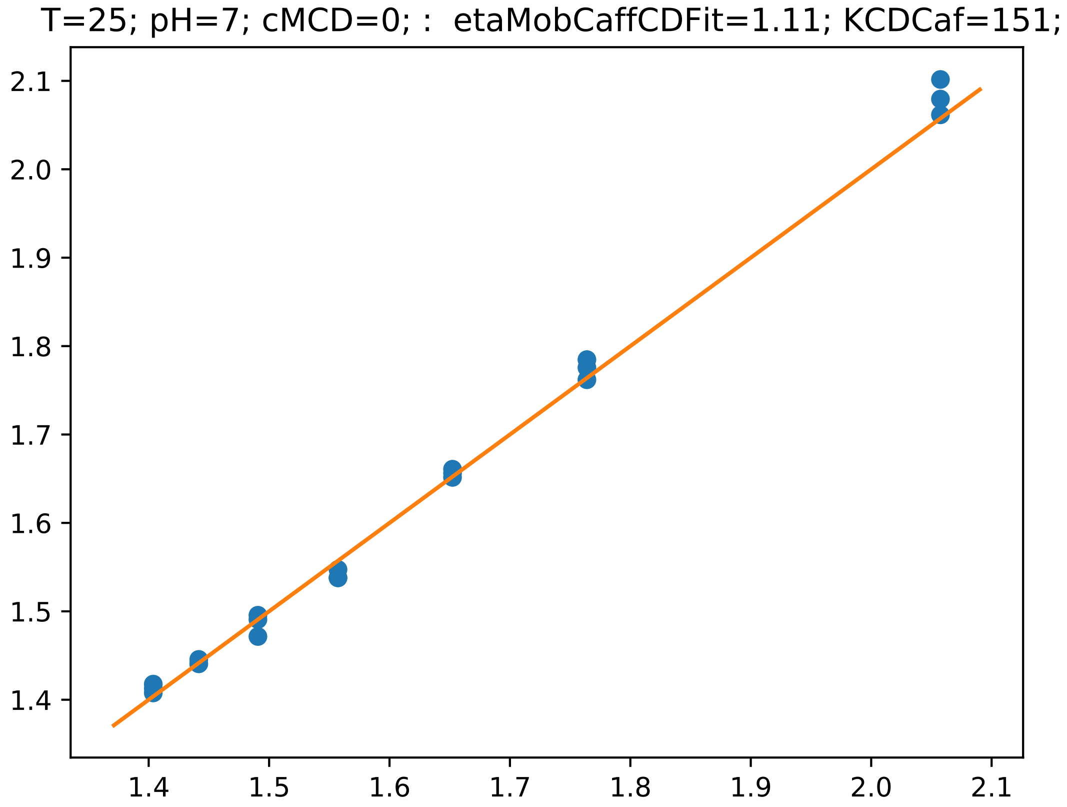
T=25; pH=7; cMCD=0; : etaMobCaffCDFit=1.11; KCDCaf=151;
2.1
2.0
1.9
1.8
1.7
1.6
1.5
1.4
1.4
1.5
1.6
1.7
1.8
1.9
2.0
2.1

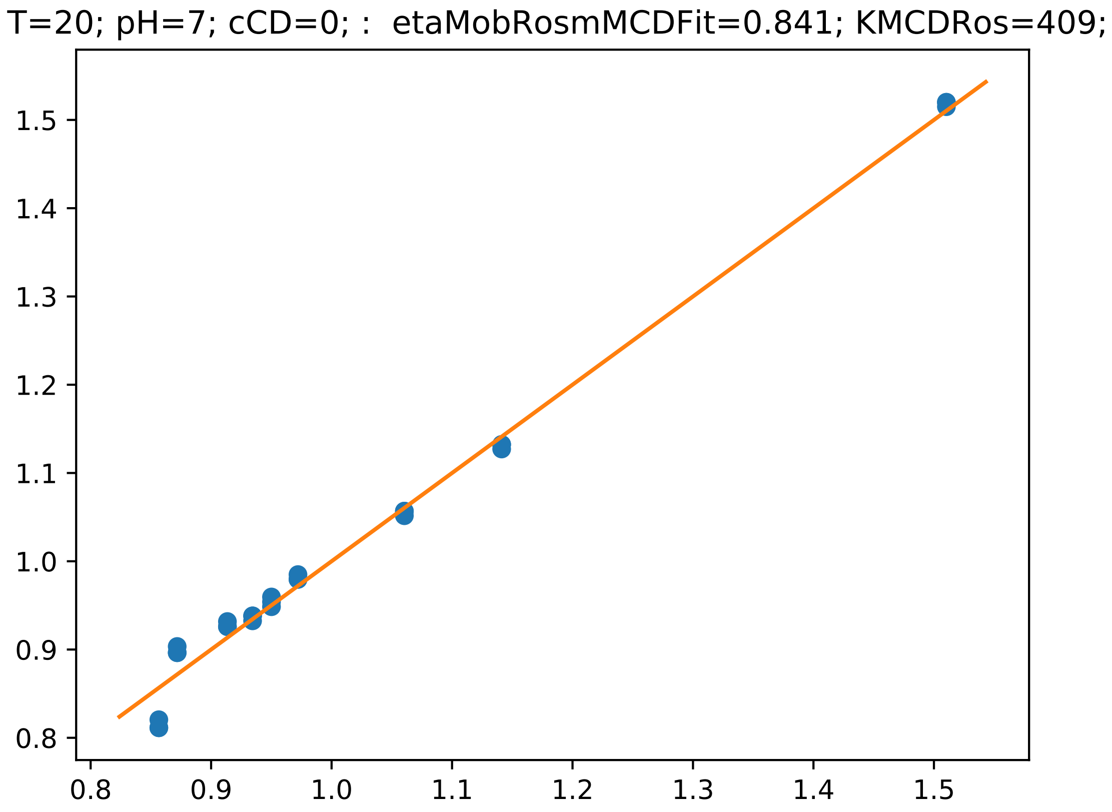

T=20; pH=7; cCD=0; : etaMobRosmMCDFit=0.841; KMCDRos=409;
1.5
1.4
1.3
1.2
1.1
1.0
0.9
0.8
0.8
0.9
1.0
1.1
1.2
1.3
1.4
1.5

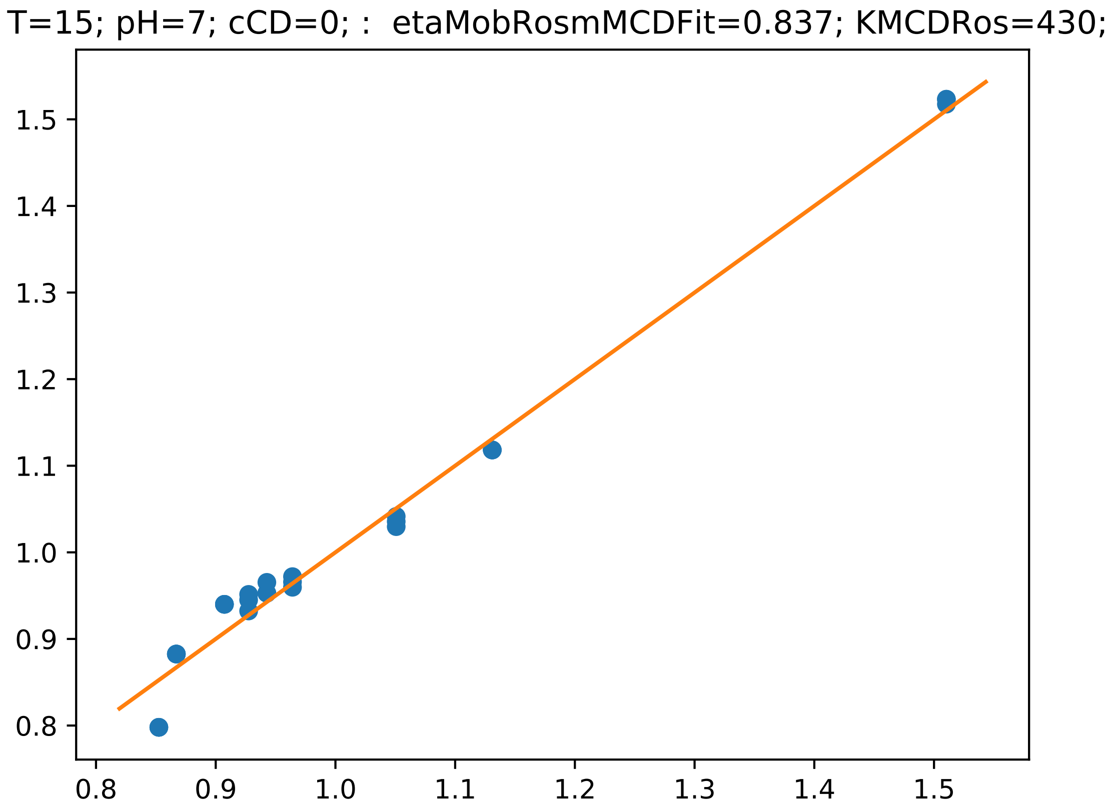
T=15; pH=7; cCD=0; : etaMobRosmMCDFit=0.837; KMCDRos=430;
1.5
1.4
1.3
1.2
1.1
1.0
0.9
0.8
0.8
0.9
1.0
1.1
1.2
1.3
1.4
1.5

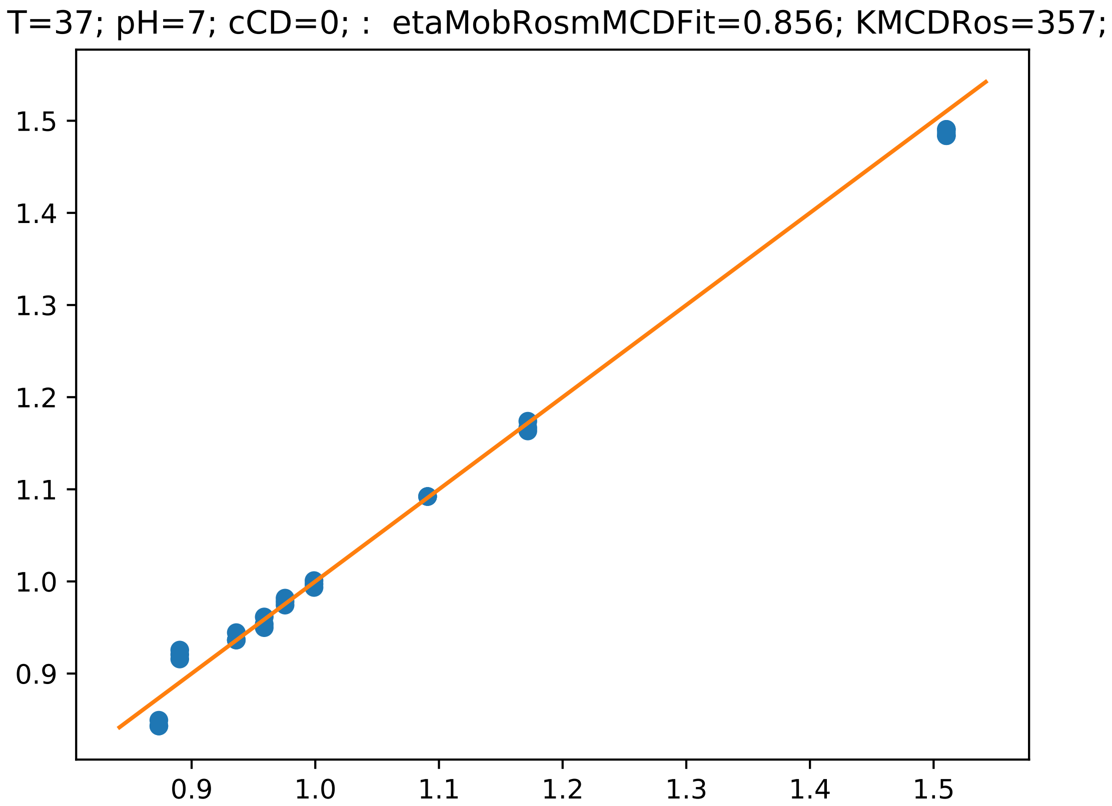
T=37; pH=7; cCD=0; : etaMobRosmMCDFit=0.856; KMCDRos=357;
1.5
1.4
1.3
1.2
1.1
1.0
0.9
0.9
1.0
1.1
1.2
1.3
1.4
1.5

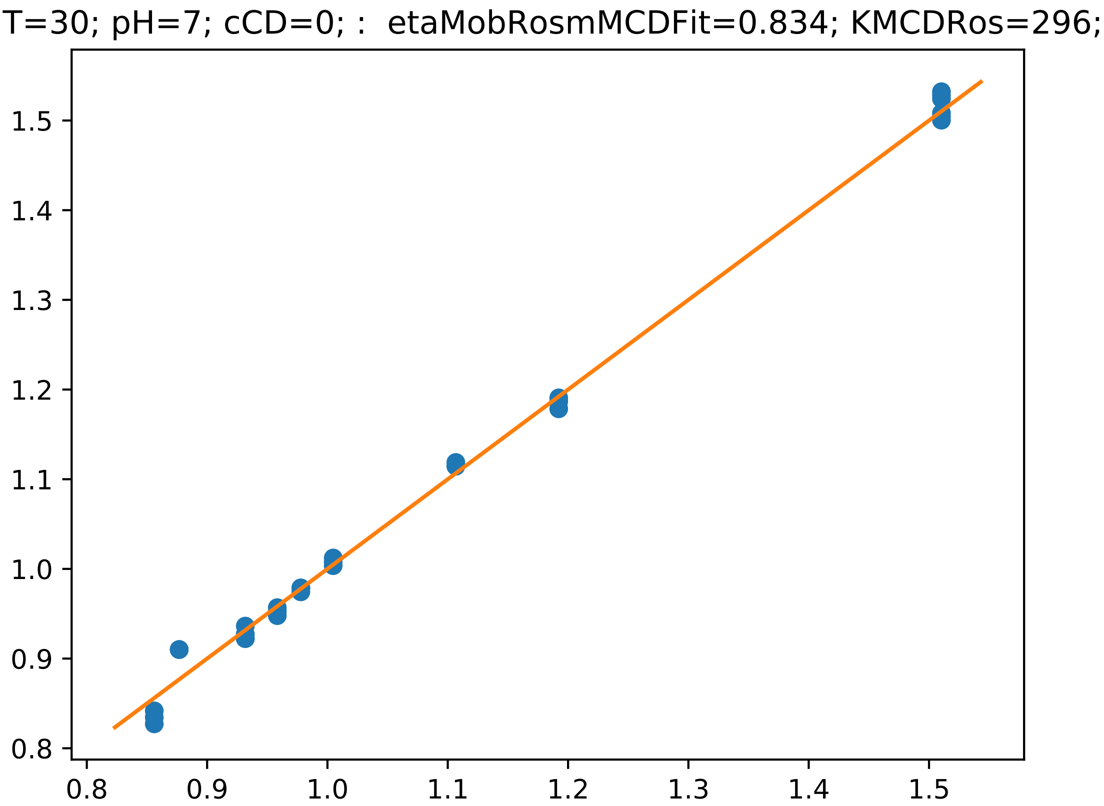
T=30; pH=7; cCD=0; : etaMobRosmMCDFit=0.834; KMCDRos=296;
1.5
1.4
1.3
1.2
1.1
1.0
0.9
0.8
0.8
0.9
1.0
1.1
1.2
1.3
1.4
1.5

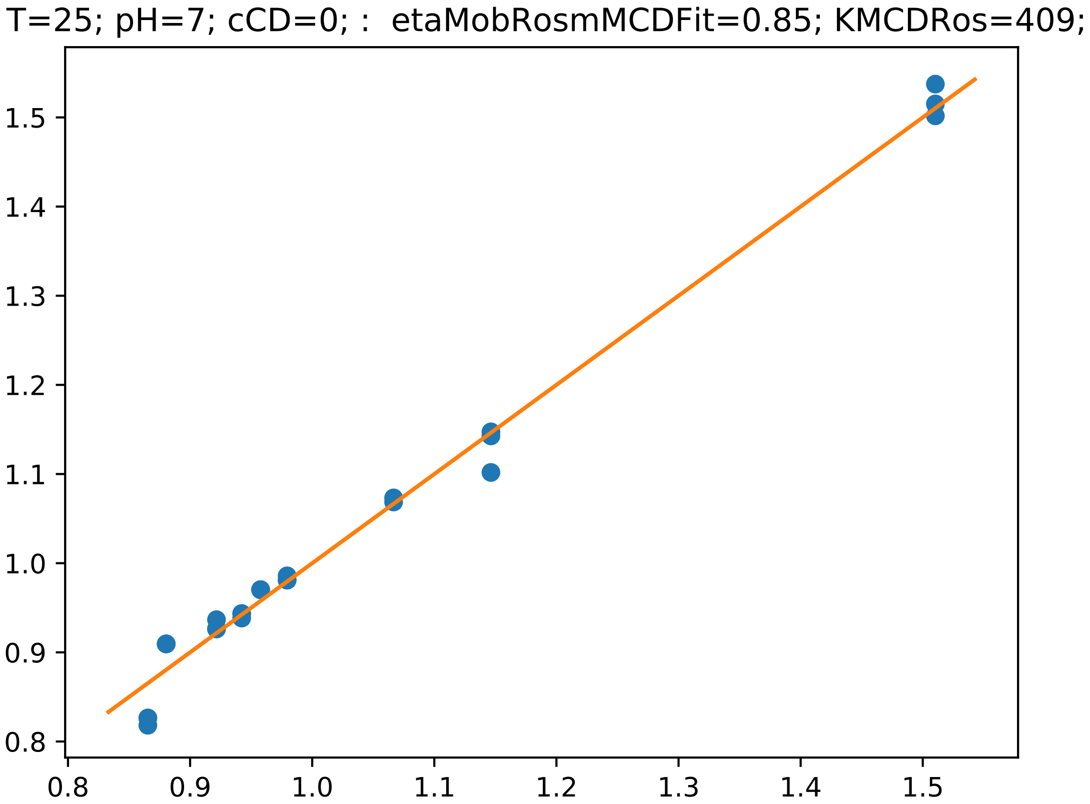
T=25; pH=7; cCD=0; : etaMobRosmMCDFit=0.85; KMCDRos=409;
0.8
0.9
1.0
1.1
1.2
1.3
1.4
1.5

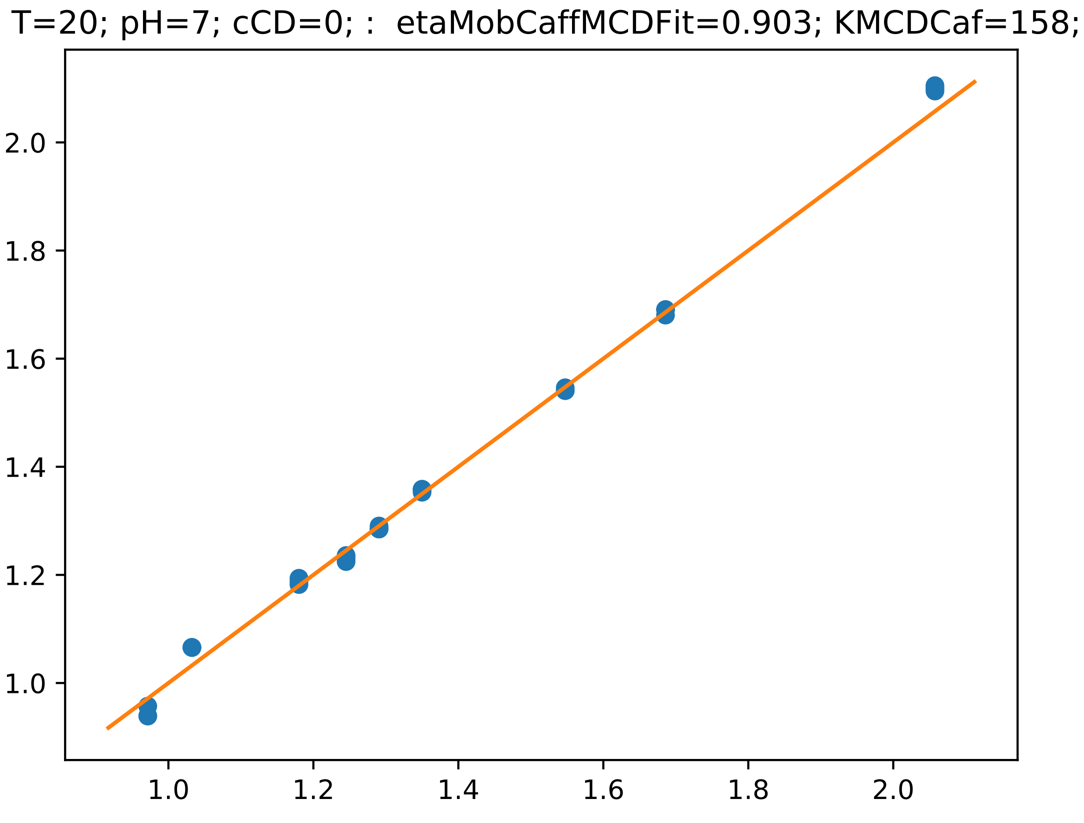

T=20; pH=7; cCD=0; : etaMobCaffMCDFit=0.903; KMCDCaf=158;
2.0
1.8
1.6
1.4
1.2
1.0
1.0
1.2
1.4
1.6
1.8
2.0

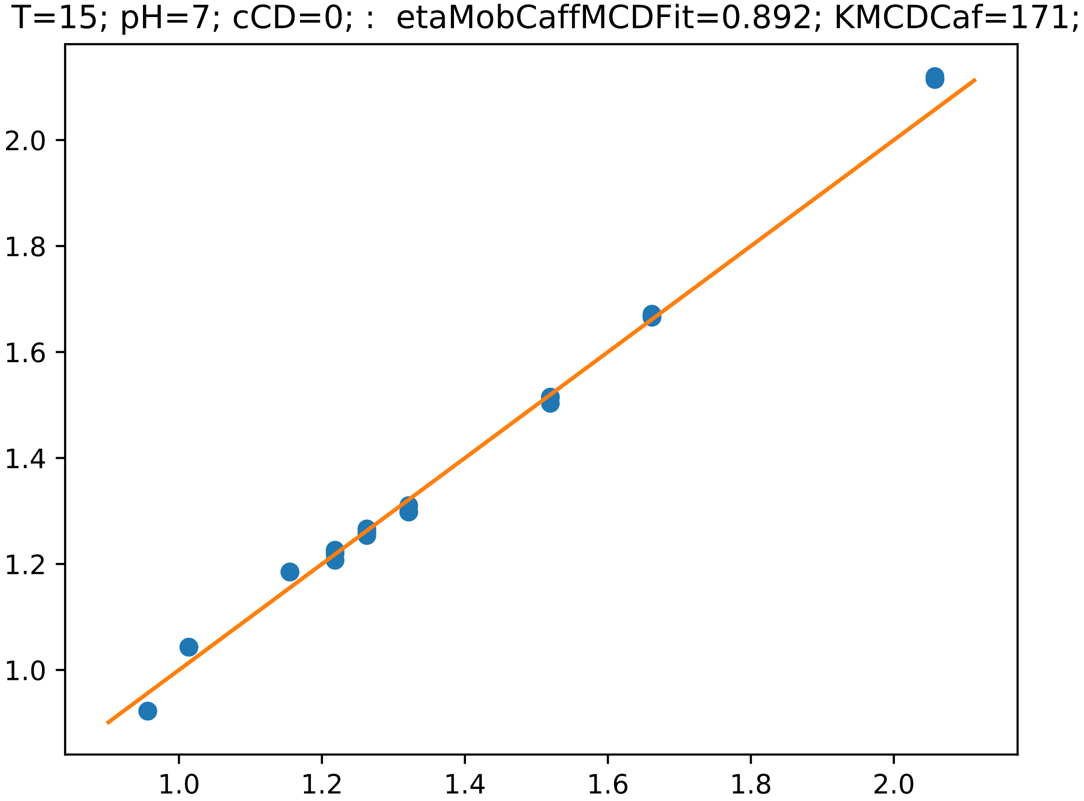
T=15; pH=7; cCD=0; : etaMobCaffMCDFit=0.892; KMCDCaf=171;
2.0
1.8
1.6
1.4
1.2
1.0
1.0
1.2
1.4
1.6
1.8
2.0

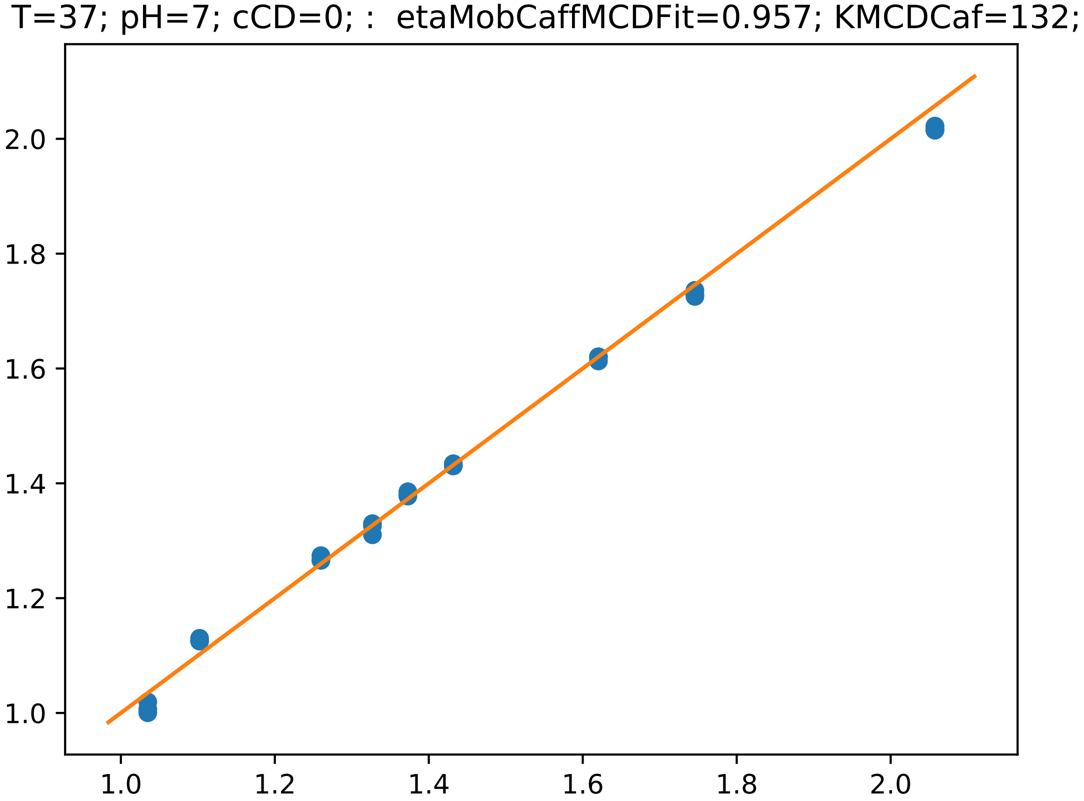
T=37; pH=7; cCD=0; : etaMobCaffMCDFit=0.957; KMCDCaf=132;
2.0
1.8
1.6
1.4
1.2
1.0
1.0
1.2
1.4
1.6
1.8
2.0

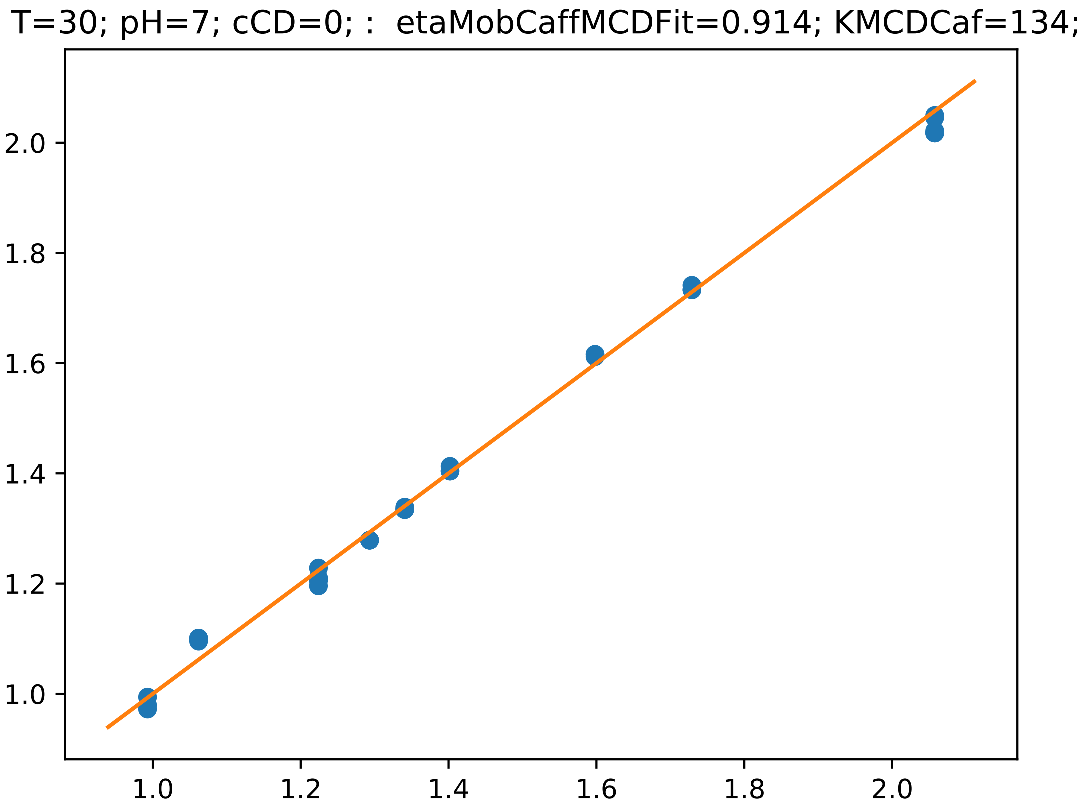
T=30; pH=7; cCD=0; : etaMobCaffMCDFit=0.914; KMCDCaf=134;
2.0
1.8
1.6
1.4
1.2
1.0
1.0
1.2
1.4
1.6
1.8
2.0

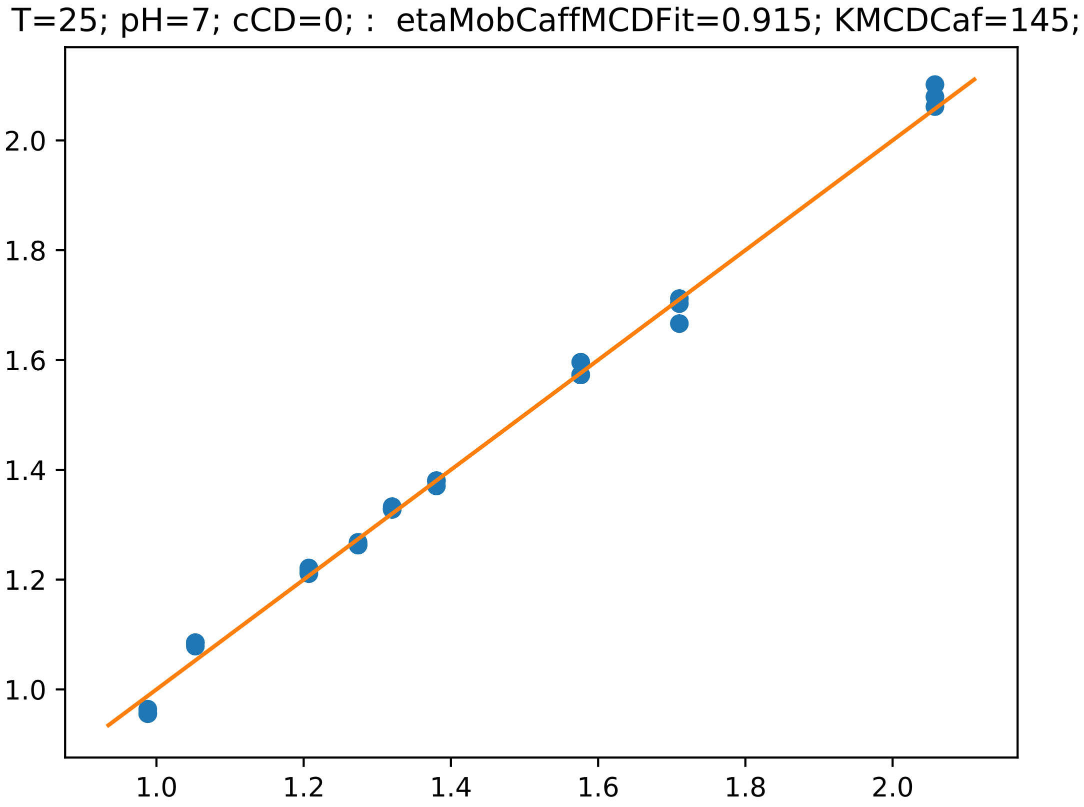

T=25; pH=7; cCD=0; : etaMobCaffMCDFit=0.915; KMCDCaf=145;
2.0
1.8
1.6
1.4
1.2
1.0
1.0
1.2
1.4
1.6
1.8
2.0

Caff0=-2.16; lCaff=0.006; pKpCaff0=-2.58; lpCaff=0.0114; uacMCaff=0.954;

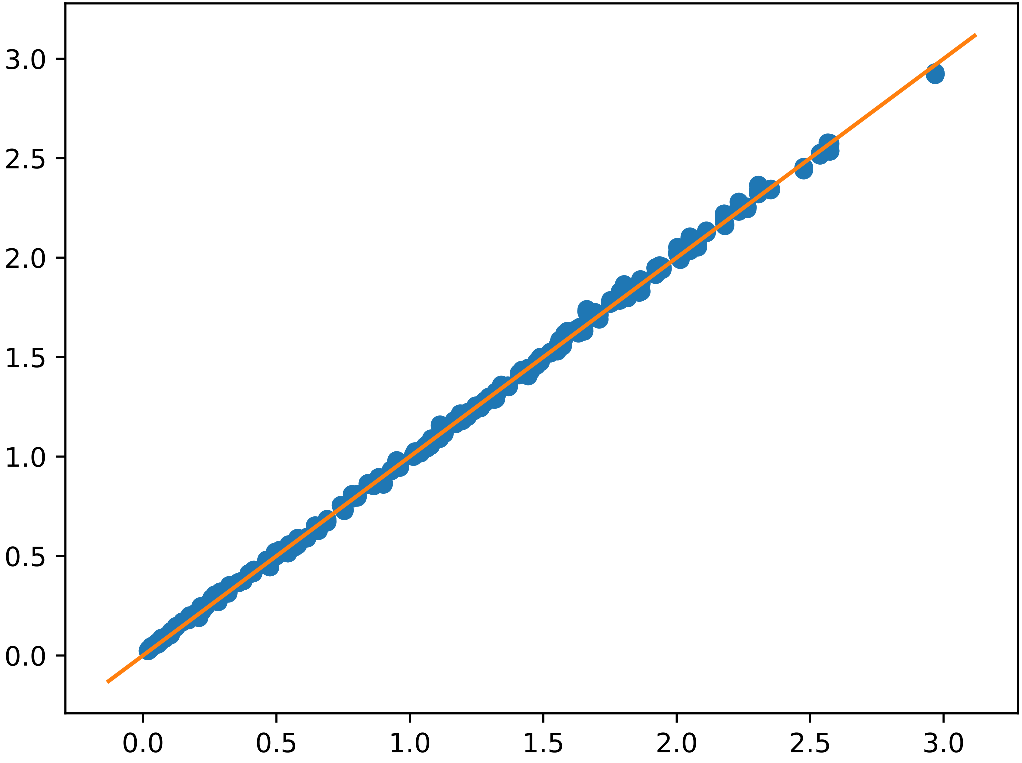

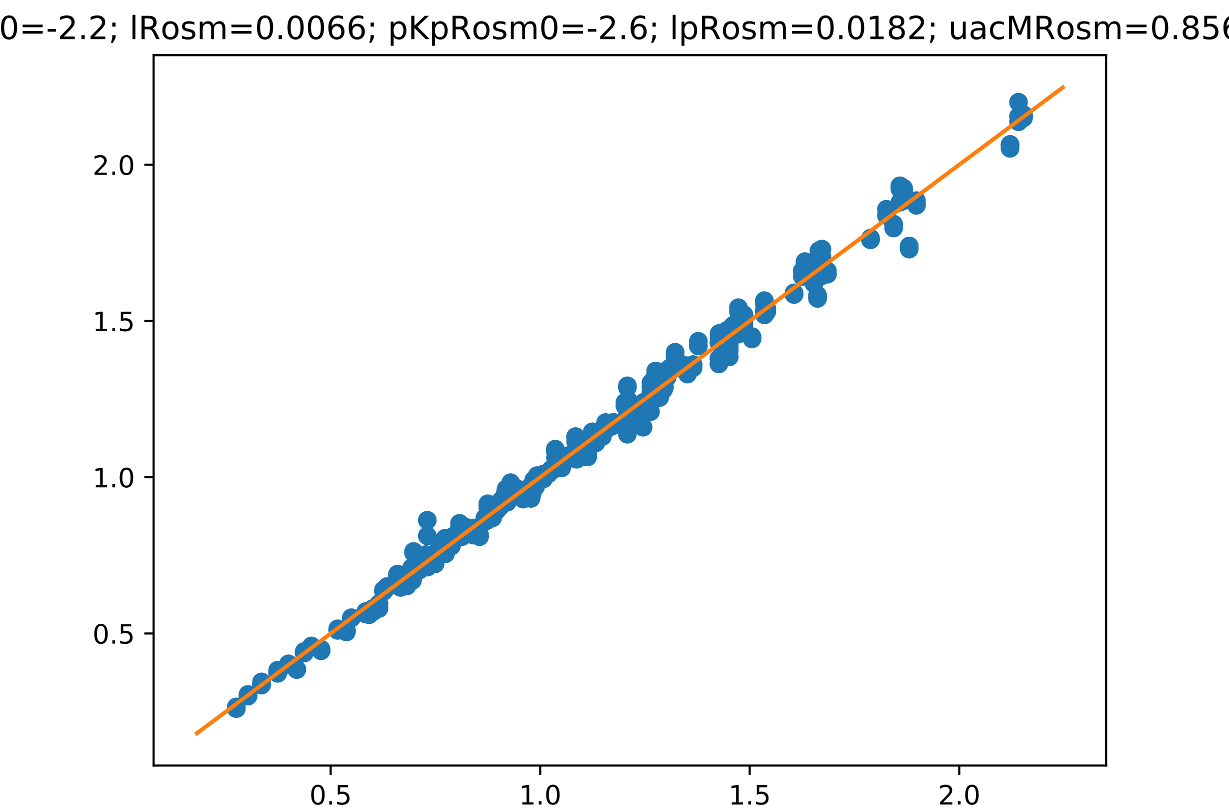
0=-2.2; lRosm=0.0066; pKpRosm0=-2.6; lpRosm=0.0182; uacMRosm=0.856
2.0
1.5
1.0
0.5
0.5
1.0
1.5
2.0
